# Characterizing the epidemiology of *Mycoplasma pneumoniae* infections in China in 2022–2024: a nationwide cross-sectional study of over 1.6 million cases

**DOI:** 10.1080/22221751.2025.2482703

**Published:** 2025-03-27

**Authors:** Yamin Sun, Pei Li, Ronghua Jin, Yaoming Liang, Jiale Yuan, Zhongxin Lu, Junrong Liang, Yingmiao Zhang, Hongyu Ren, Yuanyuan Zhang, Jianchun Chen, Yun Huang, Chuixu Lin, Yinghua Li, Jianfeng Zhou, Xi Wang, You Li, Senzhong Huang, Jianguo Xu, Tian Qin

**Affiliations:** a National Key Laboratory of Intelligent Tracking and Forecasting for infectious Diseases, Beijing Ditan Hospital, Capital Medical University, Beijing, People’s Republic of China; b Beijing Key Laboratory of Viral Infectious Disease, Beijing Institute of Infectious Diseases, Beijing, People’s Republic of China; c National Center for Infectious Diseases, Beijing Ditan Hospital, Capital Medical University, Beijing, People’s Republic of China; d KingMed Diagnostics, Guangzhou KingMed Diagnostics Group Co., Ltd, Guangzhou, People’s Republic of China; e National Key Laboratory of Intelligent Tracking and Forecasting for Infectious Diseases, TEDA Institute of Biological Sciences and Biotechnology, Nankai University, Tianjin, People’s Republic of China; f The Central Hospital of Wuhan, Tongji Medical College, Huazhong University of Science and Technology, Wuhan, People’s Republic of China; g National Key Laboratory of Intelligent Tracking and Forecasting for Infectious Diseases, National Institute for Communicable Disease Control and Prevention, Chinese Center for Disease Control and Prevention, Beijing, People’s Republic of China; h Department of Epidemiology, National Vaccine Innovation Platform, School of Public Health, Nanjing Medical University, Nanjing, People’s Republic of China; i National Key Laboratory of Intelligent Tracking and Forecasting for Infectious Diseases, School of Statistics and Data Science, Nankai University, Tianjin, People's Republic of China

**Keywords:** *Mycoplasma pneumoniae*, macrolide-resistant, transmission zones, age- and sex- specific, China

## Abstract

*Mycoplasma pneumoniae* (MP) is a leading cause of community-acquired pneumonia (CAP), accounting for 10–40% of cases in children. In China, the high prevalence of macrolide-resistant MP (MRMP) and recurrent MP epidemics place a significant burden on the healthcare system. Leveraging data from over 1.6 million cases, this study provides a comprehensive analysis of the epidemiological characteristics of MP across China. Seasonal patterns analysis revealed three distinct transmission zones in China. Transmission Zone 1 exhibited two annual epidemic peaks, while Zones 2 and 3 showed a single annual peak of distinct timings. Notably, winter travel to popular tourist destinations appears to influence MP infection patterns in China. Age- and sex- specific analysis indicated male newborns aged [0–1) years face a 1.67 times higher risk of MP infection compared to females. Conversely, females aged [23–38) years have a higher infection risk, likely due to their caregiving roles. The proportion of MRMP surged from 80.00% to 93.02% between July 2023 and May 2024, with a median growth rate of 10.21%. This rapid increase contrasts sharply with the modest 5.3% rise observed from 2011 to 2019, and we attribute this escalation in part to the growing prevalence of the T1-3R clade strain in China. These findings have important implications for the identification of high-risk population, place, and time for more targeted efforts of prevention and treatment. Furthermore, the rapidly increased proportion of MRMP in the 2023–2024 season raises a concerning signal regarding antibiotic use.

## Introduction

*Mycoplasma pneumoniae* (MP) is one of the smallest self-replicating organisms without a cell wall, which makes them intrinsically resistant to β-lactams and to all cell-wall targeting antimicrobials [1,2]. Among children, MP accounts for 10–40% of Community-acquired pneumonia (CAP) cases, consistently ranking as a leading cause of morbidity and mortality [3,4]. According to the Global Burden of Disease (GBD) 2021 Lower Respiratory Infections and Antimicrobial Resistance Collaborators study, *Mycoplasma* spp was responsible for 25.3 million lower respiratory tract infections (LRTIs) in 2021, making it the third most common pathogen contributing to LRTIs globally [5]. The COVID-19 pandemic saw a reduction in MP infections due to widespread non-pharmaceutical interventions (NPIs). However, with the relaxation of these measures, a resurgence of MP cases has been observed in countries including China, Denmark, France, the Netherlands, and Spain [6–10].

In recent years, the rising proportion of macrolide-resistant MP (MRMP) in China has posed significant challenges to clinical treatment [11–14]. In 2023, China experienced a severe MP epidemic that overwhelmed emergency departments and drew the attention of the World Health Organization (WHO) [15,16]. Reports from various regions, including Beijing, Zhejiang, Wuhan, Shanghai, Inner Mongolia, and Northeast China, have highlighted the prevalence of MP during this period [14,17–22]. The widespread MRMP and the scale of the epidemic present severe public health challenges for China. However, national epidemiological characteristics in China is lacking.

Targeted next-generation sequencing (tNGS) has proven to be highly effective in identifying hard-to-culture pathogens and monitoring drug resistance genes. It is increasingly being adopted in clinical settings for pathogens such as *Mycobacterium tuberculosis* and *Chlamydia psittaci* [23–26]. Recognizing its potential, the WHO endorsed tNGS for drug-resistant tuberculosis testing in March 2024 and has prioritized its integration into tuberculosis diagnostic tool research and development. With the decreasing costs of tNGS, it has become a primary tool for identifying pathogen spectra in acute respiratory infections (ARIs) in many hospitals across China [27–30].

In this study, we analyzed tNGS data from the nationwide hospital laboratory network of KingMed Diagnostics, which has established 39 medical center laboratories across China, serving over 25,000 hospitals or healthcare centers covering regions where over 90% of the population resides. Based on the 1.6 million ARI cases diagnosed by tNGS nationwide, we precisely characterized the geographic, seasonal, and demographic features of MP epidemics in China, and identified the factors driving the surge in MP macrolide resistance.

## Materials and methods

### Study design

This cross-sectional study utilized tNGS diagnostic data from hospitalized patients with acute respiratory infections (ARIs), provided by KingMed (Guangzhou, Guangdong, China), a diagnostic company offering third-party testing services to over 25,000 healthcare institutions across China. The data were collected from January 2022 to May 2024, covering 4,758 hospitals in 314 cities across all 31 provinces of mainland China. For the analysis, data from only 29 provinces were included, excluding Qinghai and Tibet due to fewer than 2,000 total cases reported in these regions (Supplemental Figure S1).

The data analysis protocol was reviewed and approved by the ethics review committees of KingMed (2024139), the National Institute of Communicable Disease Prevention and Control, China CDC (ICDC-202115), and The Central Hospital of Wuhan (WHZXKYL2023-171). From April 2023 to March 2024, a total of 1,221,943 ARI samples were collected and analyzed for seasonal pattern analysis. Age- and sex-specific risk assessments were performed on 1,668,960 ARI samples collected from January 2022 to May 2024. Beginning in July 2023, all samples were also tested for four macrolide-resistant sites (A2063G, A2064G, C2617G, and A2067G) in MP 23S rRNA, resulting in the inclusion of 1,211,183 ARI samples for macrolide resistance analysis from July 2023 to May 2024. Detailed descriptions and rationale for the various datasets are provided in the supplemental material (Supplemental Figure S2).

**Figure 1.**
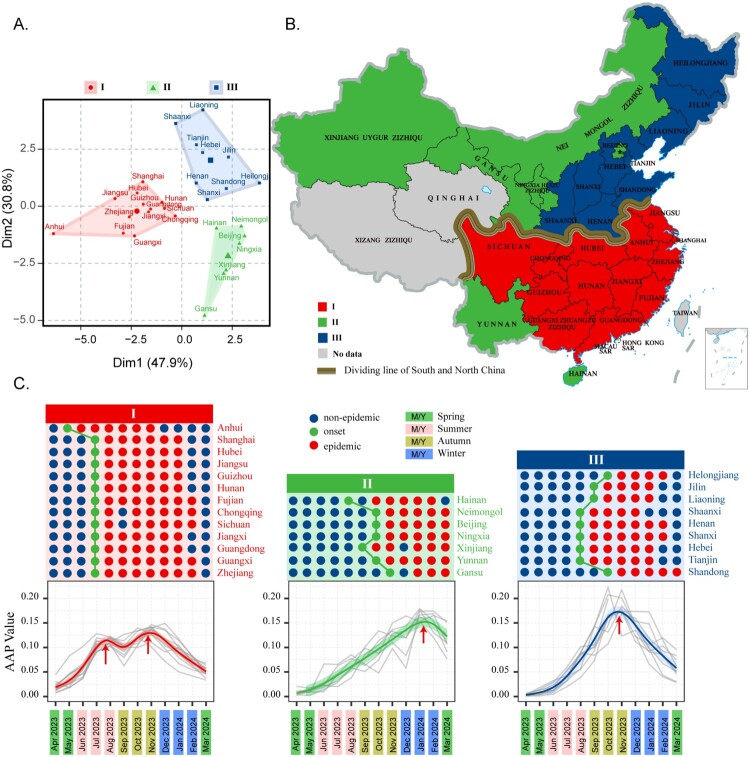
Seasonal pattern and transmission zones of MP in 29 provinces. (a) K-means clustering results of epidemic trends in different provinces, Dim1 and Dim2 represent the first and second dimensions after dimensionality reduction, respectively, where Dim1 explains the largest variance (47.9%) in the data, and Dim2 explains the second-largest variance (30.8%). (b) Cluster distribution on the map of China. (c) Onset months (green dots), epidemic months (red dots), and non-epidemic months (blue dots) in different provinces and clusters. Gray lines represent the AAP trend charts for different provinces, the fitted line represents the epidemic trend for clusters 1 (red), 2 (green), and 3 (blue). Red arrows indicate epidemic peaks.

Furthermore, 253 MP-positive samples from Wuhan were used to analyze the clade distribution of circulating MP strains in China. Of these, two isolates underwent whole-genome sequencing, while the remaining 251 samples were subjected to metagenomic next-generation sequencing (mNGS). The geographic division between southern and northern provinces of China was delineated by the Qinling Mountains and the Yellow River, as shown in Figure 1.

### Procedures

For the tNGS samples, data on sex, name, date of birth, sampling date, location (e.g. Guangdong, Guangzhou), sampling site (e.g. nasopharyngeal), and type of infection were obtained from sample submission forms. Samples missing any of that information were excluded from the study. Samples with infection types classified as ARI were included in this study. Samples with the same sex, name, date of birth, and sampling location were identified as duplicates and excluded from the study. Sputum and bronchoalveolar lavage fluid samples were classified as lower respiratory tract (LRT) samples and were analyzed using RP100™ Respiratory Pathogen Microorganisms Multiplex Testing Kits (KingCreate, China) (Supplemental Table S1). Nasopharyngeal and oropharyngeal swabs were classified as upper respiratory tract (URT) samples and were analyzed using URP50™ Respiratory Pathogen Microorganisms Multiplex Testing Kits (KingCreate, China) (Supplemental Table S1). Pathogen and resistance gene detection were conducted using proprietary software (KingCreate, China). Detailed methodologies and data quality control standards are provided in the Supplemental Text.

### Statistical analysis

#### Seasonal pattern analysis

Data from 29 provinces were normalized, and Average Annual Proportion (AAP) values were calculated for each province-month pair [31,32]. K-means clustering was employed to classify seasonal patterns across the provinces, with the optimal number of clusters determined using both the elbow method and gap statistic [33]. To validate the robustness of clustering analysis, we have performed a sensitivity analysis using Gaussian Mixture Models (GMM). Each cluster was defined as a transmission zone. Onset, epidemic, and non-epidemic months were identified for each province using established methods, and epidemic durations were calculated [34].

#### Age-sex specific analysis

The positive rates of MP were calculated across different ages and sexes. Chi-square tests were used to analyze differences in positive rates across sex and age groups [35]. Prior to performing the tests, expected cell counts were checked to ensure that no expected count was less than 1 and that fewer than 20% of cells had expected counts of less than 5. When these assumptions were violated, Fisher’s exact test was used as an alternative [36]. The positivity rate of MP for each age was calculated, and the risk of MP infection across various ages was assessed using the restricted cubic spline (RCS) method [37]. The age-sex specific of 15 common respiratory pathogens, including SPN, *Haemophilus influenzae* (HIN), *Staphylococcus aureus* (SA), human rhinovirus (HRV), human adenovirus (HAdV), human herpesvirus (HHV), *Klebsiella pneumoniae* (KPN), *Acinetobacter baumannii* (ABA), influenza A virus (IAV), *Moraxella catarrhalis* (MCAT), RSV, human parainfluenza virus (HPIV), human metapneumovirus (HMPV), influenza B virus (IBV), and *Bordetella pertussis* (BP), was analyzed.

#### Macrolide resistance analysis

Resistance sites were detected in each MP-positive case, with cases classified as MRMP-positive if any resistance site was present, and as MSMP otherwise. The proportion of MRMP-positive cases was calculated for each province-month pair. Two MP strains were isolated and their complete genomes were assembled using a combination of Illumina and Nanopore sequencing technologies [38–40]. Phylogenetic analysis was performed on these strains and 431 other MP genomes from public databases [41]. The clade and resistance profiles of each strain were determined, and the proportion of MRMP was calculated for each clade. Clade-specific SNPs were identified using an in-house script. Additionally, 251 throat swab samples underwent mNGS on the Illumina platform, with the clade for each MP strain determined by identifying clade-specific SNPs [42,43]. The proportion of the T1-3R clade was calculated by dividing the number of T1-3R clade samples by the total number of samples with a determined clade.

## Results

### Seasonal pattern analysis

Nationally, while MP infections occurred year-round, there was a peak from October–December, with the highest MP-positive rate among ARI cases observed in November, reaching 33.56%. Regionally, provinces such as Fujian, Anhui, Zhejiang, and Hubei showed high MP-positive rates among ARI cases, with Fujian having the highest MP-positive rate at 35.43% (Supplemental Table S3 and S4). The seasonal patterns of MP (including onset, epidemic, and non-epidemic months) in the 29 provinces of China were analyzed (Supplemental Table S5). These provinces were then categorized into three distinct transmission zones based on their seasonal patterns (Figure 1(A), Supplemental Table S6, and Figure S3). Transmission Zone 1 includes 13 provinces (Anhui, Shanghai, Hubei, Jiangsu, Guizhou, Hunan, Fujian, Chongqing, Sichuan, Jiangxi, Guangdong, Guangxi, Zhejiang), where the onset months of MP epidemic typically begins in July. This zone experiences an epidemic duration of approximately 6.7 months (IQR 6.5–6.9), with two significant peaks in July–August and November. Transmission Zone 2 encompasses Hainan, Neimongol, Beijing, Ningxia, Xinjiang, Yunnan, and Gansu, with an onset months ranging from August to November. Here, the epidemic duration averages 5.6 months (IQR 5.4–5.7), with a single peak in January. Transmission Zone 3 includes nine provinces (Heilongjiang, Jilin, Liaoning, Shaanxi, Henan, Shanxi, Hebei, Tianjin, and Shandong) where the onset month ranges occurs between August and October, leading to an epidemic duration of 5.5 months (IQR 4.9–5.6), peaking in November (Figure 1(B,C)).

### Age- and sex- specific

**Figure 2.**
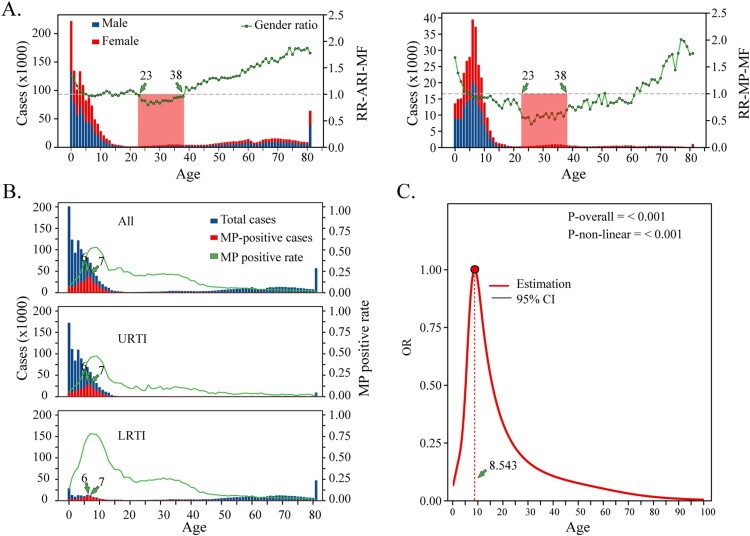
Age-sex specific risk analysis of MP Infections. (a and b) Comparative analysis of ARI (a) or MP infection (b) risk between males and females. The *x*-axis represents age. The red bars denote the number of female cases, while the blue bars denote male cases. The green line represents the risk ratio of ARI (a) or MP (b) infection between males and females (RR-ARI-MF or RR-MP-MF). Values greater than 1 indicates higher infection risk in males, whereas less than 1 indicates higher risk in females. The red boxes mark the age range corresponding to the peak fertility years in females. (c): Three graphs illustrating positivity rates and case numbers for total cases, URTI cases, and LRTI cases. The *x*-axis represents age. The blue bars represent all ARIs case numbers, the red bars represent MP-positive case numbers, and the green line represents MP positivity rate. (d) Risk assessment of MP infections across different age groups. The *x*-axis represents different age groups, the *y*-axis represents the OR values, the red line represents the OR values for different age groups.

From January 2022 to May 2024, among 1,668,960 ARI cases nationwide, the median age of ARI cases was 6 years (IQR 2–50). Newborns aged [0–1) years had the highest numbers of ARI case, with case numbers declining with age, except for a small peak among those aged [50–80) years (Supplemental Table S7). Males accounted for 58.51% (976,485 cases) and females for 41.49% (692,475 cases), with a normalized male-to-female ARI ratio of 1.35. Males had a significantly higher numbers of ARI case than females (*χ*^2 ^= 5273.33, *p* < 0.0001). This finding aligns with the GBD study on sex-specific trends in LRTIs [44]. The risk ratio of ARI between males and females (RR-ARI-MF) was high in newborns aged [0–1) years at 1.48, decreasing rapidly after birth to approach 1 by age 3. In the [23–38) age group, RR-ARI-MF was below 1 (mean 0.889, SD 0.013), indicating a higher risk in females, but this ratio rose again in those aged 39 and older (Figure 2(A) and Supplemental Table S7).

In the total ARI cases, there were 313,799 MP-positive cases, with a positivity rate of 18.80%. The highest number of MP-positive cases were among 6-year-old children, followed by 7-year-olds, with significantly higher positive case numbers compared to other ages. Regarding MP-positivity rates, children aged 7, 8, and 9 years consistently exhibited the highest MP positivity rates among all cases, URTIs cases, and LRTIs cases (Figure 2(B) and Supplemental Table S8). RCS analysis identified 8.543 years as the peak age for MP infection risk, with the risk increasing up to this age and gradually declining thereafter (Figure 2(C)).

Among MP-positive cases, males represented 52.75% (165,552 cases) and females 47.25% (148,247 cases), with a normalized male-to-female MP-positive ratio of 1.07. This indicates that the risk of MP infection is comparable between males and females in the general population. However, there is significant heterogeneity across different age groups. The risk ratio of MP infection between males and females (RR-MP-MF) followed a similar trend to RR-ARI-MF, peaking at 1.67 in newborns aged [0–1) years and declining rapidly post-birth. In the [6–61) age group, RR-MP-MF was less than 1 (mean 0.772, SD 0.019), with the lowest ratio also observed in the [23–38) age group (mean 0.578, SD 0.016). Among those aged 60 and older, RR-MP-MF again exceeded 1, increasing with age (Figure 2(A) and Supplemental Table S7).

The age-sex specific for 15 other respiratory pathogens were also analyzed (Supplemental Table S9 and Figure S4). Male newborns aged [0–1) years are at a consistently higher risk of infection compared to female newborns for all examined respiratory pathogens. This suggests that the elevated infection risk in male newborns may be related to host-specific factors rather than the pathogens themselves, for example differences in immune system function and/or hormone levels, among other factors, between male and female newborns.

Interestingly, across pediatric respiratory pathogens (including RSV, HIN, HMPV, IAV, IBV, MCAT, SPN, SA, HRV, BP, HAdV, HPIV, and MP), females aged [23–38) years have a higher infection risk compared to males. Data from the National Bureau of Statistics of China shows that the ages 23–38 correspond to the peak childbearing years for women (Supplemental Table S10), so perhaps this higher infection risk in females aged [23–38) years reflects increased exposure as a result of relatively greater extent of caregiving work compared to males.

### Macrolide resistance

**Figure 3.**
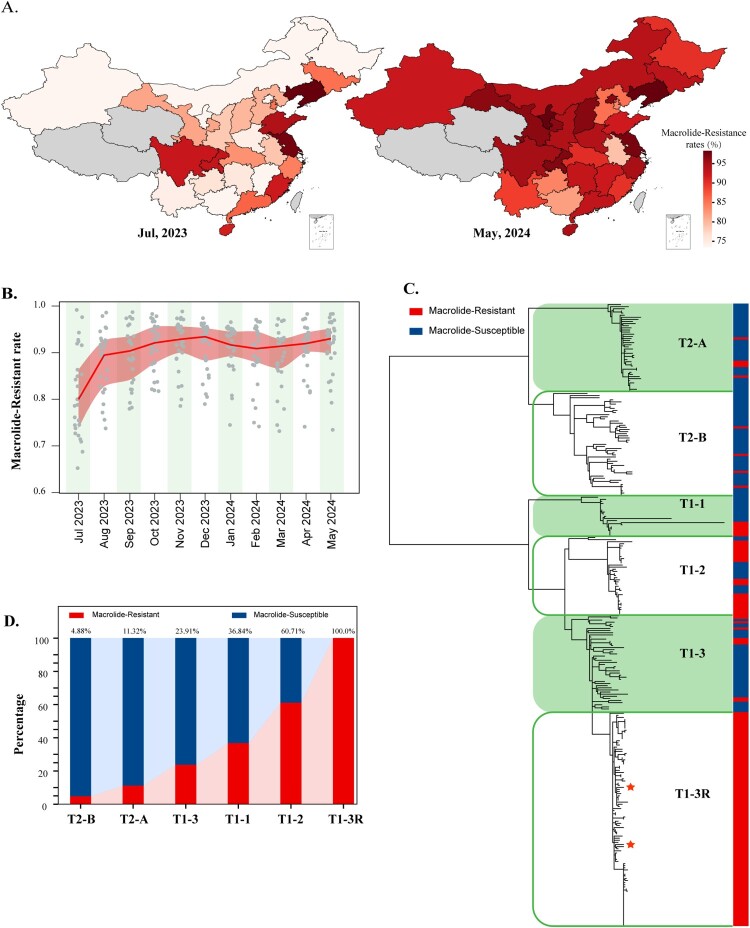
Correlation between changes in Macrolide resistance rates and major epidemic clades of MP. (a) Proportion of MRMP in different provinces across the country in July 2023 and May 2024. (b) Trend chart of MRMP proportions in 29 provinces nationwide from July 2023 to May 2024. The green line represents the median resistance rate trend across 29 provinces for different months, with the upper edge of the green shaded area representing the 75th and 25th percentile of resistance rates. (c) Phylogenetic tree of MP. The two genomes marked with asterisks are strains isolated in this study. The branch information and resistance status of the genomes are annotated on the right side of the phylogenetic tree, with red indicating resistance and blue indicating susceptibility. (d) Comparison of macrolide resistance rates in different clades of MP.

Nationwide, the median proportion of MRMP in July 2023 across the 29 provinces was 80.00% (IQR 73.79%–86.35%). Seven provinces reported MRMP proportions exceeding 90%, with two provinces reporting proportions over 95% (Figure 3(A) and Supplemental Table S11). By May 2024, the median MRMP proportion had increased to 93.02% (IQR 89.81%–95.20%), with 20 provinces exceeding 90% and eight provinces exceeding 95% (Figure 3(A)). Previous studies have shown that in the Western Pacific region, the proportion of MRMP increased from 71.2% in 2011 to 76.5% in 2019, marking a 5.3% increase over a 9-year period [45]. The median growth rate of macrolide resistance from July 2023 to May 2024 was 10.21% (IQR 3.05%–25.43%) in China (Figure 3(B)),which is concerning.

Phylogenetic analysis categorized 433 MP genomes into six clades. The T1-3R clade, which showed a 100% MRMP proportion, was particularly prominent. Among the genomes, 19 belonged to T1-1 (36.84% MRMP), 56 to T1-2 (60.71% MRMP), 91 to T1-3 (24.18% MRMP), 132 to T1-3R (100% MRMP), 53 to T2-A (11.32% MRMP), and 82 to T2-B (4.88% MRMP) (Figure 3(C,D) and Supplemental Table S12). Two strains isolated in this study were part of the T1-3R resistant clade (Supplemental Figure S5 and S6). In Wuhan, the proportion of MP-positive samples belonging to the T1-3R clade increased from 79.05% in August 2023 to 86.36% in May 2024, reflecting a 7.31% increase in this highly resistant clade over the study period (Supplemental Table S12). The recent rapid rise in resistance rates in China is apparently being driven by the proliferation of the T1-3R clade, which exhibits 100% resistance prevalence and is rapidly expanding.

## Discussion

This study presents an extensive epidemiological analysis of MP using data from over 1.6 million ARI cases across China. The large dataset allowed for a detailed examination of MP at the provincial level and provided insights into demographic characteristics at each age, rather than simply focusing on broad age groups. This comprehensive approach enabled us to identify distinct seasonal patterns of MP across China, leading to the classification of three transmission zones Demographic analysis unveils the varying risk of MP infection among different populations, providing valuable insights for the prevention and control of high-risk groups. Additionally, the analysis of the nationwide MP resistance rate and the factors driving its rapid increase provides data support for the control of MP resistance.

This study identified three geographically distinct MP transmission zones in China. Provinces within each zone displayed synchronized seasonal epidemic patterns, supporting the implementation of zone-specific prevention strategies (e.g. coordinated surveillance schedules and resource distribution) to improve outbreak preparedness. Intriguingly, Yunnan and Hainan—geographically southern provinces – were clustered with northern provinces in Transmission Zone II. These regions serve as major winter tourism destinations for northern residents, including a notable influx of pediatric populations. Such seasonal migration patterns may perturb local transmission dynamics, indicating that human mobility acts synergistically with climatic drivers to shape regional heterogeneity in MP seasonality.

In 2023, China faced a severe outbreak of respiratory infections among children [20,46–50]. Previous studies suggest that extended NPIs reduced exposure to pathogens, creating an immunity gap and increasing susceptibility [51,52]. Furthermore, China’s 2016 relaxation of the one-child policy led to 2 million additional births in 2016 and 2017. By 2023, these children were 6–7 years old, an age group at high risk for respiratory infections. Our study found a significant increase in MP-positive cases among 6- and 7-year-olds, indicating that the post-COVID-19 relaxation of NPIs in 2023 and the comprehensive relaxation of the one-child policy in 2016 may have contributed to the severe outbreak of respiratory infections among children.

This study found that women aged [23–38) have a higher risk of infection, which may be related to their role in caring for infectied children. However, we cannot dismiss the possibility of sampling bias, as over-testing in this age group – aimed at protecting pregnant women – could have inflated the number of cases [53]. Since ARI was the criterion for case selection in present study, and women aged [23–38) showed a higher MP positivity rate compared to other adult women (Supplemental Table S7), it suggests that the increased infection risk is more likely due to caregiving responsibilities rather than over-testing.

The rising prevalence of MRMP observed in this study aligns with other studies [6,11]. Notably, China's revised 2023 National Guidelines for Pediatric MP Management (National Health Commission) now recommend doxycycline as an alternative therapeutic option for children, demonstrating therapeutic adaptation to increasing resistance rates. This highlights an urgent public health priority: nationwide implementation of standardized antimicrobial stewardship initiatives to curtail the selection and dissemination of resistant strains.

Although our analysis of T1-3R clade prevalence was based on data from a single center, combined with other studies, it is evident that T1-3R is also a predominant clade in other cities. For example, Li et al. [46] mentioned the EC1 clade prevalent in Suzhou and Shenzhen in 2023, which phylogenetically corresponds to T1-3R clade despite using different nomenclature [54]. Additionally, Chen et al. [18] identified ST3 as the predominant MLST type in most regions of China in 2023, and all strains within the T1-3R clade belong to the ST3 type. This indicates the widespread prevalence of the T1-3R clade in China [18]. Given the increasing prevalence of resistant subgroups and resistance rates for MP in China, it seems evidewnt that it would be useful to include testing for the 23 s rRNA A2063G mutation in clinical practice to guide clinical management.

This study has several limitations. The lack of multi-year data prevents validation of seasonal patterns, as our analysis is based on data from April 2023 to March 2024. Additionally, the number of cases per province reflects only the included cases, not the overall severity of the epidemic, limiting our ability to compare epidemic severity across provinces. Lastly, the positivity rate refers to MP positivity among ARIs, not the general population, and the resistance rate is specific to inpatients, which may be slightly higher than in outpatients. This study exclusively enrolled patients with laboratory-confirmed MP infection through tNGS. However, cases diagnosed via alternative methods (e.g. RT-PCR, serological antibody [IgG/IgM] testing) were excluded, which may limit the epidemiological representativeness of the reported MP incidence. Furthermore, the clinical indications for tNGS application (typically severe or complex presentations) introduce inherent case selection bias, potentially overestimating MP detection rates compared to populations screened with conventional diagnostic protocols. Despite these limitations, our study provides critical insights into the epidemiological characteristics of MP in China, offering valuable guidance for prevention and control strategies, clinical treatment, and protection of high-risk populations. The novel approach of leveraging tNGS and mNGS data in epidemiological research opens new avenues for future studies and serves as a significant resource for understanding the epidemiological characteristics of other respiratory pathogens.

## Supplementary Material

Supplement_revised-clean.docx

## Data sharing

All relevant data supporting the findings are provided in the supplemental material. The in-house script used for analysis is available on GitHub (https://github.com/bioyuan/Analysis-of-Mycoplasma-pneumoniae). De-identified participant data will be shared in accordance with Chinese law and will be available post-publication. Data requests should be directed to Jianguo Xu (xujianguo@icdc.cn). Upon approval and receipt of a signed data access agreement, the requested data will be provided.
