# Supplementary material for: Characterizing the epidemiology of Mycoplasma pneumoniae infections in China in 2022–2024: a nationwide cross-sectional study of over 1.6 million cases
Source: Emerg Microbes Infect. 2025 Mar 27;14(1):2482703. doi: 10.1080/22221751.2025.2482703 (PMC11980206; doi:10.1080/22221751.2025.2482703)
Supplement: Supplement_revised-clean.docx [file TEMI_A_2482703_SM1085.docx]

**Supplemental Material**

**Characterising the epidemiology of *Mycoplasma pneumoniae* infections in China in 2022–2024: a nationwide cross-sectional study of over 1.6 million cases**

Yamin Sun ^1, 6, 7, #^,Pei Li^2, #^, Ronghua Jin ^1, 6, 7, #^, Yaoming Liang ^2, #^, Jiale Yuan ^3, #^, Zhongxin Lu ^5^, Junrong Liang ^4^, Yingmiao Zhang ^5^, Hongyu Ren ^4^, Yuanyuan Zhang ^1, 6, 7^, Jianchun Chen ^2^, Yun Huang ^2^, Chuixu Lin ^2^, Yinghua Li ^2^, Jianfeng Zhou ^2^, Xi Wang ^1, 6, 7^, You Li ^8^, Senzhong Huang ^9^, Jianguo Xu ^4, *^,and Tian Qin ^4, *^

**Author affiliation:**

1 National Key Laboratory of Intelligent Tracking and Forecasting for infectious Diseases, Beijing Ditan Hospital, Capital Medical University, Beijing, China; 2 KingMed Diagnostics, Guangzhou, Guangdong, China; 3 National Key Laboratory of Intelligent Tracking and Forecasting for Infectious Diseases, TEDA Institute of Biological Sciences and Biotechnology, Nankai University, Tianjin, China; 4 National Key Laboratory of Intelligent Tracking and Forecasting for Infectious Diseases, National Institute for Communicable Disease Control and Prevention, Chinese Center for Disease Control and Prevention, Beijing, China; 5 The Central Hospital of Wuhan, Tongji Medical College, Huazhong University of Science and Technology, Wuhan, China; 6 Beijing Institute of Infectious Diseases, Beijing, China; 7 National Center for Infectious Diseases, Beijing Ditan Hospital, Capital Medical University, Beijing, China; 8 Department of Epidemiology, National Vaccine Innovation Platform, School of Public Health, Nanjing Medical University, Nanjing, China; 9 National Key Laboratory of Intelligent Tracking and Forecasting for Infectious Diseases, School of Statistics and Data Science, Nankai University.

* **Corresponding authors:** Prof Tian Qin, National Key Laboratory of Intelligent Tracking and Forecasting for Infectious Diseases, National Institute for Communicable Disease Control and Prevention, Chinese Center for Disease Control and Prevention, 155^#^ Changbai Road, Changping, Beijing 102206, China, qintian@icdc.cn ; Prof Jianguo Xu, National Key Laboratory of Intelligent Tracking and Forecasting for Infectious Diseases, National Institute for Communicable Disease Control and Prevention, Chinese Center for Disease Control and Prevention, 155^#^ Changbai Road, Changping, Beijing 102206, China, xujianguo@icdc.cn.

# These authors contributed equally to this work.

1. **tNGS Data Generation and Analysis**

**1.1 Material Processing and Nucleic Acid Extraction from Nasopharyngeal/Oropharyngeal Swabs**

Swab samples were collected in reference to the established standard procedures. A volume of 1.3 mL from the swab sample was aliquoted, to which 13 μL of an exogenous internal reference was added. The mixture was subjected to vortexing for homogenous mixing followed by centrifugation at 12,000 rpm for 5 minutes. The supernatant was carefully discarded, the pellet was resuspended in 250 μL by pipetting up and down to ensure thorough mixing. Nucleic acids were then extracted using the MagPure Pathogen RNA/DNA Extraction Kit (Guangzhou Magen Biotechnology Co.,Ltd.), following the manufacturer's protocol. The extracted nucleic acids were used for subsequent library construction.

**1.2 Material Processing and Nucleic Acid Extraction from Sputum/Alveolar Lavage Fluid**

Sputum or alveolar lavage samples were collected in reference to the established standard procedures. For viscous alveolar lavage fluid or sputum samples, an additional liquefaction step of adding DTT is required during which an equal volume of 0.1 M dithiothreitol (DTT) liquefaction agent was added to the collection tube, vortex for thoroughly mixed, and sit at room temperature for 3-5 min to ensure complete liquefaction. Non-viscous alveolar lavage fluid samples do not require a 0.1 M DTT treatment step. Subsample 1.3 mL of the liquefied mix, add 13 μL of exogenous endogenous reference, votex to ensure thorough mix, and centrifuge at 12000 rpm for 5 min.

The supernatant was discarded, and the residual sample volume was adjusted to 500 μL by pipetting. This aliquot was transfer into the bead mill tube provided in the extraction kit, to which add 50 μL of SDS was added, and them subjected to the wall-breaking apparatus (4700 rpm, oscillate for 45 s, with an intermittent pause of 20s, and 2 intervals, a total of 3 oscillations of 135 s) for mechanical lysis. Following mechanical lysis, the samples were centrifuged at 12,000 rpm for 5 minutes, and 250 μL of the supernatant was used for nucleic acid extraction. The extraction was performed using the MagPure Pathogen RNA/DNA Extraction Kit（Guangzhou Magen Biotechnology Co.,Ltd.）, following the manufacturer's protocol. The extracted nucleic acids were quantified using Equalbit DNA HS Assay Kit (Vazyme Biotech, Nanjing, Jiangsu, China) with a Invitrogen™ Qubit™ 3.0/4.0 (Thermo Fisher Scientific, Waltham, MA, USA) , and the input nucleic acids did not exceed 100ng for library construction.

**1.3 Library Preparation and Sequencing**

Library preparation were performed using the RP100^TM^ Respiratory Pathogen Microorganisms Multiplex Testing Kit (KingCreate Biotechnology Co.,Ltd., Guangzhou, China) for Nasopharyngeal/Oropharyngeal Swabs, Sputum and Alveolar Lavage Fluid and using URP50^TM^ Respiratory Pathogen Microorganisms Multiplex Testing Kit (KingCreate, Guangzhou, Guangdong, China) for Nasopharyngeal/Oropharyngeal Swabs only . cDNA was synthesised by reverse transcription of the extracted nucleic acids, followed by steps such as target region enrichment PCR, PCR product purification, adapter ligation and library purification to complete library construction. Nuclease-free water (Invitrogen, Waltham, MA, USA) was used as NTC (non-template control) to detect contamination.

Generated libraries were quantified using Equalbit DNA HS Assay Kit (Vazyme Biotech, Nanjing, Jiangsu, China) with a Invitrogen™ Qubit™ 3.0/4.0 (Thermo Fisher Scientific, Waltham, MA, USA) . Fluorometer to ensure all samples were with library density ≥ 0.5 ng/μL or else the library should be subjected to re-construction. The constructed libraries were pooled to homogeneous mass. The size of the library fragments was determined by a automated nucleic acid protein analyzer (Qsep100) using Standard Cartridge Kit (S2). The size of the library fragments should be of from 250 to 350 bp. Qualified pooled library was diluted and denatured, 500 μL of which was subjected to KM MiniSeq Dx-CN Platform (KingCreate, Guangzhou, Guangdong, China) for sequencing.

**1.4 Bioinformatics**

Generated sequencing raw read data underwent quality control procedure. The fastp v0.20.1 was employed for adapter trimming and quality trimming using default parameters followed by mapping to the reference using Bowtie2 v2.4.1 in ‘very-sensitive’ mode. The reference sequences used for read mapping was a database curated from various sources including the GenBank, RefSeq, and NT databases from NCBI (https://www.ncbi.nlm.nih.gov). To identify positive signals for specific pathogens, the number of mapped reads were counted and normalized to reads per 100,000 (RPhK). Cases with specific RPhKs were considered as positive for each sample. If a specific species or high-level taxonomy unit was identified in a sample with RPhK value ≥ 10, this species unit was regarded as “present” in this sample, otherwise, it was reported as “absent”.

1. **Statistical and Model Analysis**

**2.1 Positive Rate**

We calculated the each provincemonthly positive rate (PO). PO is defined as:

$$\begin{aligned} \boldsymbol{PO}_{\boldsymbol{ij}}\boldsymbol{=}\frac{\boldsymbol{P}_{\boldsymbol{ij}}}{\boldsymbol{R}_{\boldsymbol{ij}}}\boldsymbol{\times100\%\#}\left( \boldsymbol{1} \right) \end{aligned}$$

where i represents each province, j represents each month, P is the number of MP positive cases, and R is the total number of ARI cases.

**2.2 The normalized number of MP-positive cases**

To avoid the impact of differences in each province ARI case numbers on MP-positive cases, we normalized the number of MP-positive cases by using the number of MP-positive cases per 1000 ARI cases as the standardized figure. The formula is as follows:

$$\boldsymbol{P}_{\boldsymbol{normalized ij}\boldsymbol{=}}\frac{\boldsymbol{P}_{\boldsymbol{ij}}}{\boldsymbol{R}_{\boldsymbol{ij}}}\boldsymbol{\times1000}$$

where i represents each province, j represents each month, P is the number of MP positive cases, and R is the total number of ARI cases.

This formula ensures that the MP-positive case numbers are adjusted relative to the number of ARI cases, facilitating fair comparisons across provinces and months.

**2.3 AAP Value and Epidemic Months Identification**

We identified epidemic months using the Annual Average Percentage (AAP) value from normalized number of MP-posi tive cases across 29 provinces (excluding Qinghai and Tibet due to insufficient data) from April 2023 to March 2024. The AAP is calculated as:

$$\begin{aligned} \boldsymbol{AAP}_{\boldsymbol{ij}}\boldsymbol{=}\frac{\boldsymbol{P}_{\boldsymbol{normalized ij}}}{\sum_{\boldsymbol{12}}^{\boldsymbol{1}} \boldsymbol{P}_{\boldsymbol{normalized ij}}}\boldsymbol{\times100\%\#}\left( \boldsymbol{2} \right) \end{aligned}$$

​

where i represents each province, j represents each month, where $P_{normalized}$ is the number of MP-positive cases after normalization. We sorted the AAP values for each province in descending order and identified epidemic months as those contributing to the top 75% of cumulative AAP. We defined the onset of a season by the beginning month of consecutive epidemic months.

**2.4 Sensitivity analysis of the clustering analysis**

Clustering stability was assessed via Gaussian Mixture Models (GMM) initialized with K-means++ parameters. The expectation-maximization (EM) algorithm was iterated (max 1000 iterations, convergence threshold 1e-6) to optimize model fit. A benchmark GMM was established by aligning its output with the original K-means clusters. Stability was quantified across 10 independent runs (100 random initializations per run, total n=1000 samples). Cluster labels were aligned using the Hungarian algorithm, and invalid or aberrantly clustered samples were excluded. The proportion of runs achieving perfect concordance with the benchmark grouping was calculated to evaluate stability.

.

**2.5 RCS Infection Risk Analysis**

We used whether MP infection occurred as the dependent variable, conducted RCS logistic regression analysis on the relationship between age (independent variable) and the dependent variable, predicted MP infection risks across different ages, and included gender, region, and infection site as covariates. The RCS infection risk prediction and visualization were performed using the rcssci package in R.

1. **Genome/Metagenome Sequence and Phylogenetic analysis**

**3.1 Phylogenetic analysis with Whole Genome Sequence**

The strategy of whole genome sequencing used was a combination of Illumina Novaseq (Illumina Inc., San Diego, CA, USA) and Oxford Nanopore PromethION 2. Illumina paired-end sequencing library was prepared using TruSeq DNA sample prep kits (Illumina Inc., San Diego, CA, USA). The library preparation was carried out using the ligation sequencing kits (Oxford Nanopore Technologies) SQK-LSK112 for sequencing on the R.10.4 flowcells. Long reads were assembled using Flye v. 2.9-b1768 (12, 13) with the ‘-nano-hq’ option for assembling Nanopore reads. The Pilon v1.22 software was used to correct the assembly with Illumina data, and Circulator v1.5.5 software was used to cyclize and adjust the staring site. Completed, circularized genomes were annotated using RAST. We used 294 MP genomes, including two sequenced by us, and aligned them with the reference genome M129-B7 (GenBank CP003913) using snippy (v4.6.0). A maximum likelihood tree was constructed with iqtree (v2.2.0.3) using the GTR+Γ+I nucleotide substitution model. Phylogenetic trees were visualized using FigTree v1.4.4 and iTol software.

**3.2 Metagenome sequence and analysis**

Altogether, 251 MP-PCR positive throat swabs were sequenced on an Illumina HiSeq Novaseq platform (San Diego, CA, USA). The raw sequencing reads were trimmed for quality using fastp (v. 0.22.0). To identify the MP clade of the 251 sequenced samples, we first used Python scripts to extract clade-specific SNP sites of MP. The filtered data were aligned to the reference genome M129-B7 (GenBank CP003913) using BWA (v. 0.7.17-r1188). SAMtools was then used to extract the reads aligned to the reference genome, and BCFtools was used to extract the single nucleotide polymorphism (SNP) information for each sample. The clade of the 251 sequenced samples were identified based on the clade-specific SNP sites of each clade. The resistance of each sample was inferred based on four MP resistance mutation sites (A2063G, A2064G, C2617G, and A2067G in the 23s rRNA gene).

**Table S1. infomation of pathogens and resistance genes detected by tNGS using the RP100^TM^ Kit.**

| **RP100^TM^** | | | | | |
| --- | --- | --- | --- | --- | --- |
| **Viruses(69)** |  |  |  |  |  |
| *BK polyomavirus (Human polyomavirus 1)* | *Human adenovirus 11* | *Human respiratory syncytial virus A (HRSV-A)* | *Cytomegalovirus (CMV)* | *Enterovirus A71* | *Influenza B virus lineage Yamagata* |
| *Human adenovirus 1* | *Human adenovirus 14* | *Human respiratory syncytial virus B (HRSV-B)* | *Human herpesvirus 6* | *Enterovirus B* | *Measles virus* |
| *Human adenovirus 2* | *Human adenovirus 34* | *Human parainfluenza virus 1 (HPIV-1)* | *Human herpesvirus 7* | *Enterovirus C* | *Mumps virus* |
| *Human adenovirus 21* | *Human adenovirus 35* | *Human parainfluenza virus 3 (HPIV-3)* | *Human bocavirus 1* | *Enterovirus D* | *Rhinovirus* |
| *Human adenovirus 5* | *Human herpesvirus 6A* | *Influenza A virus* | *Human bocavirus 2* | *Enterovirus D68* | *Rhinovirus A* |
| *Human adenovirus 55* | *Human herpesvirus 6B* | *Influenza A virus subtype H1N1* | *Human bocavirus 3* | *Human coronavirus 229E* | *Rhinovirus B* |
| *Human adenovirus 57* | *JC polyomavirus (Human polyomavirus 2)* | *Influenza A virus subtype H3N2* | *Human bocavirus 4* | *Human coronavirus HKU1* | *Rhinovirus C* |
| *Human adenovirus 6* | *WU polyomavirus (Human polyomavirus 4)* | *Influenza A virus subtype H5N1* | *Epstein-Barr virus (EBV)* | *Human coronavirus NL63* | *Rubella virus* |
| *Human adenovirus 7* | *Coxsackievirus A5* | *Influenza A virus subtype H7N9* | *Human adenovirus* | *Human coronavirus OC43* | *SARS-CoV-2* |
| *Human adenovirus 3* | *Coxsackievirus A6* | *Influenza B virus* | *Human adenovirus group B* | *Human metapneumovirus* | *Human parainfluenza virus 4 (HPIV-4)* |
| *Human adenovirus 4* | *Coxsackievirus B3* | *Coxsackievirus A10* | *Human adenovirus group C* | *Human parainfluenza virus 2 (HPIV-2)* | *Human adenovirus group D* |
| *Herpes simplex virus 1 (HSV1)* | *Echovirus E18* | *Coxsackievirus A16* | .. | .. | .. |
| **Bacteria(80)** |  |  |  |  |  |
| *Corynebacterium diphtheriae* | *Nocardia cerradoensis (Nocardia transvalensis)* | *Klebsiella aerogenes* | *Mycobacterium tuberculosis complex* | *Burkholderia cepacia complex* | *Haemophilus pertussis* |
| *Mycobacterium asiaticum* | *Nocardia farcinica* | *Klebsiella oxytoca* | *Mycobacterium xenopi* | *Burkholderia mallei* | *Brucella* |
| *Mycobacterium avium* | *Nocardia neocaledoniensis* | *Klebsiella pneumoniae* | *Mycobacterium abscessus* | *Burkholderia pseudomallei* | *Burkholderia cenocepacia* |
| *Mycobacterium avium complex* | *Nocardia otitidiscaviarum* | *Klebsiella variicola* | *Mycobacterium chelonge-abscessus complex* | *Burkholderia contaminans* | *Burkholderia cepacia* |
| *Mycobacterium celatum* | *Nocardia terpenica* | *Legionella* | *Mycobacterium chelonae* | *Burkholderia multivorans* | *Neisseria meningitidis* |
| *Mycobacteriumgordonae* | *Micromonospora parva* | *Legionella bozemanii* | *Mycobacterium fortuitum* | *Elizabethkingia anophelis* | *Pasteurella multocida* |
| *Mycobacterium intracellulare* | *Rhodococcus equi* | *Legionella pneumophila* | *Mycobacterium smegmatis* | *Elizabethkingia meningoseptica* | *Proteus mirabilis* |
| *Mycobacterium kansasii* | *Staphylococcus aureus* | *Legionella longbeachae* | *Nocardia* | *Enterobacter cloacae complex* | *Pseudomonas aeruginosa* |
| *Mycobacterium malmoense* | *Streptococcus agalactiae* | *Legionella mikedaensis* | *Nocardia abscessus* | *Escherichia coli* | *Serratia marcescens* |
| *Mycobacterium nonchromogenicum* | *Streptococcus anginosus group* | *Moraxella catarrhalis* | *Nocardia africana* | *Fusobacterium necrophorum* | *Stenotrophomonas maltophilia* |
| *Mycobacterium scrofulaceum* | *Streptococcus intermedius* | *Acinetobacter baumannii* | *Nocardia asteroides* | *Fusobacterium nucleatum* | *Haemophilus parainfluenzae* |
| *Mycobacterium shimoidei* | *Streptococcus pneumoniae* | *Acinetobacter junii* | *Nocardia brasiliensis* | *Haemophilus influenzae* | *Arcanobacterium pyogenes* |
| *Mycobacterium simiae* | *Streptococcus pyogenes* | *Acinetobacter ursingii* | *Nocardia caviae* | *Haemophilus influenzae biogroup aegyptius* | *Bacteroides fragilis* |
| *Mycobacterium suricattae* | *Calymmatobacterium granulomatis* | .. | .. | .. | .. |
| **Fungi(32)** |  |  |  |  |  |
| *Candida glabrata* | *Fusarium* | *Rhizomucor pusillus* | *Candida pseudotropicalis* | *Mucor racemosus* | *Talaromyces marneffei* |
| *Aspergillus flavus complex* | *Histoplasma capsulatum* | *Rhizopus* | *Candida parapsilosis* | *Candida krusei* | *Trichosporon asahii* |
| *Aspergillus fumigatus* | *Aphanoascus* | *Rhizopus delemar* | *Candida tropicalis* | *Pneumocystis jirovecii* | *Scedosporium boydii* |
| *Aspergillus niger complex* | *Aphanoascus fulvescens* | *Rhizopus microsporus* | *Cryptococcus gattii* | *Rhizomucor* | *Absidia corymbifera* |
| *Aspergillus terreus complex* | *Aphanoascus mephitalis* | *Rhizopus oryzae* | *Cryptococcus neoformans* | *Scedosporium apiospermum* | *Scedosporium* |
| *Candida albicans* | *Pichia guilliermondii* | .. | .. | .. | .. |
| **Other(7)** |  |  |  |  |  |
| *Chlamydophila pneumoniae* | *Mycoplasma pneumoniae* | *Coxiella burnetii* | *Chlamydophila psittaci* | *Ureaplasma parvum* | *Chlamydia trachomatis* |
| *Ureaplasma urealyticum* | .. | .. | .. | .. | .. |
| **Mycoplasma pneumoniae drug resistance detection site*（4）** |  |  |  |  |  |
| 23S rRNA:A2063G | 23S rRNA:A2064G | 23S rRNA:C2617G | 23S rRNA:A2067G | .. | .. |

* indicates that resistance detection started on 2023-07-01.

**Table S2. infomation of pathogens and resistance genes detected by tNGS using the URP50™ Kit.**

| **URP50™** | | | | | |
| --- | --- | --- | --- | --- | --- |
| **Viruse(74)** |  |  |  |  |  |
| *Coxsackievirus A10* | *Epstein-Barr virus* | *Human Adenovirus 30* | *Human Adenovirus E* | *Human Parvovirus B19* | *Measles Virus* |
| *Coxsackievirus A16* | *Herpes Simplex Virus 1* | *Human Adenovirus 31* | *Human Bocavirus 1* | *Human Respiratory Syncytial Virus 1* | *Mumps Virus* |
| *Coxsackievirus A5* | *Herpes Simplex Virus 2* | *Human Adenovirus 34* | *Human Coronavirus 229E* | *Human Respiratory Syncytial Virus 3* | *Rhinovirus* |
| *Coxsackievirus A6* | *Human Adenovirus* | *Human Adenovirus 38* | *Human Coronavirus HKU1* | *Human Respiratory Syncytial Virus A* | *Rhinovirus A* |
| *Cytomegalovirus* | *Human Adenovirus 1* | *Human Adenovirus 4* | *Human Coronavirus NL63* | *Human Respiratory Syncytial Virus B* | *Rhinovirus B* |
| *Echovirus E18* | *Human Adenovirus 12* | *Human Adenovirus 5* | *Human Coronavirus OC43* | *Influenzavirus A* | *Rhinovirus C* |
| *Enterovirus* | *Human Adenovirus 18* | *Human Adenovirus 55* | *Human Herpesvirus 6* | *Influenzavirus A H1N1* | *Rubella Virus* |
| *Enterovirus A* | *Human Adenovirus 2* | *Human Adenovirus 7* | *Human Herpesvirus 6A* | *Influenzavirus A H1N1 (2009)* | *SARS-CoV-2* |
| *Enterovirus A71* | *Human Adenovirus 21* | *Human Adenovirus 8* | *Human Herpesvirus 6B* | *Influenzavirus A H3N2* | *Varicella-zoster virus* |
| *Enterovirus B* | *Human Adenovirus 24* | *Human Adenovirus A* | *Human Herpesvirus 7* | *Influenzavirus A H5N1* | *Influenzavirus C* |
| *Enterovirus C* | *Human Adenovirus 27* | *Human Adenovirus B* | *Human Metapneumovirus* | *Influenzavirus A H7N9* | *Human Parainfluenza Virus 4* |
| *Enterovirus D* | *Human Adenovirus 28* | *Human Adenovirus C* | *Human Parainfluenza Virus 2* | *Influenzavirus B* | *Human Adenovirus D* |
| *Enterovirus D68* | *Human Adenovirus 3* | .. | .. | .. | .. |
| **Bacteria(70)** |  |  |  |  |  |
| *Streptococcus pneumoniae* | *Haemophilus influenzae* | *Acinetobacter baumannii* | *Mycobacterium haemophilum* | *Mycobacterium gordonae* | *Mycobacterium malmoense* |
| *Streptococcus agalactiae* | *Neisseria gonorrhoeae* | *Mycobacterium tuberculosis complex* | *Mycobacterium peregrinum* | *Mycobacterium ulcerans* | *Mycobacterium scrofulaceum* |
| *Anaerococcus haemolyticus* | *Moraxella catarrhalis* | *Mycobacterium avium* | *Mycobacterium lentiflavum* | *Mycobacterium decascendens* | *Mycobacterium persicum* |
| *Streptococcus pyogenes* | *Bordetella pertussis* | *Mycobacterium intracellulare* | *Mycobacterium margaritense* | *Mycobacterium szulgai* | *Mycobacterium asiaticum* |
| *Streptococcus dysgalactiae subsp. dysgalactiae* | *Legionella pneumophila* | *Mycobacterium abscessus subsp. abscessus* | *Mycobacterium cosmeticum* | *Mycobacterium intermedium* | *Mycobacterium smegmatis* |
| *Streptococcus intermedius* | *Fusobacterium necrophorum* | *Mycobacterium abscessus subsp. massiliense* | *Mycobacterium massiliense* | *Mycobacterium simiae* | *Mycobacterium wound complex* |
| *Staphylococcus aureus* | *Yersinia enterocolitica* | *Mycobacterium abscessus subsp. bolletii* | *Mycobacterium bovis* | *Mycobacterium haemophilum* | *Mycobacterium europaeum* |
| *Corynebacterium diphtheriae* | *Haemophilus influenzae type b* | *Mycobacterium chelonae* | *Mycobacterium phlei* | *Mycobacterium gastri* | *Mycobacterium flavescens* |
| *Listeria monocytogenes* | *Pseudomonas aeruginosa* | *Mycobacterium kansasii* | *Mycobacterium neoaurum* | *Mycobacterium chimaera* | *Mycobacterium shinae* |
| *Streptococcus constellatus group* | *Serratia marcescens* | *Mycobacterium liflandii* | *Mycobacterium nonchromogenicum* | *Mycobacterium lindaoense* | *Nontuberculous mycobacterial* |
| *Helicobacter pylori* | *Stenotrophomonas maltophilia* | *Mycobacterium fortuitum* | *Mycobacterium terrae complex* | *Mycobacterium paraintracellulare* | *Mycobacterium colombiense* |
| *Neisseria meningitidis* | *Klebsiella pneumoniae* | *Mycobacterium avium subsp. hominissuis* | *Mycobacterium minor* | .. | .. |
| **Other(8)** |  |  |  |  |  |
| *Mycoplasma pneumoniae* | *Chlamydophila psittaci* | *Ureaplasma urealyticum* | *Mycoplasma hominis* | *Ureaplasma parvum* | *Mycoplasma genitalium* |
| *Chlamydophila pneumoniae* | *Chlamydia trachomatis* | .. | .. | .. | .. |
| **Mycoplasma pneumoniae drug resistance detection site*（4）** |  |  |  |  |  |
| 23S rRNA:A2063G | 23S rRNA:A2064G | 23S rRNA:C2617G | 23S rRNA:A2067G | .. | .. |

**Table S3. The monthly MP positivity rates for each province from April 2023 to March 2024.**

| **Province** | **Apr-23** | **May-23** | **Jun-23** | **Jul-23** | **Aug-23** | **Sep-23** | **Oct-23** | **Nov-23** | **Dec-23** | **Jan-24** | **Feb-24** | **Mar-24** |
| --- | --- | --- | --- | --- | --- | --- | --- | --- | --- | --- | --- | --- |
| Shanghai | 0.03846 | 0.10284 | 0.14119 | 0.27283 | 0.31000 | 0.35900 | 0.37900 | 0.39600 | 0.31600 | 0.25000 | 0.18000 | 0.10400 |
| Yunnan | 0.01500 | 0.01200 | 0.02000 | 0.03400 | 0.04200 | 0.03800 | 0.05400 | 0.09400 | 0.09800 | 0.12200 | 0.12300 | 0.07700 |
| Neimongol | 0.00791 | 0.00529 | 0.03202 | 0.06653 | 0.13178 | 0.23097 | 0.30728 | 0.28370 | 0.27526 | 0.40602 | 0.29949 | 0.24966 |
| Beijing | 0.01163 | 0.02597 | 0.04128 | 0.03684 | 0.06485 | 0.14671 | 0.18724 | 0.25500 | 0.27200 | 0.34700 | 0.29014 | 0.17385 |
| Jilin | 0.00305 | 0.00647 | 0.02459 | 0.05799 | 0.10621 | 0.26500 | 0.24147 | 0.34196 | 0.28311 | 0.27300 | 0.15100 | 0.09500 |
| Sichuan | 0.03700 | 0.05700 | 0.08400 | 0.20200 | 0.22600 | 0.17400 | 0.26100 | 0.31300 | 0.29600 | 0.24850 | 0.17700 | 0.10400 |
| Tianjin | 0.01000 | 0.01400 | 0.02300 | 0.08200 | 0.19000 | 0.30200 | 0.47300 | 0.26178 | 0.19956 | 0.18868 | 0.18519 | 0.09586 |
| Ningxia | 0.00725 | 0.03015 | 0.05941 | 0.08734 | 0.16571 | 0.22500 | 0.33099 | 0.38889 | 0.34607 | 0.46869 | 0.42857 | 0.35366 |
| Anhui | 0.18011 | 0.33209 | 0.40900 | 0.51200 | 0.48600 | 0.41200 | 0.48400 | 0.40600 | 0.28700 | 0.20100 | 0.16600 | 0.13400 |
| Shandong | 0.00700 | 0.01200 | 0.02300 | 0.07000 | 0.09500 | 0.16100 | 0.28900 | 0.30800 | 0.21200 | 0.18100 | 0.20700 | 0.16500 |
| Shanxi | 0.00664 | 0.00382 | 0.04261 | 0.07042 | 0.13740 | 0.12023 | 0.17456 | 0.28595 | 0.26275 | 0.19500 | 0.14920 | 0.13727 |
| Guangdong | 0.03700 | 0.06600 | 0.14400 | 0.24800 | 0.28200 | 0.23600 | 0.31900 | 0.35100 | 0.29500 | 0.24800 | 0.21200 | 0.13200 |
| Guangxi | 0.03500 | 0.07500 | 0.13400 | 0.20200 | 0.22000 | 0.15800 | 0.20600 | 0.22400 | 0.19800 | 0.18200 | 0.17600 | 0.11900 |
| Xinjiang | 0.02632 | 0.02326 | 0.01600 | 0.12000 | 0.18552 | 0.23750 | 0.20937 | 0.27677 | 0.20793 | 0.38100 | 0.37998 | 0.36875 |
| Jiangsu | 0.08700 | 0.15800 | 0.23000 | 0.38250 | 0.43800 | 0.41600 | 0.50800 | 0.48200 | 0.35500 | 0.24400 | 0.19300 | 0.16250 |
| Jiangxi | 0.01228 | 0.03300 | 0.09500 | 0.20400 | 0.19939 | 0.17400 | 0.18000 | 0.22400 | 0.16900 | 0.15000 | 0.12500 | 0.11400 |
| Hebei | 0.00585 | 0.02455 | 0.03200 | 0.09500 | 0.15473 | 0.18969 | 0.26821 | 0.39946 | 0.27846 | 0.18300 | 0.12908 | 0.09266 |
| Henan | 0.01400 | 0.02400 | 0.04700 | 0.10600 | 0.17450 | 0.20100 | 0.31900 | 0.34700 | 0.22400 | 0.18500 | 0.20900 | 0.15600 |
| Zhejiang | 0.07968 | 0.16300 | 0.22600 | 0.39000 | 0.40100 | 0.35600 | 0.44200 | 0.46300 | 0.38400 | 0.31900 | 0.24800 | 0.21800 |
| Hainan | 0.02800 | 0.02900 | 0.05700 | 0.15400 | 0.19000 | 0.15500 | 0.21000 | 0.36900 | 0.40600 | 0.37600 | 0.29800 | 0.18900 |
| Hubei | 0.04000 | 0.10800 | 0.21000 | 0.38700 | 0.43200 | 0.37400 | 0.48300 | 0.51000 | 0.40400 | 0.28800 | 0.21700 | 0.17300 |
| Hunan | 0.03000 | 0.05400 | 0.10300 | 0.19900 | 0.23000 | 0.18300 | 0.27000 | 0.32000 | 0.30600 | 0.21600 | 0.15700 | 0.12600 |
| Gansu | 0.00962 | 0.01351 | 0.10390 | 0.10804 | 0.13430 | 0.10049 | 0.07492 | 0.14927 | 0.08600 | 0.26480 | 0.26728 | 0.23197 |
| Fujian | 0.07364 | 0.17834 | 0.27100 | 0.44600 | 0.47700 | 0.40600 | 0.40700 | 0.44900 | 0.36400 | 0.39800 | 0.32000 | 0.22300 |
| Guizhou | 0.03514 | 0.02000 | 0.04300 | 0.11500 | 0.14500 | 0.12600 | 0.13500 | 0.16300 | 0.14100 | 0.12100 | 0.08500 | 0.05500 |
| Liaoning | 0.00273 | 0.00900 | 0.01900 | 0.07100 | 0.19600 | 0.31500 | 0.45550 | 0.44000 | 0.31400 | 0.21200 | 0.09500 | 0.06600 |
| Chongqing | 0.01600 | 0.04100 | 0.08400 | 0.25700 | 0.24200 | 0.13900 | 0.23600 | 0.31850 | 0.34000 | 0.26700 | 0.18600 | 0.14500 |
| Shaanxi | 0.01000 | 0.03000 | 0.06200 | 0.12700 | 0.21600 | 0.22600 | 0.42900 | 0.48500 | 0.26900 | 0.14100 | 0.08600 | 0.06200 |
| Heilongjiang | 0.00184 | 0.00298 | 0.00900 | 0.03900 | 0.07300 | 0.13900 | 0.31200 | 0.33500 | 0.32650 | 0.32600 | 0.22300 | 0.12400 |

**Table S4. The monthly AAP for each province from April 2023 to March 2024**

| **Province** | **Apr-23** | **May-23** | **Jun-23** | **Jul-23** | **Aug-23** | **Sep-23** | **Oct-23** | **Nov-23** | **Dec-23** | **Jan-24** | **Feb-24** | **Mar-24** |
| --- | --- | --- | --- | --- | --- | --- | --- | --- | --- | --- | --- | --- |
| Shanghai | 0.0135 | 0.03609 | 0.04955 | 0.09575 | 0.1088 | 0.126 | 0.13301 | 0.13898 | 0.1109 | 0.08774 | 0.06317 | 0.0365 |
| Yunnan | 0.02058 | 0.01646 | 0.02743 | 0.04664 | 0.05761 | 0.05213 | 0.07407 | 0.12894 | 0.13443 | 0.16735 | 0.16872 | 0.10562 |
| Neimongol | 0.00344 | 0.0023 | 0.01395 | 0.02898 | 0.0574 | 0.1006 | 0.13384 | 0.12357 | 0.11989 | 0.17684 | 0.13045 | 0.10874 |
| Beijing | 0.00628 | 0.01402 | 0.02229 | 0.01989 | 0.035 | 0.07919 | 0.10108 | 0.13765 | 0.14683 | 0.18731 | 0.15662 | 0.09385 |
| Jilin | 0.00165 | 0.0035 | 0.0133 | 0.03137 | 0.05744 | 0.14333 | 0.13061 | 0.18496 | 0.15313 | 0.14766 | 0.08167 | 0.05138 |
| Sichuan | 0.01698 | 0.02615 | 0.03854 | 0.09268 | 0.10369 | 0.07983 | 0.11975 | 0.14361 | 0.13581 | 0.11402 | 0.08121 | 0.04772 |
| Tianjin | 0.00494 | 0.00691 | 0.01136 | 0.04049 | 0.09382 | 0.14913 | 0.23357 | 0.12927 | 0.09855 | 0.09317 | 0.09145 | 0.04734 |
| Ningxia | 0.00251 | 0.01043 | 0.02054 | 0.0302 | 0.05731 | 0.07781 | 0.11446 | 0.13448 | 0.11968 | 0.16208 | 0.14821 | 0.1223 |
| Anhui | 0.04492 | 0.08283 | 0.10202 | 0.12771 | 0.12122 | 0.10276 | 0.12072 | 0.10127 | 0.07159 | 0.05013 | 0.0414 | 0.03342 |
| Shandong | 0.00405 | 0.00694 | 0.01329 | 0.04046 | 0.05491 | 0.09306 | 0.16705 | 0.17803 | 0.12254 | 0.10462 | 0.11965 | 0.09538 |
| Shanxi | 0.00419 | 0.00241 | 0.02687 | 0.04441 | 0.08664 | 0.07582 | 0.11007 | 0.18031 | 0.16568 | 0.12296 | 0.09408 | 0.08656 |
| Guangdong | 0.0144 | 0.02568 | 0.05603 | 0.0965 | 0.10973 | 0.09183 | 0.12412 | 0.13658 | 0.11479 | 0.0965 | 0.08249 | 0.05136 |
| Guangxi | 0.01814 | 0.03888 | 0.06947 | 0.10472 | 0.11405 | 0.08191 | 0.10679 | 0.11612 | 0.10264 | 0.09435 | 0.09124 | 0.06169 |
| Xinjiang | 0.01082 | 0.00956 | 0.00658 | 0.04933 | 0.07627 | 0.09764 | 0.08607 | 0.11379 | 0.08548 | 0.15664 | 0.15622 | 0.1516 |
| Jiangsu | 0.0238 | 0.04322 | 0.06291 | 0.10462 | 0.1198 | 0.11379 | 0.13895 | 0.13184 | 0.0971 | 0.06674 | 0.05279 | 0.04445 |
| Jiangxi | 0.00731 | 0.01965 | 0.05656 | 0.12145 | 0.11871 | 0.10359 | 0.10716 | 0.13336 | 0.10062 | 0.0893 | 0.07442 | 0.06787 |
| Hebei | 0.00316 | 0.01325 | 0.01727 | 0.05128 | 0.08352 | 0.10239 | 0.14477 | 0.21561 | 0.1503 | 0.09878 | 0.06967 | 0.05002 |
| Henan | 0.00698 | 0.01196 | 0.02342 | 0.05283 | 0.08697 | 0.10017 | 0.15898 | 0.17294 | 0.11164 | 0.0922 | 0.10416 | 0.07775 |
| Zhejiang | 0.02159 | 0.04418 | 0.06125 | 0.1057 | 0.10868 | 0.09649 | 0.11979 | 0.12549 | 0.10407 | 0.08646 | 0.06721 | 0.05908 |
| Hainan | 0.01138 | 0.01178 | 0.02316 | 0.06258 | 0.0772 | 0.06298 | 0.08533 | 0.14994 | 0.16497 | 0.15278 | 0.12109 | 0.0768 |
| Hubei | 0.01103 | 0.02978 | 0.05792 | 0.10673 | 0.11914 | 0.10314 | 0.1332 | 0.14065 | 0.11142 | 0.07943 | 0.05985 | 0.04771 |
| Hunan | 0.01367 | 0.02461 | 0.04695 | 0.0907 | 0.10483 | 0.08341 | 0.12306 | 0.14585 | 0.13947 | 0.09845 | 0.07156 | 0.05743 |
| Gansu | 0.00623 | 0.00875 | 0.06729 | 0.06997 | 0.08698 | 0.06508 | 0.04852 | 0.09667 | 0.0557 | 0.17149 | 0.1731 | 0.15023 |
| Fujian | 0.01835 | 0.04444 | 0.06753 | 0.11114 | 0.11886 | 0.10117 | 0.10142 | 0.11189 | 0.09071 | 0.09918 | 0.07974 | 0.05557 |
| Guizhou | 0.02968 | 0.01689 | 0.03631 | 0.09712 | 0.12245 | 0.10641 | 0.11401 | 0.13765 | 0.11907 | 0.10218 | 0.07178 | 0.04645 |
| Liaoning | 0.00124 | 0.0041 | 0.00866 | 0.03234 | 0.08928 | 0.14349 | 0.2075 | 0.20043 | 0.14304 | 0.09657 | 0.04328 | 0.03007 |
| Chongqing | 0.00704 | 0.01805 | 0.03698 | 0.11314 | 0.10654 | 0.06119 | 0.1039 | 0.14022 | 0.14968 | 0.11754 | 0.08188 | 0.06383 |
| Shaanxi | 0.00467 | 0.014 | 0.02893 | 0.05926 | 0.10079 | 0.10546 | 0.20019 | 0.22632 | 0.12552 | 0.0658 | 0.04013 | 0.02893 |
| Heilongjiang | 0.00096 | 0.00156 | 0.00471 | 0.0204 | 0.03819 | 0.07272 | 0.16324 | 0.17527 | 0.17082 | 0.17056 | 0.11667 | 0.06488 |

**Table S5. Epidemic Months, Non-Epidemic Months, and Onset Months of MP in 29 Provinces from April 2023 to March 2024**

| **Province** | **Apr-23** | **May-23** | **Jun-23** | **Jul-23** | **Aug-23** | **Sep-23** | **Oct-23** | **Nov-23** | **Dec-23** | **Jan-24** | **Feb-24** | **Mar-24** |
| --- | --- | --- | --- | --- | --- | --- | --- | --- | --- | --- | --- | --- |
| Shanghai | N(0) | N(0) | N(0) | O(1) | E(1) | E(1) | E(1) | E(1) | E(1) | E(0.41669) | N(0) | N(0) |
| Yunnan | N(0) | N(0) | N(0) | N(0) | N(0) | N(0) | O(0.60672) | E(1) | E(1) | E(1) | E(1) | E(1) |
| Neimongol | N(0) | N(0) | N(0) | N(0) | N(0) | N(0) | O(1) | E(1) | E(1) | E(1) | E(1) | E(0.60153) |
| Beijing | N(0) | N(0) | N(0) | N(0) | N(0) | N(0) | O(1) | E(1) | E(1) | E(1) | E(1) | E(0.21854) |
| Jilin | N(0) | N(0) | N(0) | N(0) | N(0) | O(1) | E(0.92581) | E(1) | E(1) | E(1) | N(0) | N(0) |
| Sichuan | N(0) | N(0) | N(0) | O(1) | E(1) | N(0) | E(1) | E(1) | E(1) | E(1) | E(0.49797) | N(0) |
| Tianjin | N(0) | N(0) | N(0) | N(0) | O(1) | E(1) | E(1) | E(1) | E(1) | E(0.49007) | N(0) | N(0) |
| Ningxia | N(0) | N(0) | N(0) | N(0) | N(0) | N(0) | O(0.55259) | E(1) | E(1) | E(1) | E(1) | E(1) |
| Anhui | N(0) | O(0.89702) | E(1) | E(1) | E(1) | E(1) | E(1) | E(1) | N(0) | N(0) | N(0) | N(0) |
| Guangdong | N(0) | N(0) | N(0) | N(0) | N(0) | N(0) | O(1) | E(1) | E(1) | E(1) | E(1) | E(0.60925) |
| Shanxi | N(0) | N(0) | N(0) | N(0) | O(0.88758) | N(0) | E(1) | E(1) | E(1) | E(1) | E(1) | N(0) |
| Guangdong | N(0) | N(0) | N(0) | O(1) | E(1) | E(0.78166) | E(1) | E(1) | E(1) | E(1) | N(0) | N(0) |
| Guangxi | N(0) | N(0) | N(0) | O(1) | E(1) | E(0.24527) | E(1) | E(1) | E(1) | E(1) | E(1) | N(0) |
| Xinjiang | N(0) | N(0) | N(0) | N(0) | N(0) | O(1) | E(0.86104) | E(1) | N(0) | E(1) | E(1) | E(1) |
| Jiangsu | N(0) | N(0) | N(0) | O(1) | E(1) | E(1) | E(1) | E(1) | E(1) | E(0.65778) | N(0) | N(0) |
| Jiangxi | N(0) | N(0) | N(0) | O(1) | E(1) | E(1) | E(1) | E(1) | E(1) | E(0.72912) | N(0) | N(0) |
| Hebei | N(0) | N(0) | N(0) | N(0) | O(0.45678) | E(1) | E(1) | E(1) | E(1) | E(1) | N(0) | N(0) |
| Henan | N(0) | N(0) | N(0) | N(0) | O(0.11395) | E(1) | E(1) | E(1) | E(1) | E(1) | E(1) | N(0) |
| Zhejiang | N(0) | N(0) | N(0) | O(1) | E(1) | E(1) | E(1) | E(1) | E(1) | E(1) | E(0.0494) | N(0) |
| Hainan | N(0) | N(0) | N(0) | N(0) | O(0.98303) | N(0) | E(1) | E(1) | E(1) | E(1) | E(1) | N(0) |
| Hubei | N(0) | N(0) | N(0) | O(1) | E(1) | E(1) | E(1) | E(1) | E(1) | E(0.4497) | N(0) | N(0) |
| Hunan | N(0) | N(0) | N(0) | O(1) | E(1) | E(0.57115) | E(1) | E(1) | E(1) | E(1) | N(0) | N(0) |
| Gansu | N(0) | N(0) | E(0) | E(0) | E(0) | N(0) | N(0) | O(1) | N(0) | E(1) | E(1) | E(1) |
| Fujian | N(0) | N(0) | N(0) | O(1) | E(1) | E(1) | E(1) | E(1) | E(1) | E(1) | E(0.19601) | N(0) |
| Guizhou | N(0) | N(0) | N(0) | O(0.4966) | E(1) | E(1) | E(1) | E(1) | E(1) | E(1) | N(0) | N(0) |
| Liaoning | N(0) | N(0) | N(0) | N(0) | N(0) | O(1) | E(1) | E(1) | E(1) | E(0.57513) | N(0) | N(0) |
| Chongqing | N(0) | N(0) | N(0) | O(1) | E(1) | N(0) | E(1) | E(1) | E(1) | E(1) | E(0.2318) | N(0) |
| Shaanxi | N(0) | N(0) | N(0) | N(0) | O(0.91785) | E(1) | E(1) | E(1) | E(1) | N(0) | N(0) | N(0) |
| Heilongjiang | N(0) | N(0) | N(0) | N(0) | N(0) | N(0) | O(1) | E(1) | E(1) | E(1) | E(0.60093) | N(0) |

N: Non-epidemic Month; O: Onset Month; E: Epidemic Month, the numbers in parentheses representing the percentage of months identified as epidemic months.

**Table S6. Validation of the posterior probabilities for provincial cluster assignments using Gaussian Mixture Models (GMM).**

| prov | Cluster | Prob_Cluster_1 | Prob_Cluster_2 | Prob_Cluster_3 | prov | Cluster | Prob_Cluster_1 | Prob_Cluster_2 | Prob_Cluster_3 |
| --- | --- | --- | --- | --- | --- | --- | --- | --- | --- |
| Shanghai | 1 | 1 | 0 | 0 | Shandong | 3 | 2.1766E-151 | 0 | 1 |
| Sichuan | 1 | 1 | 0 | 0 | Shanxi | 3 | 1.6037E-201 | 0 | 1 |
| Anhui | 1 | 1 | 0 | 0 | Hebei | 3 | 0 | 0 | 1 |
| Guangdong | 1 | 1 | 0 | 0 | Henan | 3 | 4.5282E-179 | 0 | 1 |
| Guangxi | 1 | 1 | 0 | 0 | Liaoning | 3 | 5.04602E-78 | 0 | 1 |
| Jiangsu | 1 | 1 | 0 | 0 | Shaanxi | 3 | 0 | 0 | 1 |
| Jiangxi | 1 | 1 | 0 | 0 | Heilongjiang | 3 | 0 | 0 | 1 |
| Zhejiang | 1 | 1 | 0 | 0 | Yunnan | 2 | 1.5692E-108 | 1 | 0 |
| Hubei | 1 | 1 | 0 | 0 | Neimongol | 2 | 0 | 1 | 0 |
| Hunan | 1 | 1 | 0 | 0 | Beijing | 2 | 2.417E-194 | 1 | 0 |
| Fujian | 1 | 1 | 0 | 0 | Ningxia | 2 | 7.4942E-138 | 1 | 0 |
| Guizhou | 1 | 1 | 0 | 0 | Xinjiang | 2 | 4.9352E-158 | 1 | 0 |
| Chongqing | 1 | 1 | 0 | 0 | Hainan | 2 | 1.14652E-59 | 1 | 0 |
| Jilin | 3 | 1.2389E-120 | 0 | 1 | Gansu | 2 | 4.0649E-163 | 1 | 0 |
| Tianjin | 3 | 0 | 0 | 1 |  |  |  |  |  |

**Table S7. Ratio of ARI and MP positive cases between males and females across each age**

|  | **ARI cases** | | | | | **MP positive cases** | | | | | **General population of China** |
| --- | --- | --- | --- | --- | --- | --- | --- | --- | --- | --- | --- |
| **Age** | **All** | **Male** | **Female** | **Male/Female** | **Normalized ratio**  **(Male/Female)** | **All** | **Male** | **Female** | **Male/Female** | **Normalized ratio**  **(Male/Female)** | **Male/Female** |
| 0 | 201026 | 125161 | 75865 | 1.6498 | 1.4834 | 12421 | 8067 | 4354 | 1.8528 | 1.6659 | 1.1122 |
| 1 | 123623 | 74840 | 48783 | 1.5341 | 1.3847 | 13804 | 8344 | 5460 | 1.5282 | 1.3794 | 1.1079 |
| 2 | 92919 | 53519 | 39400 | 1.3584 | 1.2273 | 14017 | 8049 | 5968 | 1.3487 | 1.2186 | 1.1068 |
| 3 | 121892 | 66637 | 55255 | 1.2060 | 1.0910 | 21461 | 11617 | 9844 | 1.1801 | 1.0676 | 1.1054 |
| 4 | 101131 | 54535 | 46596 | 1.1704 | 1.0477 | 25288 | 13450 | 11838 | 1.1362 | 1.0171 | 1.1171 |
| 5 | 75540 | 40678 | 34862 | 1.1668 | 1.0358 | 26318 | 13939 | 12379 | 1.1260 | 0.9996 | 1.1265 |
| 6 | 82318 | 43554 | 38764 | 1.1236 | 0.9900 | 37804 | 19453 | 18351 | 1.0601 | 0.9340 | 1.1349 |
| 7 | 69730 | 36919 | 32811 | 1.1252 | 0.9855 | 35747 | 18375 | 17372 | 1.0577 | 0.9264 | 1.1418 |
| 8 | 46043 | 24427 | 21616 | 1.1300 | 0.9851 | 24144 | 12442 | 11702 | 1.0632 | 0.9269 | 1.1471 |
| 9 | 39153 | 20981 | 18172 | 1.1546 | 1.0025 | 20713 | 10658 | 10055 | 1.0600 | 0.9204 | 1.1517 |
| 10 | 26770 | 14430 | 12340 | 1.1694 | 1.0175 | 13256 | 6834 | 6422 | 1.0642 | 0.9259 | 1.1493 |
| 11 | 19802 | 10841 | 8961 | 1.2098 | 1.0532 | 8861 | 4573 | 4288 | 1.0665 | 0.9284 | 1.1487 |
| 12 | 14199 | 7927 | 6272 | 1.2639 | 1.0989 | 4995 | 2604 | 2391 | 1.0891 | 0.9469 | 1.1501 |
| 13 | 11313 | 6230 | 5083 | 1.2257 | 1.0643 | 2942 | 1480 | 1462 | 1.0123 | 0.8790 | 1.1516 |
| 14 | 5667 | 3057 | 2610 | 1.1713 | 1.0106 | 1516 | 749 | 767 | 0.9765 | 0.8426 | 1.1590 |
| 15 | 4364 | 2399 | 1965 | 1.2209 | 1.0461 | 1158 | 547 | 611 | 0.8953 | 0.7671 | 1.1671 |
| 16 | 3435 | 1866 | 1569 | 1.1893 | 1.0171 | 994 | 454 | 540 | 0.8407 | 0.7190 | 1.1693 |
| 17 | 2868 | 1595 | 1273 | 1.2529 | 1.0744 | 830 | 413 | 417 | 0.9904 | 0.8493 | 1.1662 |
| 18 | 1850 | 1029 | 821 | 1.2533 | 1.0946 | 444 | 225 | 219 | 1.0274 | 0.8973 | 1.1450 |
| 19 | 1356 | 751 | 605 | 1.2413 | 1.0935 | 333 | 161 | 172 | 0.9360 | 0.8246 | 1.1352 |
| 20 | 1258 | 715 | 543 | 1.3168 | 1.1665 | 296 | 149 | 147 | 1.0136 | 0.8979 | 1.1288 |
| 21 | 1336 | 744 | 592 | 1.2568 | 1.1157 | 275 | 134 | 141 | 0.9504 | 0.8437 | 1.1264 |
| 22 | 1483 | 800 | 683 | 1.1713 | 1.0439 | 289 | 134 | 155 | 0.8645 | 0.7705 | 1.1220 |
| 23 | 1661 | 869 | 792 | 1.0972 | 0.9848 | 336 | 129 | 207 | 0.6232 | 0.5593 | 1.1142 |
| 24 | 1765 | 887 | 878 | 1.0103 | 0.9054 | 351 | 131 | 220 | 0.5955 | 0.5337 | 1.1158 |
| 25 | 2026 | 1006 | 1020 | 0.9863 | 0.8900 | 398 | 149 | 249 | 0.5984 | 0.5400 | 1.1082 |
| 26 | 2077 | 966 | 1111 | 0.8695 | 0.7899 | 455 | 145 | 310 | 0.4677 | 0.4249 | 1.1008 |
| 27 | 2262 | 1110 | 1152 | 0.9635 | 0.8775 | 467 | 158 | 309 | 0.5113 | 0.4657 | 1.0980 |
| 28 | 2562 | 1221 | 1341 | 0.9105 | 0.8334 | 531 | 208 | 323 | 0.6440 | 0.5894 | 1.0925 |
| 29 | 2622 | 1266 | 1356 | 0.9336 | 0.8674 | 570 | 208 | 362 | 0.5746 | 0.5339 | 1.0763 |
| 30 | 2855 | 1334 | 1521 | 0.8771 | 0.8276 | 617 | 222 | 395 | 0.5620 | 0.5304 | 1.0597 |
| 31 | 3089 | 1485 | 1604 | 0.9258 | 0.8784 | 682 | 271 | 411 | 0.6594 | 0.6256 | 1.0540 |
| 32 | 3272 | 1577 | 1695 | 0.9304 | 0.8825 | 695 | 258 | 437 | 0.5904 | 0.5600 | 1.0542 |
| 33 | 4201 | 2024 | 2177 | 0.9297 | 0.8828 | 898 | 356 | 542 | 0.6568 | 0.6237 | 1.0531 |
| 34 | 4170 | 2018 | 2152 | 0.9377 | 0.8848 | 889 | 302 | 587 | 0.5145 | 0.4855 | 1.0598 |
| 35 | 4127 | 2077 | 2050 | 1.0132 | 0.9557 | 859 | 345 | 514 | 0.6712 | 0.6332 | 1.0601 |
| 36 | 4340 | 2196 | 2144 | 1.0243 | 0.9650 | 909 | 369 | 540 | 0.6833 | 0.6438 | 1.0614 |
| 37 | 3907 | 1999 | 1908 | 1.0477 | 0.9884 | 735 | 274 | 461 | 0.5944 | 0.5607 | 1.0600 |
| 38 | 3547 | 1829 | 1718 | 1.0646 | 1.0090 | 636 | 267 | 369 | 0.7236 | 0.6858 | 1.0551 |
| 39 | 3554 | 1977 | 1577 | 1.2536 | 1.1904 | 600 | 274 | 326 | 0.8405 | 0.7981 | 1.0531 |
| 40 | 3620 | 1981 | 1639 | 1.2087 | 1.1441 | 503 | 218 | 285 | 0.7649 | 0.7241 | 1.0564 |
| 41 | 3784 | 2108 | 1676 | 1.2578 | 1.2021 | 530 | 226 | 304 | 0.7434 | 0.7105 | 1.0463 |
| 42 | 3459 | 1932 | 1527 | 1.2652 | 1.2046 | 385 | 170 | 215 | 0.7907 | 0.7528 | 1.0503 |
| 43 | 3494 | 2010 | 1484 | 1.3544 | 1.2915 | 315 | 136 | 179 | 0.7598 | 0.7245 | 1.0487 |
| 44 | 3760 | 2174 | 1586 | 1.3707 | 1.3164 | 363 | 171 | 192 | 0.8906 | 0.8553 | 1.0413 |
| 45 | 3751 | 2245 | 1506 | 1.4907 | 1.4378 | 297 | 140 | 157 | 0.8917 | 0.8601 | 1.0368 |
| 46 | 3860 | 2290 | 1570 | 1.4586 | 1.4025 | 271 | 107 | 164 | 0.6524 | 0.6273 | 1.0400 |
| 47 | 4404 | 2685 | 1719 | 1.5620 | 1.5064 | 310 | 145 | 165 | 0.8788 | 0.8475 | 1.0369 |
| 48 | 4956 | 3028 | 1928 | 1.5705 | 1.5132 | 316 | 147 | 169 | 0.8698 | 0.8381 | 1.0379 |
| 49 | 5653 | 3378 | 2275 | 1.4848 | 1.4580 | 371 | 183 | 188 | 0.9734 | 0.9558 | 1.0184 |
| 50 | 6245 | 3723 | 2522 | 1.4762 | 1.4474 | 362 | 164 | 198 | 0.8283 | 0.8121 | 1.0199 |
| 51 | 6904 | 4183 | 2721 | 1.5373 | 1.5146 | 417 | 208 | 209 | 0.9952 | 0.9805 | 1.0150 |
| 52 | 7494 | 4528 | 2966 | 1.5266 | 1.5094 | 389 | 167 | 222 | 0.7523 | 0.7438 | 1.0114 |
| 53 | 8004 | 4811 | 3193 | 1.5067 | 1.4746 | 462 | 211 | 251 | 0.8406 | 0.8227 | 1.0218 |
| 54 | 8476 | 5108 | 3368 | 1.5166 | 1.4917 | 466 | 216 | 250 | 0.8640 | 0.8498 | 1.0167 |
| 55 | 9157 | 5507 | 3650 | 1.5088 | 1.5029 | 519 | 242 | 277 | 0.8736 | 0.8703 | 1.0039 |
| 56 | 8500 | 5245 | 3255 | 1.6114 | 1.5847 | 442 | 208 | 234 | 0.8889 | 0.8742 | 1.0168 |
| 57 | 9495 | 5786 | 3709 | 1.5600 | 1.5612 | 498 | 226 | 272 | 0.8309 | 0.8315 | 0.9992 |
| 58 | 10048 | 6225 | 3823 | 1.6283 | 1.6954 | 545 | 240 | 305 | 0.7869 | 0.8193 | 0.9604 |
| 59 | 10919 | 6849 | 4070 | 1.6828 | 1.6868 | 507 | 226 | 281 | 0.8043 | 0.8062 | 0.9976 |
| 60 | 12838 | 8037 | 4801 | 1.6740 | 1.6303 | 616 | 287 | 329 | 0.8723 | 0.8496 | 1.0268 |
| 61 | 10277 | 6536 | 3741 | 1.7471 | 1.7025 | 444 | 223 | 221 | 1.0090 | 0.9833 | 1.0262 |
| 62 | 6840 | 4449 | 2391 | 1.8607 | 1.8341 | 264 | 137 | 127 | 1.0787 | 1.0633 | 1.0145 |
| 63 | 7590 | 4989 | 2601 | 1.9181 | 1.9451 | 296 | 154 | 142 | 1.0845 | 1.0998 | 0.9861 |
| 64 | 8430 | 5578 | 2852 | 1.9558 | 1.9780 | 335 | 181 | 154 | 1.1753 | 1.1886 | 0.9888 |
| 65 | 11316 | 7413 | 3903 | 1.8993 | 1.9564 | 392 | 209 | 183 | 1.1421 | 1.1764 | 0.9708 |
| 66 | 12760 | 8384 | 4376 | 1.9159 | 2.0030 | 463 | 241 | 222 | 1.0856 | 1.1350 | 0.9565 |
| 67 | 12305 | 8001 | 4304 | 1.8590 | 1.9425 | 426 | 228 | 198 | 1.1515 | 1.2033 | 0.9570 |
| 68 | 13325 | 8707 | 4618 | 1.8854 | 1.9967 | 415 | 220 | 195 | 1.1282 | 1.1948 | 0.9443 |
| 69 | 13733 | 8922 | 4811 | 1.8545 | 1.9417 | 460 | 254 | 206 | 1.2330 | 1.2910 | 0.9551 |
| 70 | 13441 | 8783 | 4658 | 1.8856 | 1.9367 | 425 | 235 | 190 | 1.2368 | 1.2704 | 0.9736 |
| 71 | 13223 | 8636 | 4587 | 1.8827 | 1.9929 | 395 | 230 | 165 | 1.3939 | 1.4755 | 0.9447 |
| 72 | 12262 | 8106 | 4156 | 1.9504 | 2.0600 | 380 | 224 | 156 | 1.4359 | 1.5166 | 0.9468 |
| 73 | 12219 | 8074 | 4145 | 1.9479 | 2.1118 | 331 | 206 | 125 | 1.6480 | 1.7866 | 0.9224 |
| 74 | 12180 | 8158 | 4022 | 2.0283 | 2.2371 | 309 | 185 | 124 | 1.4919 | 1.6455 | 0.9067 |
| 75 | 10826 | 7117 | 3709 | 1.9188 | 2.0882 | 294 | 163 | 131 | 1.2443 | 1.3541 | 0.9189 |
| 76 | 10666 | 7081 | 3585 | 1.9752 | 2.2183 | 277 | 168 | 109 | 1.5413 | 1.7310 | 0.8904 |
| 77 | 9689 | 6406 | 3283 | 1.9513 | 2.2133 | 256 | 162 | 94 | 1.7234 | 1.9549 | 0.8816 |
| 78 | 8536 | 5580 | 2956 | 1.8877 | 2.1718 | 213 | 133 | 80 | 1.6625 | 1.9127 | 0.8692 |
| 79 | 8228 | 5362 | 2866 | 1.8709 | 2.1803 | 185 | 113 | 72 | 1.5694 | 1.8290 | 0.8581 |
| 80 | 7670 | 4936 | 2734 | 1.8054 | 2.1932 | 155 | 91 | 64 | 1.4219 | 1.7273 | 0.8232 |
| 80+ | 56884 | 36474 | 20410 | 1.7871 | 2.0825 | 946 | 559 | 387 | 1.4444 | 1.6833 | 0.8581 |

**Table S8. Number of MP cases and MP positivity rate among males and females of each age in all cases, URTI cases, and LRTI cases.**

|  | **All cases** | | | **Upper respiratory tract** | | | **Lower respiratory tract** | | |
| --- | --- | --- | --- | --- | --- | --- | --- | --- | --- |
| **Age** | **All cases** | **MP positive** | **Positivity rate** | **All cases** | **MP positive** | **Positivity rate** | **All cases** | **MP positive** | **Positivity rate** |
| 0 | 221905 | 13631 | 6.143% | 187278 | 9306 | 4.969% | 34627 | 4325 | 12.490% |
| 1 | 134702 | 14958 | 11.105% | 119644 | 10917 | 9.125% | 15058 | 4041 | 26.836% |
| 2 | 101288 | 15089 | 14.897% | 90624 | 11450 | 12.635% | 10664 | 3639 | 34.124% |
| 3 | 133478 | 23020 | 17.246% | 118471 | 17262 | 14.571% | 15007 | 5758 | 38.369% |
| 4 | 109446 | 26747 | 24.439% | 95352 | 19781 | 20.745% | 14094 | 6966 | 49.425% |
| 5 | 82569 | 27964 | 33.867% | 70410 | 20323 | 28.864% | 12159 | 7641 | 62.842% |
| 6 | 87520 | **39498** | 45.130% | 72446 | **28661** | 39.562% | 15074 | **10837** | 71.892% |
| 7 | 73491 | **37277** | 50.723% | 59997 | **26860** | 44.769% | 13494 | **10417** | 77.197% |
| 8 | 49037 | 25481 | 51.963% | 39565 | 18231 | 46.079% | 9472 | 7250 | 76.541% |
| 9 | 41323 | 21599 | 52.269% | 33362 | 15540 | 46.580% | 7961 | 6059 | 76.109% |
| 10 | 28425 | 13836 | 48.675% | 22965 | 9946 | 43.309% | 5460 | 3890 | 71.245% |
| 11 | 20871 | 9206 | 44.109% | 16806 | 6571 | 39.099% | 4065 | 2635 | 64.822% |
| 12 | 15084 | 5224 | 34.633% | 12287 | 3651 | 29.714% | 2797 | 1573 | 56.239% |
| 13 | 12060 | 3105 | 25.746% | 9689 | 2025 | 20.900% | 2371 | 1080 | 45.550% |
| 14 | 6093 | 1630 | 26.752% | 3761 | 682 | 18.133% | 2332 | 948 | 40.652% |
| 15 | 4748 | 1268 | 26.706% | 2174 | 349 | 16.053% | 2574 | 919 | 35.703% |
| 16 | 3825 | 1102 | 28.810% | 1314 | 208 | 15.830% | 2511 | 894 | 35.603% |
| 17 | 3236 | 945 | 29.203% | 876 | 136 | 15.525% | 2360 | 809 | 34.280% |
| 18 | 2148 | 504 | 23.464% | 421 | 55 | 13.064% | 1727 | 449 | 25.999% |
| 19 | 1557 | 378 | 24.277% | 267 | 31 | 11.610% | 1290 | 347 | 26.899% |
| 20 | 1449 | 331 | 22.843% | 235 | 23 | 9.787% | 1214 | 308 | 25.371% |
| 21 | 1604 | 309 | 19.264% | 271 | 24 | 8.856% | 1333 | 285 | 21.380% |
| 22 | 1739 | 334 | 19.206% | 281 | 34 | 12.100% | 1458 | 300 | 20.576% |
| 23 | 1926 | 377 | 19.574% | 350 | 35 | 10.000% | 1576 | 342 | 21.701% |
| 24 | 2106 | 391 | 18.566% | 406 | 27 | 6.650% | 1700 | 364 | 21.412% |
| 25 | 2360 | 446 | 18.898% | 444 | 32 | 7.207% | 1916 | 414 | 21.608% |
| 26 | 2440 | 509 | 20.861% | 443 | 56 | 12.641% | 1997 | 453 | 22.684% |
| 27 | 2594 | 506 | 19.507% | 543 | 51 | 9.392% | 2051 | 455 | 22.184% |
| 28 | 2911 | 582 | 19.993% | 626 | 61 | 9.744% | 2285 | 521 | 22.801% |
| 29 | 3026 | 635 | 20.985% | 655 | 69 | 10.534% | 2371 | 566 | 23.872% |
| 30 | 3323 | 710 | 21.366% | 747 | 88 | 11.780% | 2576 | 622 | 24.146% |
| 31 | 3579 | 764 | 21.347% | 795 | 70 | 8.805% | 2784 | 694 | 24.928% |
| 32 | 3870 | 788 | 20.362% | 848 | 81 | 9.552% | 3022 | 707 | 23.395% |
| 33 | 4810 | 984 | 20.457% | 1125 | 100 | 8.889% | 3685 | 884 | 23.989% |
| 34 | 4755 | 971 | 20.421% | 1004 | 106 | 10.558% | 3751 | 865 | 23.061% |
| 35 | 4738 | 953 | 20.114% | 1002 | 114 | 11.377% | 3736 | 839 | 22.457% |
| 36 | 4927 | 980 | 19.890% | 1001 | 103 | 10.290% | 3926 | 877 | 22.338% |
| 37 | 4414 | 791 | 17.920% | 894 | 89 | 9.955% | 3520 | 702 | 19.943% |
| 38 | 4022 | 704 | 17.504% | 766 | 77 | 10.052% | 3256 | 627 | 19.257% |
| 39 | 4034 | 639 | 15.840% | 706 | 64 | 9.065% | 3328 | 575 | 17.278% |
| 40 | 4199 | 548 | 13.051% | 797 | 65 | 8.156% | 3402 | 483 | 14.198% |
| 41 | 4298 | 571 | 13.285% | 763 | 72 | 9.436% | 3535 | 499 | 14.116% |
| 42 | 3948 | 424 | 10.740% | 707 | 56 | 7.921% | 3241 | 368 | 11.355% |
| 43 | 4076 | 352 | 8.636% | 617 | 42 | 6.807% | 3459 | 310 | 8.962% |
| 44 | 4311 | 397 | 9.209% | 636 | 47 | 7.390% | 3675 | 350 | 9.524% |
| 45 | 4372 | 321 | 7.342% | 598 | 34 | 5.686% | 3774 | 287 | 7.605% |
| 46 | 4571 | 325 | 7.110% | 586 | 22 | 3.754% | 3985 | 303 | 7.604% |
| 47 | 5187 | 357 | 6.883% | 635 | 29 | 4.567% | 4552 | 328 | 7.206% |
| 48 | 5868 | 362 | 6.169% | 667 | 24 | 3.598% | 5201 | 338 | 6.499% |
| 49 | 6642 | 416 | 6.263% | 698 | 36 | 5.158% | 5944 | 380 | 6.393% |
| 50 | 7268 | 413 | 5.682% | 790 | 34 | 4.304% | 6478 | 379 | 5.851% |
| 51 | 8066 | 472 | 5.852% | 816 | 39 | 4.779% | 7250 | 433 | 5.972% |
| 52 | 8791 | 447 | 5.085% | 919 | 35 | 3.808% | 7872 | 412 | 5.234% |
| 53 | 9354 | 523 | 5.591% | 914 | 30 | 3.282% | 8440 | 493 | 5.841% |
| 54 | 9838 | 532 | 5.408% | 952 | 25 | 2.626% | 8886 | 507 | 5.706% |
| 55 | 10484 | 577 | 5.504% | 1004 | 29 | 2.888% | 9480 | 548 | 5.781% |
| 56 | 9960 | 502 | 5.040% | 975 | 31 | 3.179% | 8985 | 471 | 5.242% |
| 57 | 11090 | 565 | 5.095% | 1043 | 38 | 3.643% | 10047 | 527 | 5.245% |
| 58 | 11798 | 632 | 5.357% | 1083 | 43 | 3.970% | 10715 | 589 | 5.497% |
| 59 | 12906 | 588 | 4.556% | 1248 | 43 | 3.446% | 11658 | 545 | 4.675% |
| 60 | 14219 | 672 | 4.726% | 1452 | 52 | 3.581% | 12767 | 620 | 4.856% |
| 61 | 11214 | 484 | 4.316% | 1171 | 44 | 3.757% | 10043 | 440 | 4.381% |
| 62 | 8044 | 314 | 3.904% | 795 | 26 | 3.270% | 7249 | 288 | 3.973% |
| 63 | 8954 | 336 | 3.753% | 821 | 34 | 4.141% | 8133 | 302 | 3.713% |
| 64 | 10271 | 383 | 3.729% | 983 | 31 | 3.154% | 9288 | 352 | 3.790% |
| 65 | 13161 | 438 | 3.328% | 1283 | 33 | 2.572% | 11878 | 405 | 3.410% |
| 66 | 14585 | 520 | 3.565% | 1450 | 40 | 2.759% | 13135 | 480 | 3.654% |
| 67 | 14340 | 487 | 3.396% | 1420 | 27 | 1.901% | 12920 | 460 | 3.560% |
| 68 | 15286 | 471 | 3.081% | 1502 | 35 | 2.330% | 13784 | 436 | 3.163% |
| 69 | 15660 | 508 | 3.244% | 1601 | 35 | 2.186% | 14059 | 473 | 3.364% |
| 70 | 15323 | 469 | 3.061% | 1585 | 46 | 2.902% | 13738 | 423 | 3.079% |
| 71 | 14939 | 433 | 2.898% | 1680 | 41 | 2.440% | 13259 | 392 | 2.956% |
| 72 | 13964 | 439 | 3.144% | 1470 | 34 | 2.313% | 12494 | 405 | 3.242% |
| 73 | 13877 | 377 | 2.717% | 1559 | 38 | 2.437% | 12318 | 339 | 2.752% |
| 74 | 13671 | 350 | 2.560% | 1544 | 26 | 1.684% | 12127 | 324 | 2.672% |
| 75 | 12261 | 330 | 2.691% | 1432 | 24 | 1.676% | 10829 | 306 | 2.826% |
| 76 | 12060 | 315 | 2.612% | 1396 | 33 | 2.364% | 10664 | 282 | 2.644% |
| 77 | 10916 | 291 | 2.666% | 1327 | 30 | 2.261% | 9589 | 261 | 2.722% |
| 78 | 9678 | 242 | 2.501% | 1179 | 33 | 2.799% | 8499 | 209 | 2.459% |
| 79 | 9294 | 209 | 2.249% | 1195 | 32 | 2.678% | 8099 | 177 | 2.185% |
| 80 | 8619 | 165 | 1.914% | 1241 | 17 | 1.370% | 7378 | 148 | 2.006% |
| 80+ | 64164 | 1046 | 1.630% | 10266 | 181 | 1.763% | 53898 | 865 | 1.605% |

**Table S9. The ratio of male to female positive cases for each age group across 15 respiratory pathogens, with the two numbers in parentheses representing the number of male and female positive cases, respectively.**

| **age** | ABA | BP | HIN | Hadv | HHV | HMPV | HPIV | HRV | IAV | IBV | KPN | MCAT | RSV | SA | SPN |
| --- | --- | --- | --- | --- | --- | --- | --- | --- | --- | --- | --- | --- | --- | --- | --- |
| **0** | 1.666(21992/13202) | 1.420(6034/4249) | 1.752(33573/19165) | 1.853(4747/2562) | 1.483(1066/719) | 1.993(9302/4668) | 1.756(16868/9608) | 1.855(30162/16257) | 1.659(5049/3044) | 1.655(2899/1752) | 1.710(11930/6976) | 1.619(21438/13241) | 1.631(39501/24213) | 1.724(25512/14798) | 1.680(21961/13074) |
| **1** | 1.597(10204/6388) | 1.316(990/752) | 1.564(26942/17227) | 1.593(7511/4716) | 1.315(2236/1701) | 1.508(6267/4155) | 1.604(10883/6786) | 1.746(18214/10433) | 1.569(4149/2645) | 1.524(2412/1583) | 1.638(3588/2190) | 1.528(13767/9008) | 1.515(17994/11875) | 1.606(8489/5286) | 1.635(19707/12053) |
| **2** | 1.402(6092/4346) | 1.379(451/327) | 1.370(19688/14367) | 1.424(5536/3887) | 1.167(1563/1339) | 1.264(5116/4046) | 1.331(7048/5296) | 1.507(12706/8434) | 1.455(3438/2363) | 1.386(1738/1254) | 1.434(1834/1279) | 1.349(11183/8291) | 1.291(12075/9354) | 1.380(5605/4061) | 1.405(13158/9363) |
| **3** | 1.248(6611/5298) | 1.042(597/573) | 1.246(27236/21857) | 1.310(7708/5885) | 1.110(1722/1552) | 1.084(6948/6407) | 1.196(8598/7188) | 1.280(18281/14281) | 1.275(5041/3954) | 1.255(1981/1579) | 1.256(1775/1413) | 1.230(18833/15307) | 1.111(12083/10874) | 1.245(6523/5241) | 1.235(22756/18423) |
| **4** | 1.165(4750/4076) | 1.060(1191/1124) | 1.235(24242/19632) | 1.272(6903/5428) | 1.113(1424/1280) | 1.072(5265/4913) | 1.154(5194/4499) | 1.233(13898/11273) | 1.324(4764/3598) | 1.238(2187/1767) | 1.138(1137/999) | 1.224(13260/10834) | 1.075(5852/5443) | 1.159(5372/4634) | 1.241(20085/16189) |
| **5** | 1.146(3317/2894) | 1.018(2010/1975) | 1.259(17420/13832) | 1.240(5050/4074) | 1.181(1245/1054) | 1.001(2486/2483) | 1.148(2541/2214) | 1.222(9461/7741) | 1.290(3848/2982) | 1.278(2202/1723) | 1.172(900/768) | 1.213(7049/5812) | 1.074(2387/2222) | 1.159(4469/3856) | 1.276(13170/10322) |
| **6** | 1.132(3224/2847) | 1.074(3014/2806) | 1.242(17091/13763) | 1.209(5356/4430) | 1.134(1452/1280) | 0.990(1992/2013) | 1.063(1819/1711) | 1.150(9044/7861) | 1.211(3808/3144) | 1.243(2770/2228) | 1.218(877/720) | 1.218(5272/4328) | 1.015(1866/1838) | 1.142(4724/4135) | 1.244(12238/9834) |
| **7** | 1.157(2562/2215) | 1.092(2708/2479) | 1.248(13677/10956) | 1.263(4389/3476) | 1.170(1265/1081) | 1.083(1336/1234) | 1.154(1395/1209) | 1.185(7307/6165) | 1.272(2826/2221) | 1.203(2576/2142) | 1.280(740/578) | 1.203(3654/3037) | 1.039(1545/1487) | 1.123(3868/3444) | 1.291(9284/7189) |
| **8** | 1.098(1746/1590) | 1.132(1556/1375) | 1.222(8089/6618) | 1.202(2218/1845) | 1.042(851/817) | 1.113(805/723) | 1.030(826/802) | 1.213(4508/3716) | 1.305(2026/1553) | 1.251(1619/1294) | 1.110(526/474) | 1.337(1941/1452) | 1.113(1021/917) | 1.247(2844/2281) | 1.372(5441/3967) |
| **9** | 1.167(1574/1349) | 1.274(1182/928) | 1.301(6666/5122) | 1.277(1849/1448) | 1.215(882/726) | 1.103(676/613) | 1.142(709/621) | 1.318(3850/2922) | 1.193(1549/1298) | 1.230(1129/918) | 1.199(500/417) | 1.273(1312/1031) | 1.119(913/816) | 1.248(2479/1986) | 1.476(4153/2813) |
| **10** | 1.176(1162/988) | 1.124(688/612) | 1.320(4618/3499) | 1.288(1108/860) | 1.227(687/560) | 1.041(482/463) | 1.143(518/453) | 1.308(2611/1996) | 1.416(1147/810) | 1.319(761/577) | 1.238(395/319) | 1.325(803/606) | 0.993(556/560) | 1.264(1793/1419) | 1.400(2581/1843) |
| **11** | 1.180(905/767) | 1.224(454/371) | 1.389(3570/2571) | 1.383(867/627) | 1.177(551/468) | 1.191(399/335) | 1.205(411/341) | 1.332(1828/1372) | 1.361(860/632) | 1.267(575/454) | 1.125(341/303) | 1.299(517/398) | 1.182(494/418) | 1.406(1471/1046) | 1.515(1765/1165) |
| **12** | 1.317(707/537) | 1.454(317/218) | 1.507(3076/2041) | 1.541(570/370) | 1.310(465/355) | 1.327(333/251) | 1.322(349/264) | 1.450(1370/945) | 1.446(798/552) | 1.517(522/344) | 1.027(267/260) | 1.407(339/241) | 1.364(401/294) | 1.454(1066/733) | 1.674(1202/718) |
| **13** | 1.105(539/488) | 1.153(234/203) | 1.493(2777/1860) | 1.594(456/286) | 1.454(426/293) | 1.593(309/194) | 1.526(325/213) | 1.246(1028/825) | 1.460(651/446) | 1.420(443/312) | 1.197(304/254) | 1.689(255/151) | 1.452(363/250) | 1.250(821/657) | 1.920(958/499) |
| **14** | 1.161(245/211) | 1.274(93/73) | 1.536(1253/816) | 1.323(176/133) | 1.447(233/161) | 1.311(139/106) | 1.197(140/117) | 1.284(506/394) | 1.213(245/202) | 1.549(237/153) | 1.354(176/130) | 1.411(79/56) | 1.231(133/108) | 1.235(400/324) | 2.121(422/199) |
| **15** | 1.394(216/155) | 1.382(76/55) | 1.680(1001/596) | 1.400(133/95) | 1.318(199/151) | 1.414(123/87) | 1.392(110/79) | 1.471(434/295) | 1.314(230/175) | 1.733(130/75) | 1.756(151/86) | 0.957(44/46) | 1.389(132/95) | 1.464(350/239) | 2.146(294/137) |
| **16** | 1.133(111/98) | 1.214(51/42) | 1.761(766/435) | 1.911(107/56) | 1.263(168/133) | 1.383(83/60) | 1.237(73/59) | 1.606(347/216) | 1.350(166/123) | 1.241(98/79) | 1.377(106/77) | 1.400(28/20) | 1.593(86/54) | 1.477(260/176) | 2.789(212/76) |
| **17** | 1.529(104/68) | 1.440(36/25) | 1.821(570/313) | 1.812(87/48) | 1.713(149/87) | 1.462(76/52) | 2.370(64/27) | 1.442(235/163) | 1.219(117/96) | 1.429(80/56) | 1.329(97/73) | 1.444(26/18) | 0.925(49/53) | 1.620(222/137) | 2.559(174/68) |
| **18** | 1.821(71/39) | 1.250(15/12) | 1.979(285/144) | 1.931(56/29) | 1.553(118/76) | 1.042(25/24) | 2.800(42/15) | 1.713(149/87) | 1.580(79/50) | 1.269(33/26) | 1.305(77/59) | 0.923(12/13) | 1.739(40/23) | 1.210(144/119) | 2.275(91/40) |
| **19** | 1.710(53/31) | 2.000(8/4) | 1.667(155/93) | 1.958(47/24) | 1.836(101/55) | 3.167(19/6) | 0.765(13/17) | 1.492(97/65) | 0.771(27/35) | 2.077(27/13) | 1.390(57/41) | 1.000(11/11) | 0.750(12/16) | 1.295(114/88) | 2.333(56/24) |
| **20** | 1.676(57/34) | 3.000(9/3) | 1.585(130/82) | 1.300(26/20) | 2.140(107/50) | 4.250(17/4) | 0.929(13/14) | 1.704(92/54) | 1.242(41/33) | 2.444(22/9) | 2.179(61/28) | 1.444(13/9) | 1.417(17/12) | 1.276(97/76) | 1.750(49/28) |
| **21** | 1.462(57/39) | 4.000(4/1) | 1.325(106/80) | 1.870(43/23) | 1.486(107/72) | 1.364(15/11) | 1.545(17/11) | 1.265(62/49) | 1.058(55/52) | 1.000(23/23) | 1.220(61/50) | 1.182(13/11) | 0.944(17/18) | 1.694(105/62) | 1.524(32/21) |
| **22** | 1.109(51/46) | 1.333(4/3) | 1.484(138/93) | 1.440(36/25) | 1.568(116/74) | 0.867(13/15) | 1.462(19/13) | 1.147(78/68) | 0.884(61/69) | 1.737(33/19) | 1.549(79/51) | 1.400(14/10) | 0.909(20/22) | 1.241(98/79) | 2.292(55/24) |
| **23** | 1.463(60/41) | 1.333(8/6) | 1.126(125/111) | 1.727(38/22) | 1.920(144/75) | 0.630(17/27) | 0.900(18/20) | 1.403(101/72) | 1.000(62/62) | 1.357(38/28) | 1.508(92/61) | 0.929(13/14) | 2.083(25/12) | 1.306(111/85) | 0.812(39/48) |
| **24** | 1.471(50/34) | 0.222(2/9) | 1.039(133/128) | 2.190(46/21) | 1.378(124/90) | 1.158(22/19) | 0.643(18/28) | 0.926(75/81) | 0.890(73/82) | 1.027(38/37) | 1.673(87/52) | 0.882(15/17) | 1.043(24/23) | 0.965(109/113) | 1.525(61/40) |
| **25** | 1.172(68/58) | 1.600(8/5) | 0.886(132/149) | 1.027(38/37) | 1.207(134/111) | 0.476(20/42) | 1.250(30/24) | 0.894(101/113) | 0.938(75/80) | 1.191(56/47) | 1.507(101/67) | 0.375(12/32) | 1.043(24/23) | 1.008(119/118) | 0.966(57/59) |
| **26** | 1.510(74/49) | 1.091(12/11) | 0.759(126/166) | 1.111(40/36) | 1.622(146/90) | 0.553(21/38) | 0.606(20/33) | 0.951(98/103) | 0.649(50/77) | 0.870(47/54) | 1.620(115/71) | 0.517(15/29) | 1.000(21/21) | 1.037(113/109) | 1.114(49/44) |
| **27** | 1.394(92/66) | 1.333(12/9) | 0.951(176/185) | 1.333(56/42) | 1.288(152/118) | 0.679(19/28) | 0.917(22/24) | 0.801(117/146) | 0.639(69/108) | 0.877(50/57) | 1.486(107/72) | 0.515(17/33) | 0.885(23/26) | 1.111(130/117) | 0.965(55/57) |
| **28** | 1.064(83/78) | 0.857(12/14) | 0.870(188/216) | 1.304(73/56) | 1.242(164/132) | 0.765(26/34) | 1.000(34/34) | 0.917(122/133) | 0.612(79/129) | 0.886(62/70) | 1.420(125/88) | 0.606(20/33) | 0.750(30/40) | 1.042(149/143) | 0.744(58/78) |
| **29** | 1.130(87/77) | 0.615(8/13) | 0.704(176/250) | 1.167(70/60) | 1.336(183/137) | 0.571(28/49) | 0.786(22/28) | 0.745(102/137) | 0.890(97/109) | 0.811(60/74) | 1.937(153/79) | 0.794(27/34) | 0.558(24/43) | 0.952(138/145) | 0.809(76/94) |
| **30** | 1.034(91/88) | 0.571(12/21) | 0.747(207/277) | 1.472(78/53) | 1.623(185/114) | 0.509(28/55) | 0.629(22/35) | 0.833(125/150) | 0.455(60/132) | 0.693(70/101) | 1.731(161/93) | 0.586(34/58) | 0.953(41/43) | 1.080(149/138) | 0.848(78/92) |
| **31** | 1.198(109/91) | 0.810(17/21) | 0.902(257/285) | 1.132(77/68) | 1.669(227/136) | 0.678(40/59) | 0.620(31/50) | 0.938(135/144) | 0.720(85/118) | 0.717(76/106) | 1.705(162/95) | 0.567(34/60) | 0.863(44/51) | 0.918(167/182) | 1.031(101/98) |
| **32** | 1.570(124/79) | 0.769(20/26) | 0.760(266/350) | 1.062(86/81) | 1.705(237/139) | 0.677(42/62) | 0.766(36/47) | 0.769(143/186) | 1.065(131/123) | 0.849(79/93) | 1.532(167/109) | 0.600(36/60) | 0.870(40/46) | 1.215(175/144) | 1.036(114/110) |
| **33** | 1.256(157/125) | 0.535(23/43) | 0.899(349/388) | 0.807(92/114) | 1.708(304/178) | 0.542(52/96) | 0.982(54/55) | 0.765(179/234) | 0.911(133/146) | 0.783(101/129) | 1.788(245/137) | 0.537(44/82) | 0.918(56/61) | 1.020(207/203) | 0.935(143/153) |
| **34** | 1.369(152/111) | 0.535(23/43) | 0.918(358/390) | 1.053(99/94) | 1.521(295/194) | 0.612(41/67) | 0.851(40/47) | 0.968(210/217) | 0.795(124/156) | 1.078(125/116) | 1.828(245/134) | 0.506(45/89) | 0.841(58/69) | 1.098(202/184) | 1.020(152/149) |
| **35** | 1.500(162/108) | 0.750(36/48) | 0.933(389/417) | 1.214(119/98) | 1.860(320/172) | 0.667(46/69) | 1.093(47/43) | 0.880(191/217) | 1.047(134/128) | 0.746(94/126) | 2.330(261/112) | 0.646(51/79) | 0.971(67/69) | 0.971(201/207) | 0.814(140/172) |
| **36** | 1.102(140/127) | 0.538(28/52) | 0.906(397/438) | 0.931(95/102) | 1.955(307/157) | 0.553(47/85) | 0.650(39/60) | 1.043(217/208) | 1.000(141/141) | 0.781(107/137) | 2.046(266/130) | 0.658(48/73) | 0.662(47/71) | 1.276(231/181) | 1.120(149/133) |
| **37** | 1.339(166/124) | 0.761(35/46) | 0.917(342/373) | 1.778(112/63) | 1.880(329/175) | 0.683(43/63) | 0.833(40/48) | 0.927(179/193) | 1.294(141/109) | 0.770(77/100) | 2.486(271/109) | 0.629(44/70) | 0.881(52/59) | 1.308(221/169) | 1.537(166/108) |
| **38** | 1.065(131/123) | 0.833(35/42) | 1.110(324/292) | 1.200(84/70) | 1.497(262/175) | 0.618(34/55) | 0.923(36/39) | 0.879(160/182) | 1.157(133/115) | 0.810(81/100) | 1.557(218/140) | 0.927(51/55) | 1.122(55/49) | 1.222(187/153) | 1.381(145/105) |
| **39** | 1.890(155/82) | 1.212(40/33) | 1.099(354/322) | 1.527(84/55) | 2.277(296/130) | 0.854(41/48) | 1.091(48/44) | 1.037(168/162) | 1.243(128/103) | 1.220(111/91) | 2.902(238/82) | 0.654(34/52) | 1.163(57/49) | 1.338(198/148) | 1.637(167/102) |
| **40** | 1.551(152/98) | 0.767(33/43) | 1.186(363/306) | 1.696(95/56) | 1.821(306/168) | 0.780(39/50) | 0.976(41/42) | 1.271(183/144) | 1.008(127/126) | 1.528(110/72) | 2.528(268/106) | 1.068(47/44) | 1.302(56/43) | 1.196(195/163) | 1.479(139/94) |
| **41** | 1.410(165/117) | 1.071(30/28) | 1.166(359/308) | 1.510(77/51) | 1.592(304/191) | 0.562(36/64) | 1.167(49/42) | 1.112(169/152) | 1.145(150/131) | 1.220(100/82) | 2.873(293/102) | 0.980(50/51) | 0.815(53/65) | 1.380(218/158) | 2.282(178/78) |
| **42** | 1.483(175/118) | 1.345(39/29) | 1.179(349/296) | 1.686(86/51) | 1.819(302/166) | 0.776(45/58) | 0.951(39/41) | 1.285(185/144) | 1.058(145/137) | 1.241(103/83) | 2.140(291/136) | 1.051(41/39) | 0.968(60/62) | 1.185(186/157) | 1.844(142/77) |
| **43** | 1.833(176/96) | 0.720(18/25) | 1.161(325/280) | 1.472(78/53) | 1.858(301/162) | 1.040(52/50) | 1.234(58/47) | 1.068(141/132) | 1.156(141/122) | 1.355(103/76) | 2.467(296/120) | 1.222(44/36) | 0.851(57/67) | 1.324(196/148) | 2.087(144/69) |
| **44** | 1.774(204/115) | 1.156(37/32) | 1.494(387/259) | 1.200(84/70) | 2.011(350/174) | 0.786(44/56) | 1.459(54/37) | 1.134(169/149) | 1.246(162/130) | 1.319(91/69) | 2.435(336/138) | 1.071(45/42) | 0.750(54/72) | 1.379(211/153) | 1.695(161/95) |
| **45** | 1.726(202/117) | 1.647(28/17) | 1.473(411/279) | 1.611(87/54) | 2.289(364/159) | 0.947(54/57) | 0.891(49/55) | 1.158(161/139) | 1.286(144/112) | 1.243(92/74) | 2.943(362/123) | 0.694(34/49) | 1.000(62/62) | 1.341(177/132) | 2.194(158/72) |
| **46** | 2.031(197/97) | 1.150(23/20) | 1.641(420/256) | 1.554(87/56) | 2.154(377/175) | 1.209(52/43) | 1.039(53/51) | 1.261(179/142) | 1.484(181/122) | 1.458(105/72) | 2.395(352/147) | 1.140(49/43) | 0.687(57/83) | 1.792(224/125) | 2.162(147/68) |
| **47** | 2.099(275/131) | 1.000(24/24) | 1.475(447/303) | 1.369(89/65) | 2.342(473/202) | 0.737(42/57) | 1.345(74/55) | 1.293(194/150) | 1.616(202/125) | 1.202(107/89) | 2.481(449/181) | 0.974(38/39) | 1.228(70/57) | 1.702(223/131) | 2.586(181/70) |
| **48** | 1.900(266/140) | 1.250(30/24) | 1.520(520/342) | 1.213(91/75) | 2.391(514/215) | 0.830(44/53) | 1.508(95/63) | 1.509(243/161) | 1.311(215/164) | 1.558(134/86) | 3.195(524/164) | 1.140(65/57) | 1.120(93/83) | 1.686(258/153) | 2.726(229/84) |
| **49** | 2.007(293/146) | 1.160(29/25) | 1.525(607/398) | 1.493(106/71) | 2.244(543/242) | 0.675(54/80) | 1.368(78/57) | 1.440(298/207) | 1.395(272/195) | 1.110(121/109) | 3.021(571/189) | 1.170(55/47) | 1.354(107/79) | 1.570(259/165) | 2.126(236/111) |
| **50** | 1.635(314/192) | 0.886(31/35) | 1.628(700/430) | 1.532(118/77) | 2.091(577/276) | 0.921(70/76) | 0.953(81/85) | 1.389(289/208) | 1.127(276/245) | 1.144(119/104) | 2.377(599/252) | 1.519(79/52) | 1.105(116/105) | 1.593(325/204) | 2.221(271/122) |
| **51** | 2.031(388/191) | 1.156(37/32) | 1.576(747/474) | 1.860(160/86) | 2.081(697/335) | 0.676(71/105) | 1.190(100/84) | 1.434(347/242) | 1.264(316/250) | 1.122(138/123) | 2.707(693/256) | 1.239(83/67) | 1.238(130/105) | 1.564(344/220) | 2.431(333/137) |
| **52** | 1.888(389/206) | 1.303(43/33) | 1.527(814/533) | 1.670(152/91) | 2.328(766/329) | 0.625(65/104) | 0.886(101/114) | 1.689(407/241) | 1.456(348/239) | 1.360(151/111) | 2.602(791/304) | 1.014(75/74) | 1.017(118/116) | 1.511(331/219) | 2.602(346/133) |
| **53** | 1.615(415/257) | 1.600(48/30) | 1.478(863/584) | 1.363(154/113) | 2.218(794/358) | 0.717(86/120) | 0.966(114/118) | 1.663(399/240) | 1.418(400/282) | 1.263(149/118) | 2.834(853/301) | 1.295(101/78) | 1.152(144/125) | 1.567(387/247) | 2.208(371/168) |
| **54** | 1.996(475/238) | 0.717(33/46) | 1.378(940/682) | 1.305(171/131) | 2.287(860/376) | 0.690(89/129) | 1.121(148/132) | 1.288(403/313) | 1.348(399/296) | 1.346(144/107) | 2.510(866/345) | 1.151(107/93) | 1.023(135/132) | 1.519(392/258) | 2.025(399/197) |
| **55** | 2.121(509/240) | 1.161(36/31) | 1.331(1009/758) | 1.199(169/141) | 2.265(906/400) | 0.748(98/131) | 0.866(136/157) | 1.341(460/343) | 1.435(449/313) | 1.088(136/125) | 2.688(938/349) | 0.943(100/106) | 0.956(174/182) | 1.362(384/282) | 2.246(438/195) |
| **56** | 1.984(508/256) | 1.406(45/32) | 1.470(941/640) | 1.509(160/106) | 2.519(937/372) | 0.862(94/109) | 1.495(148/99) | 1.339(395/295) | 1.462(437/299) | 1.337(119/89) | 3.060(918/300) | 1.114(127/114) | 1.357(171/126) | 1.511(346/229) | 2.151(456/212) |
| **57** | 1.957(595/304) | 1.630(44/27) | 1.375(998/726) | 1.292(168/130) | 2.017(954/473) | 0.733(96/131) | 1.105(168/152) | 1.452(472/325) | 1.719(495/288) | 1.554(129/83) | 2.774(1043/376) | 1.180(118/100) | 1.179(184/156) | 1.446(418/289) | 2.220(484/218) |
| **58** | 1.943(581/299) | 0.892(33/37) | 1.489(1132/760) | 1.317(166/126) | 2.547(1146/450) | 1.000(138/138) | 1.150(146/127) | 1.408(466/331) | 1.535(511/333) | 1.238(104/84) | 2.667(1131/424) | 1.279(133/104) | 1.094(187/171) | 1.468(433/295) | 2.352(548/233) |
| **59** | 2.223(776/349) | 0.912(31/34) | 1.420(1169/823) | 1.241(175/141) | 2.526(1243/492) | 0.856(113/132) | 1.255(172/137) | 1.587(530/334) | 1.603(601/375) | 1.273(112/88) | 2.962(1256/424) | 1.513(180/119) | 1.147(195/170) | 1.534(448/292) | 1.940(516/266) |
| **60** | 2.061(845/410) | 1.340(63/47) | 1.441(1406/976) | 1.654(220/133) | 2.314(1439/622) | 1.055(172/163) | 1.234(227/184) | 1.578(609/386) | 1.643(685/417) | 1.470(169/115) | 2.482(1457/587) | 1.746(220/126) | 1.261(261/207) | 1.717(644/375) | 2.003(669/334) |
| **61** | 2.262(726/321) | 0.679(36/53) | 1.711(1261/737) | 1.445(198/137) | 2.403(1199/499) | 0.768(126/164) | 1.159(175/151) | 1.832(599/327) | 1.280(498/389) | 1.404(146/104) | 3.096(1257/406) | 1.626(161/99) | 1.656(265/160) | 1.888(457/242) | 2.313(539/233) |
| **62** | 2.450(539/220) | 1.706(29/17) | 1.697(784/462) | 1.370(111/81) | 2.610(796/305) | 0.867(85/98) | 1.681(116/69) | 1.613(363/225) | 1.792(423/236) | 1.746(110/63) | 3.191(852/267) | 1.706(116/68) | 1.411(151/107) | 1.684(288/171) | 2.810(354/126) |
| **63** | 2.687(626/233) | 1.214(34/28) | 1.540(793/515) | 1.564(122/78) | 2.762(950/344) | 0.931(94/101) | 1.436(145/101) | 1.735(366/211) | 1.817(456/251) | 1.633(129/79) | 3.577(1005/281) | 2.458(145/59) | 1.294(163/126) | 1.961(355/181) | 2.497(397/159) |
| **64** | 2.450(659/269) | 1.348(31/23) | 1.941(980/505) | 1.788(152/85) | 2.839(1005/354) | 1.260(121/96) | 1.298(135/104) | 1.730(403/233) | 1.658(484/292) | 1.633(129/79) | 3.111(1036/333) | 1.855(141/76) | 1.620(209/129) | 1.449(371/256) | 2.665(453/170) |
| **65** | 2.446(861/352) | 1.225(49/40) | 1.831(1335/729) | 1.735(196/113) | 2.599(1401/539) | 1.310(169/129) | 1.769(237/134) | 1.861(534/287) | 1.617(650/402) | 1.566(213/136) | 3.139(1359/433) | 1.568(207/132) | 1.488(256/172) | 1.710(460/269) | 2.635(614/233) |
| **66** | 2.625(1000/381) | 1.385(54/39) | 1.773(1576/889) | 1.674(241/144) | 2.876(1645/572) | 1.093(153/140) | 1.354(245/181) | 2.114(668/316) | 1.703(797/468) | 1.688(238/141) | 3.370(1567/465) | 1.900(247/130) | 1.593(301/189) | 1.638(552/337) | 2.491(695/279) |
| **67** | 2.552(998/391) | 0.930(40/43) | 1.688(1376/815) | 1.669(197/118) | 2.547(1597/627) | 1.134(178/157) | 1.704(259/152) | 1.822(656/360) | 1.733(740/427) | 1.514(221/146) | 2.731(1439/527) | 1.618(212/131) | 1.762(296/168) | 1.949(530/272) | 2.435(655/269) |
| **68** | 2.608(1119/429) | 1.688(54/32) | 1.719(1482/862) | 1.572(217/138) | 2.833(1768/624) | 1.297(188/145) | 1.527(281/184) | 1.768(672/380) | 1.809(816/451) | 1.550(217/140) | 2.967(1608/542) | 1.695(217/128) | 1.412(329/233) | 1.921(609/317) | 2.773(696/251) |
| **69** | 2.242(1193/532) | 2.103(61/29) | 1.715(1494/871) | 1.399(242/173) | 2.617(1858/710) | 1.329(210/158) | 1.623(271/167) | 1.863(695/373) | 1.495(818/547) | 1.589(240/151) | 2.810(1624/578) | 1.809(246/136) | 1.415(348/246) | 1.842(630/342) | 2.431(717/295) |
| **70** | 2.279(1183/519) | 1.139(41/36) | 1.765(1493/846) | 1.537(189/123) | 2.787(1937/695) | 1.079(177/164) | 1.321(251/190) | 1.724(688/399) | 1.677(795/474) | 1.463(237/162) | 2.812(1665/592) | 2.510(241/96) | 1.673(368/220) | 1.584(572/361) | 2.651(676/255) |
| **71** | 2.230(1200/538) | 1.657(58/35) | 1.730(1472/851) | 1.561(217/139) | 2.614(1835/702) | 1.123(173/154) | 1.608(299/186) | 1.760(718/408) | 1.617(799/494) | 2.052(236/115) | 2.773(1653/596) | 1.828(223/122) | 1.608(341/212) | 1.548(545/352) | 2.713(662/244) |
| **72** | 2.463(1123/456) | 1.581(49/31) | 1.849(1307/707) | 1.571(165/105) | 2.596(1724/664) | 1.440(180/125) | 1.442(248/172) | 1.891(641/339) | 1.788(760/425) | 1.615(176/109) | 2.662(1624/610) | 2.219(233/105) | 1.488(305/205) | 1.764(531/301) | 3.090(618/200) |
| **73** | 2.489(1180/474) | 1.560(39/25) | 1.923(1342/698) | 1.811(201/111) | 2.604(1810/695) | 1.443(176/122) | 1.711(231/135) | 2.058(599/291) | 1.575(729/463) | 1.667(165/99) | 2.808(1581/563) | 1.942(202/104) | 1.576(312/198) | 1.910(594/311) | 2.662(559/210) |
| **74** | 2.503(1209/483) | 2.368(45/19) | 1.935(1333/689) | 1.514(162/107) | 2.906(1799/619) | 1.576(186/118) | 1.635(242/148) | 2.019(646/320) | 1.936(786/406) | 1.275(139/109) | 3.135(1668/532) | 2.026(231/114) | 1.451(328/226) | 1.689(544/322) | 2.958(630/213) |
| **75** | 2.261(1142/505) | 1.259(34/27) | 1.834(1146/625) | 1.604(162/101) | 2.691(1585/589) | 1.164(142/122) | 1.660(234/141) | 2.030(550/271) | 1.611(638/396) | 1.609(148/92) | 2.911(1563/537) | 2.253(196/87) | 1.563(286/183) | 2.008(486/242) | 3.092(535/173) |
| **76** | 2.425(1152/475) | 1.400(35/25) | 1.897(1108/584) | 1.750(140/80) | 2.566(1655/645) | 1.505(167/111) | 1.511(213/141) | 2.160(566/262) | 1.745(656/376) | 1.272(117/92) | 2.821(1512/536) | 2.163(199/92) | 1.663(291/175) | 1.725(457/265) | 2.775(505/182) |
| **77** | 2.358(1106/469) | 1.478(34/23) | 1.788(980/548) | 1.675(139/83) | 2.637(1506/571) | 1.109(122/110) | 1.492(191/128) | 2.147(483/225) | 1.706(616/361) | 1.675(129/77) | 2.915(1379/473) | 2.000(204/102) | 1.472(259/176) | 1.598(422/264) | 2.623(425/162) |
| **78** | 2.527(988/391) | 2.067(31/15) | 1.768(801/453) | 1.806(112/62) | 2.476(1327/536) | 1.117(124/111) | 1.611(174/108) | 1.803(393/218) | 1.698(523/308) | 1.487(113/76) | 2.622(1206/460) | 2.000(156/78) | 1.688(233/138) | 1.733(409/236) | 2.802(367/131) |
| **79** | 2.255(947/420) | 1.043(24/23) | 1.663(805/484) | 1.593(94/59) | 2.433(1343/552) | 1.363(124/91) | 1.590(186/117) | 1.918(374/195) | 1.715(511/298) | 1.462(95/65) | 2.726(1216/446) | 1.885(147/78) | 1.513(233/154) | 1.723(386/224) | 2.430(367/151) |
| **80** | 2.198(954/434) | 1.692(22/13) | 1.808(723/400) | 2.204(108/49) | 2.448(1278/522) | 1.446(120/83) | 1.466(151/103) | 1.973(361/183) | 1.508(448/297) | 1.617(97/60) | 2.519(1141/453) | 2.547(135/53) | 1.408(207/147) | 1.535(413/269) | 2.815(335/119) |
| **80+** | 2.100(8831/4205) | 1.337(139/104) | 1.550(4214/2718) | 1.614(597/370) | 2.179(9811/4503) | 1.217(818/672) | 1.397(1097/785) | 1.684(2252/1337) | 1.409(2851/2024) | 1.362(523/384) | 2.396(9911/4136) | 1.707(1050/615) | 1.210(1476/1220) | 1.625(3852/2370) | 2.181(1987/911) |

**Table S10.** Fertility rate of at different ages and the proportion of newborns to women of each age group relative to the total number of newborns

| Age | Fertility rate (‰) | the proportion of children born relative to the total birth population (%) |
| --- | --- | --- |
| 15 | 0.05 | 0.00 |
| 16 | 0.96 | 0.09 |
| 17 | 2.74 | 0.25 |
| 18 | 5.00 | 0.46 |
| 19 | 5.48 | 0.51 |
| 20 | 11.95 | 1.10 |
| 21 | 25.75 | 2.38 |
| 22 | 36.38 | 3.36 |
| 23 | 53.87 | 4.97 |
| 24 | 53.49 | 4.94 |
| 25 | 68.36 | 6.31 |
| 26 | 88.30 | 8.15 |
| 27 | 95.53 | 8.82 |
| 28 | 98.02 | 9.04 |
| 29 | 88.51 | 8.17 |
| 30 | 65.16 | 6.01 |
| 31 | 70.72 | 6.53 |
| 32 | 65.84 | 6.08 |
| 33 | 48.66 | 4.49 |
| 34 | 46.12 | 4.26 |
| 35 | 36.68 | 3.38 |
| 36 | 27.72 | 2.56 |
| 37 | 24.93 | 2.30 |
| 38 | 17.91 | 1.65 |
| 39 | 12.91 | 1.19 |
| 40 | 11.47 | 1.06 |
| 41 | 6.93 | 0.64 |
| 42 | 5.46 | 0.50 |
| 43 | 3.01 | 0.28 |
| 44 | 2.97 | 0.27 |
| 45 | 1.31 | 0.12 |
| 46 | 0.86 | 0.08 |
| 47 | 0.33 | 0.03 |
| 48 | 0.26 | 0.02 |
| 49 | 0.07 | 0.01 |

**Table S11. The monthly MP resistance rates, the number of MP-positive cases, and the number of MP-resistant cases for each province from July 2023 to May 2024**

| **Province** | **Jul-23** | **Aug-23** | **Sep-23** | **Oct-23** | **Nov-23** | **Dec-23** | **Jan-24** | **Feb-24** | **Mar-24** | **Apr-24** | **May-24** |
| --- | --- | --- | --- | --- | --- | --- | --- | --- | --- | --- | --- |
| Shanghai | 73.790%(183/248) | 84.856%(325/383) | 81.361%(502/617) | 83.596%(744/890) | 82.654%(953/1153) | 81.116%(1018/1255) | 80.817%(851/1053) | 79.839%(495/620) | 73.171%(270/369) | 74.118%(441/595) | 73.449%(296/403) |
| Yunnan | 74.459%(172/231) | 81.435%(193/237) | 77.959%(191/245) | 83.736%(381/455) | 85.945%(746/868) | 89.201%(1049/1176) | 91.362%(1301/1424) | 90.871%(876/964) | 87.781%(625/712) | 89.347%(520/582) | 88.830%(167/188) |
| Neimongol | 68.750%(22/32) | 86.275%(44/51) | 88.636%(78/88) | 94.737%(108/114) | 95.035%(134/141) | 95.570%(151/158) | 95.370%(309/324) | 96.610%(171/177) | 97.268%(178/183) | 97.297%(216/222) | 93.706%(134/143) |
| Beijing | 71.429%(5/7) | 89.474%(17/19) | 91.837%(45/49) | 96.703%(88/91) | 94.265%(263/279) | 96.308%(313/325) | 94.517%(362/383) | 96.667%(145/150) | 92.000%(115/125) | 95.270%(141/148) | 96.522%(111/115) |
| Jilin | 85.366%(35/41) | 90.909%(70/77) | 97.378%(260/267) | 97.826%(180/184) | 97.368%(296/304) | 92.704%(216/233) | 96.557%(617/639) | 89.224%(207/232) | 95.918%(188/196) | 87.273%(192/220) | 93.443%(114/122) |
| Sichuan | 92.382%(667/722) | 91.058%(723/794) | 92.413%(609/659) | 93.712%(1386/1479) | 94.996%(2202/2318) | 95.269%(2658/2790) | 94.115%(2351/2498) | 95.012%(1181/1243) | 92.973%(860/925) | 93.890%(1091/1162) | 95.201%(734/771) |
| Tianjin | 84.951%(175/206) | 89.931%(393/437) | 82.147%(727/885) | 81.927%(893/1090) | 90.000%(135/150) | 85.165%(155/182) | 86.364%(95/110) | 75.000%(60/80) | 79.545%(35/44) | 84.000%(63/75) | 83.654%(87/104) |
| Ningxia | 50.000%(10/20) | 82.759%(24/29) | 88.889%(40/45) | 94.681%(89/94) | 92.143%(129/140) | 97.403%(150/154) | 93.522%(231/247) | 94.545%(156/165) | 91.379%(159/174) | 93.000%(186/200) | 98.276%(114/116) |
| Anhui | 78.801%(736/934) | 78.929%(693/878) | 78.228%(521/666) | 81.745%(815/997) | 78.571%(638/812) | 78.977%(556/704) | 74.495%(406/545) | 74.165%(333/449) | 74.468%(280/376) | 82.812%(371/448) | 79.257%(256/323) |
| Shandong | 94.311%(431/457) | 93.060%(590/634) | 97.297%(1044/1073) | 96.682%(2040/2110) | 94.629%(2255/2383) | 92.376%(1781/1928) | 89.244%(1701/1906) | 88.239%(1523/1726) | 88.239%(1298/1471) | 92.015%(1717/1866) | 91.855%(1466/1596) |
| Shanxi | 80.000%(24/30) | 92.593%(50/54) | 92.683%(38/41) | 94.286%(66/70) | 98.844%(171/173) | 93.671%(222/237) | 95.169%(197/207) | 91.736%(111/121) | 92.701%(127/137) | 98.305%(174/177) | 96.629%(86/89) |
| Guangdong | 86.346%(3478/4028) | 89.431%(3723/4163) | 90.372%(3398/3760) | 92.090%(5495/5967) | 92.412%(6491/7024) | 91.814%(6303/6865) | 91.153%(6254/6861) | 90.707%(3514/3874) | 90.074%(3176/3526) | 91.679%(4407/4807) | 93.018%(2691/2893) |
| Guangxi | 65.230%(651/998) | 75.607%(809/1070) | 79.249%(760/959) | 82.157%(1234/1502) | 79.975%(1282/1603) | 80.505%(1371/1703) | 80.180%(1335/1665) | 78.113%(803/1028) | 77.924%(593/761) | 81.447%(777/954) | 81.818%(414/506) |
| Xinjiang | 72.222%(13/18) | 92.683%(38/41) | 84.211%(48/57) | 92.105%(70/76) | 90.452%(180/199) | 94.798%(164/173) | 94.231%(392/416) | 95.509%(319/334) | 92.655%(328/354) | 90.126%(429/476) | 92.230%(273/296) |
| Jiangsu | 97.621%(1149/1177) | 98.601%(1551/1573) | 97.694%(1398/1431) | 97.473%(2160/2216) | 97.123%(2127/2190) | 96.076%(1812/1886) | 95.627%(1509/1578) | 95.714%(938/980) | 96.763%(807/834) | 97.310%(1266/1301) | 97.535%(831/852) |
| Jiangxi | 72.687%(165/227) | 77.157%(152/197) | 88.660%(172/194) | 86.087%(198/230) | 88.772%(253/285) | 86.496%(237/274) | 91.401%(287/314) | 93.774%(241/257) | 91.520%(313/342) | 90.830%(525/578) | 92.195%(378/410) |
| Hebei | 80.392%(82/102) | 91.736%(111/121) | 92.233%(95/103) | 91.391%(138/151) | 94.558%(139/147) | 93.431%(128/137) | 87.166%(163/187) | 87.952%(73/83) | 86.111%(62/72) | 92.708%(89/96) | 85.333%(64/75) |
| Henan | 74.520%(427/573) | 92.125%(620/673) | 93.528%(867/927) | 92.635%(1849/1996) | 92.881%(1983/2135) | 94.016%(1524/1621) | 91.387%(1390/1521) | 92.654%(946/1021) | 93.217%(852/914) | 92.281%(1315/1425) | 94.118%(1024/1088) |
| Zhejiang | 84.226%(582/691) | 95.441%(628/658) | 95.833%(575/600) | 95.652%(880/920) | 95.616%(916/958) | 94.751%(686/724) | 91.667%(605/660) | 94.175%(388/412) | 91.927%(501/545) | 93.597%(497/531) | 94.885%(371/391) |
| Hainan | 91.598%(447/488) | 94.574%(488/516) | 93.548%(435/465) | 93.054%(777/835) | 95.511%(1681/1760) | 95.220%(2251/2364) | 94.602%(2366/2501) | 95.225%(1416/1487) | 94.254%(935/992) | 94.298%(860/912) | 94.725%(413/436) |
| Hubei | 82.789%(2713/3277) | 84.378%(3003/3559) | 83.547%(2341/2802) | 87.520%(3766/4303) | 91.701%(3768/4109) | 84.620%(2872/3394) | 90.355%(2492/2758) | 89.323%(1280/1433) | 86.657%(1143/1319) | 91.782%(1664/1813) | 90.177%(1120/1242) |
| Hunan | 75.484%(1053/1395) | 82.168%(1304/1587) | 88.336%(977/1106) | 90.032%(1689/1876) | 88.652%(2039/2300) | 88.464%(2362/2670) | 87.336%(1993/2282) | 87.278%(1276/1462) | 85.868%(1197/1394) | 90.941%(2098/2307) | 92.836%(1555/1675) |
| Gansu | 81.395%(35/43) | 81.538%(53/65) | 93.548%(58/62) | 87.755%(43/49) | 95.804%(137/143) | 95.495%(106/111) | 95.031%(153/161) | 88.793%(103/116) | 96.951%(159/164) | 97.107%(235/242) | 96.454%(136/141) |
| Fujian | 91.938%(650/707) | 91.954%(560/609) | 90.222%(406/450) | 90.937%(602/662) | 91.255%(720/789) | 88.326%(628/711) | 91.236%(760/833) | 87.421%(417/477) | 87.393%(305/349) | 90.137%(329/365) | 89.815%(194/216) |
| Guizhou | 76.129%(118/155) | 73.762%(149/202) | 86.486%(192/222) | 87.917%(211/240) | 82.867%(237/286) | 83.200%(312/375) | 87.136%(359/412) | 83.209%(223/268) | 82.759%(168/203) | 81.752%(224/274) | 83.902%(172/205) |
| Liaoning | 99.174%(120/121) | 97.867%(367/375) | 98.699%(683/692) | 97.248%(1166/1199) | 97.502%(1171/1201) | 96.288%(856/889) | 95.117%(448/471) | 91.781%(134/146) | 92.857%(104/112) | 94.318%(83/88) | 98.333%(59/60) |
| Chongqing | 92.767%(513/553) | 88.667%(399/450) | 82.095%(243/296) | 90.483%(599/662) | 91.639%(1085/1184) | 93.110%(1581/1698) | 93.817%(1396/1488) | 91.613%(710/775) | 87.520%(561/641) | 95.432%(773/810) | 96.970%(448/462) |
| Shaanxi | 78.594%(246/313) | 95.274%(504/529) | 97.289%(610/627) | 98.305%(1450/1475) | 97.197%(2289/2355) | 94.960%(1300/1369) | 93.722%(627/669) | 97.083%(233/240) | 92.952%(211/227) | 91.630%(208/227) | 94.309%(116/123) |
| Heilongjiang | 70.909%(39/55) | 89.011%(81/91) | 96.089%(172/179) | 95.551%(494/517) | 96.081%(809/842) | 95.913%(1009/1052) | 92.577%(873/943) | 88.068%(310/352) | 89.177%(206/231) | 91.964%(309/336) | 89.908%(294/327) |

**Table S12.Metadata information for all MP genomes utilized in this study, encompassing data sources, isolation locations, isolation date, lineages, P1 gene typing, and MR mutation details.**

| **IDs** | **Strains** | **Source** | **Region** | **Years** | **Lineage** | **p1 gene typing** | **MR mutation** |
| --- | --- | --- | --- | --- | --- | --- | --- |
| GCF_000027345.1 | M129 | NCBI | - | - | T1-1 | 1 | - |
| GCF_002095995.1 | S4 | NCBI | China | 2016 | T1-1 | 1 | A2063G |
| GCF_002096035.1 | S12 | NCBI | China | 2016 | T1-1 | 1 | A2063G |
| GCF_000331085.3 | M129-B7 | NCBI | USA | - | T1-1 | 1 | - |
| GCF_001296615.1 | Mympn_4802 | NCBI | Tunisia | 2006 | T1-1 | 1 | - |
| GCF_001296855.1 | Mympn_4807 | NCBI | Tunisia | 2008 | T1-1 | 1 | - |
| GCF_002090215.1 | S63 | NCBI | China | 2016 | T1-1 | 1 | A2063G |
| GCF_002090235.1 | FH | NCBI | China | 2016 | T1-1 | 1 | - |
| GCF_002090275.1 | S68 | NCBI | China | 2015 | T1-1 | 1 | A2063G |
| GCF_002090295.1 | S55 | NCBI | China | 2015 | T1-1 | 1 | A2063G |
| GCF_002090315.1 | S91 | NCBI | China | 2016 | T1-1 | 1 | A2063G |
| GCF_002096015.1 | S34 | NCBI | China | 2016 | T1-1 | 1 | A2063G |
| GCF_002128025.1 | 549 | NCBI | USA | 1965 | T1-1 | 1 | - |
| GCF_002147855.1 | M129 | NCBI | USA | 1968 | T1-1 | 1 | - |
| GCF_002563495.1 | 303 | NCBI | USA | 1991 | T1-1 | 1 | - |
| GCF_009947205.1 | 10-1110 | NCBI | South Korea | 2010 | T1-1 | 1 | - |
| GCF_009948395.1 | 10-980 | NCBI | South Korea | 2010 | T1-1 | 1 | - |
| GCF_910574535.1 | - | NCBI | - | - | T1-1 | 1 | - |
| ERR974262 | - | NCBI | - | 1986 | T1-1 | 1 | - |
| GCA_009810075.1 | KPI-119 | NCBI | Japan | 1988 | T1-2 | 1 | - |
| GCF_009810775.1 | P24 | NCBI | Japan | 1985 | T1-2 | 1 | - |
| GCF_009945165.1 | 11-129 | NCBI | South Korea | 2011 | T1-2 | 1 | - |
| GCF_009947575.1 | 10-1059 | NCBI | South Korea | 2010 | T1-2 | 1 | - |
| GCF_030159375.1 | Y12-4 | NCBI | Japan | 2020 | T1-2 | 1 | - |
| GCF_030159435.1 | OA-57 | NCBI | Japan | 2020 | T1-2 | 1 | - |
| GCF_030923185.1 | S66 | NCBI | China:Taiwan | 2019 | T1-2 | 1 | - |
| GCF_030923245.1 | S57 | NCBI | China:Taiwan | 2019 | T1-2 | 1 | A2063T |
| GCF_030923315.1 | S52 | NCBI | China:Taiwan | 2019 | T1-2 | 1 | A2063G |
| GCF_030923335.1 | S48 | NCBI | China:Taiwan | 2019 | T1-2 | 1 | - |
| GCF_030923435.1 | S08 | NCBI | China:Taiwan | 2018 | T1-2 | 1 | - |
| GCF_030923475.1 | S09 | NCBI | China:Taiwan | 2018 | T1-2 | 1 | - |
| GCF_030923585.1 | NS14 | NCBI | China:Taiwan | 2019 | T1-2 | 1 | - |
| GCF_030923675.1 | NS03 | NCBI | China:Taiwan | 2019 | T1-2 | 1 | A2063T |
| GCF_030923885.1 | 407-37 | NCBI | China:Taiwan | 2019 | T1-2 | 1 | A2063T |
| GCF_030924095.1 | 407-24 | NCBI | China:Taiwan | 2019 | T1-2 | 1 | A2063T |
| GCF_030924105.1 | 407-27 | NCBI | China:Taiwan | 2019 | T1-2 | 1 | - |
| GCF_030924205.1 | 407-12 | NCBI | China:Taiwan | 2019 | T1-2 | 1 | A2063T |
| GCF_030924235.1 | 403-95 | NCBI | China:Taiwan | 2020 | T1-2 | 1 | A2063T |
| GCF_030924275.1 | 403-87 | NCBI | China:Taiwan | 2020 | T1-2 | 1 | A2063T |
| GCF_030924375.1 | 403-72 | NCBI | China:Taiwan | 2019 | T1-2 | 1 | A2063G |
| GCF_030924435.1 | 403-65 | NCBI | China:Taiwan | 2019 | T1-2 | 1 | A2063T |
| GCF_030924495.1 | 403-36 | NCBI | China:Taiwan | 2019 | T1-2 | 1 | A2063G |
| GCF_030924535.1 | 403-24 | NCBI | China:Taiwan | 2019 | T1-2 | 1 | A2063G |
| GCF_030924575.1 | 403-19 | NCBI | China:Taiwan | 2019 | T1-2 | 1 | A2063G |
| GCF_030924695.1 | 006-426 | NCBI | China:Taiwan | 2019 | T1-2 | 1 | A2063G |
| GCF_030924705.1 | 006-428 | NCBI | China:Taiwan | 2019 | T1-2 | 1 | A2063T |
| GCF_030924915.1 | 006-352 | NCBI | China:Taiwan | 2019 | T1-2 | 1 | - |
| GCF_030924945.1 | 006-350 | NCBI | China:Taiwan | 2019 | T1-2 | 1 | A2063G |
| GCF_030925015.1 | 006-337 | NCBI | China:Taiwan | 2019 | T1-2 | 1 | A2063T |
| GCF_030925085.1 | 006-326 | NCBI | China:Taiwan | 2018 | T1-2 | 1 | A2063G |
| GCF_030925115.1 | 006-210 | NCBI | China:Taiwan | 2018 | T1-2 | 1 | A2063G |
| GCF_030925135.1 | 006-227 | NCBI | China:Taiwan | 2018 | T1-2 | 1 | A2063G |
| GCF_030925155.1 | 006-252 | NCBI | China:Taiwan | 2018 | T1-2 | 1 | A2063G |
| GCF_030925165.1 | 006-226 | NCBI | China:Taiwan | 2018 | T1-2 | 1 | A2063G |
| GCF_030925235.1 | 673 | NCBI | China:Taiwan | 2019 | T1-2 | 1 | A2063G |
| GCF_030925255.1 | 676 | NCBI | China:Taiwan | 2020 | T1-2 | 1 | A2063G |
| SRR11193106 | CGUS66 | NCBI | China:Taiwan | 2019 | T1-2 | 1 | - |
| SRR11193107 | CGUS57 | NCBI | China:Taiwan | 2019 | T1-2 | 1 | A2063T |
| SRR11193111 | CGUS52 | NCBI | China:Taiwan | 2019 | T1-2 | 1 | A2063G |
| SRR11193113 | CGUS48 | NCBI | China:Taiwan | 2018 | T1-2 | 1 | - |
| SRR11193115 | CGUNS03 | NCBI | China:Taiwan | 2019 | T1-2 | 1 | A2063T |
| SRR11193123 | CGU407-024 | NCBI | China:Taiwan | 2019 | T1-2 | 1 | A2063T |
| SRR11193127 | CGU006-426-A | NCBI | China:Taiwan | 2019 | T1-2 | 1 | A2063G |
| SRR11193131 | CGU006-350-A | NCBI | China:Taiwan | 2019 | T1-2 | 1 | A2063G |
| SRR11193133 | CGU006-210-A | NCBI | China:Taiwan | 2018 | T1-2 | 1 | A2063G |
| SRR12190407 | CGU006-352-A | NCBI | China:Taiwan | 2019 | T1-2 | 1 | - |
| SRR12190409 | CGU006-252-A | NCBI | China:Taiwan | 2018 | T1-2 | 1 | A2063G |
| SRR12190413 | CGUS09 | NCBI | China:Taiwan | 2018 | T1-2 | 1 | - |
| SRR12190414 | CGUS08 | NCBI | China:Taiwan | 2018 | T1-2 | 1 | - |
| SRR12190415 | CGU006-227-A | NCBI | China:Taiwan | 2018 | T1-2 | 1 | A2063G |
| SRR12190416 | CGU006-226-A | NCBI | China:Taiwan | 2018 | T1-2 | 1 | A2063G |
| SRR3924626 | NM1 | NCBI | USA | 2010 | T1-2 | 1 | - |
| SRR3924637 | EPC83 | NCBI | USA | 2011 | T1-2 | 1 | - |
| SRR3924641 | EPC104 | NCBI | USA | 2012 | T1-2 | 1 | - |
| SRR3924643 | EPC122 | NCBI | USA | 2012 | T1-2 | 1 | - |
| GCF_001455795.1 | CIP12265 | NCBI | - | 2012 | T1-3 | 1 | A2063G |
| GCF_009810295.1 | KCH-144_S | NCBI | Japan | 2008 | T1-3 | 1 | - |
| GCF_009810595.1 | KCH-120 | NCBI | Japan | 2008 | T1-3 | 1 | - |
| GCF_009810915.1 | KCH-443 | NCBI | Japan | 2011 | T1-3 | 1 | A2063G |
| GCF_009811135.1 | Y3-8 | NCBI | Japan | 2011 | T1-3 | 1 | A2063G |
| GCF_009810355.1 | KPI-040 | NCBI | Japan | 1983 | T1-3 | 1 | - |
| GCF_000319675.2 | PI | NCBI | USA | - | T1-3 | 1 | - |
| GCF_001272735.1 | 51494 | NCBI | USA | 2006 | T1-3 | 1 | - |
| GCF_001272755.1 | 54089 | NCBI | USA | 2009 | T1-3 | 1 | A2063G |
| GCF_001272775.1 | 54524 | NCBI | USA | 2009 | T1-3 | 1 | - |
| GCF_001272795.1 | 85084 | NCBI | China | 1985 | T1-3 | 1 | - |
| GCF_001272815.1 | 85138 | NCBI | China | 1985 | T1-3 | 1 | - |
| GCF_001296485.1 | Mympn_1145 | NCBI | France | 1999 | T1-3 | 1 | - |
| GCF_001296505.1 | Mympn_2285 | NCBI | France | 1996 | T1-3 | 1 | - |
| GCF_001296585.1 | Mympn_4010 | NCBI | France | 2005 | T1-3 | 1 | - |
| GCF_001296665.1 | Mympn_5392 | NCBI | Germany | 1993 | T1-3 | 1 | - |
| GCF_001296705.1 | Mympn_5767 | NCBI | France | 2011 | T1-3 | 1 | - |
| GCF_001296725.1 | Mympn_5817 | NCBI | France | 2011 | T1-3 | 1 | - |
| GCF_001296815.1 | Mympn_3912 | NCBI | France | 2005 | T1-3 | 1 | - |
| GCF_001296885.1 | Mympn_6250 | NCBI | France | 2011 | T1-3 | 1 | - |
| GCF_001296895.1 | Mympn_5837 | NCBI | France | 2011 | T1-3 | 1 | - |
| GCF_001296905.1 | Mympn_6421 | NCBI | France | 1979 | T1-3 | 1 | - |
| GCF_002128045.1 | FL8 | NCBI | USA | 2012 | T1-3 | 1 | - |
| GCF_002128065.1 | E16 | NCBI | Egypt | 2010 | T1-3 | 1 | - |
| GCF_002128145.1 | FL1 | NCBI | USA | 2012 | T1-3 | 1 | - |
| GCF_002563345.1 | CO36 | NCBI | USA | 2013 | T1-3 | 1 | - |
| GCF_002563415.1 | 986 | NCBI | Kenya | 1998 | T1-3 | 1 | - |
| GCF_002563435.1 | K21 | NCBI | Kenya | 2010 | T1-3 | 1 | - |
| GCF_002563515.1 | G6 | NCBI | Guatemala | 2010 | T1-3 | 1 | - |
| GCF_009810095.1 | KPI-136 | NCBI | Japan | 1989 | T1-3 | 1 | - |
| GCF_009810115.1 | KPI-180 | NCBI | Japan | 1992 | T1-3 | 1 | - |
| GCF_009810235.1 | K-001 | NCBI | Japan | 2011 | T1-3 | 1 | A2063T |
| GCF_009810395.1 | KPI-079 | NCBI | Japan | 1985 | T1-3 | 1 | - |
| GCF_009810435.1 | KPI-111 | NCBI | Japan | 1988 | T1-3 | 1 | - |
| GCF_009810455.1 | KPI-131 | NCBI | Japan | 1988 | T1-3 | 1 | - |
| GCF_009810695.1 | K-12 | NCBI | Japan | 2012 | T1-3 | 1 | A2063T |
| GCF_009810755.1 | K-35 | NCBI | Japan | 2012 | T1-3 | 1 | A2063T |
| GCF_009810975.1 | KCH-721 | NCBI | Japan | 2011 | T1-3 | 1 | - |
| GCF_009811255.1 | Y4-35 | NCBI | Japan | 2011 | T1-3 | 1 | - |
| GCF_009943205.1 | 11-994 | NCBI | South Korea | 2011 | T1-3 | 1 | - |
| GCF_009944075.1 | 11-473 | NCBI | South Korea | 2011 | T1-3 | 1 | - |
| GCF_009945535.1 | 11-107 | NCBI | South Korea | 2011 | T1-3 | 1 | - |
| GCF_009946285.1 | 10-1257 | NCBI | South Korea | 2010 | T1-3 | 1 | - |
| GCF_001296735.1 | Mympn_5954 | NCBI | France | 2011 | T1-3 | 1 | A2063G |
| GCF_002127985.1 | 685 | NCBI | Denmark | 1988 | T1-3 | 1 | A2063G |
| DRR040043 | KCH-053 | NCBI | Japan | 2008 | T1-3 | 1 | A2063G |
| DRR040044 | KCH-076 | NCBI | Japan | 2008 | T1-3 | 1 | A2063G |
| DRR040045 | KCH-100 | NCBI | Japan | 2008 | T1-3 | 1 | - |
| DRR040046 | KCH-144 | NCBI | Japan | 2008 | T1-3 | 1 | - |
| DRR040047 | KCH-207 | NCBI | Japan | 2009 | T1-3 | 1 | A2063G |
| DRR040048 | KCH-260 | NCBI | Japan | 2010 | T1-3 | 1 | A2063G |
| DRR040050 | KCH-320 | NCBI | Japan | 2011 | T1-3 | 1 | - |
| DRR040051 | KCH-334 | NCBI | Japan | 2011 | T1-3 | 1 | A2063G |
| DRR040052 | KCH-347 | NCBI | Japan | 2011 | T1-3 | 1 | A2063G |
| DRR040053 | KCH-353 | NCBI | Japan | 2011 | T1-3 | 1 | - |
| DRR040055 | KPI-001 | NCBI | Japan | 1976 | T1-3 | 1 | - |
| DRR040056 | KPI-053 | NCBI | Japan | 1984 | T1-3 | 1 | - |
| DRR040057 | KPI-092 | NCBI | Japan | 1986 | T1-3 | 1 | - |
| DRR040058 | KPI-100 | NCBI | Japan | 1987 | T1-3 | 1 | - |
| ERR974265 | - | NCBI | - | 1982 | T1-3 | 1 | - |
| ERR974266 | - | NCBI | - | 1967 | T1-3 | 1 | - |
| ERR974267 | - | NCBI | - | 1996 | T1-3 | 1 | - |
| ERR974268 | - | NCBI | - | 1968 | T1-3 | 1 | - |
| ERR974269 | - | NCBI | - | 1983 | T1-3 | 1 | - |
| ERR974273 | - | NCBI | - | 1983 | T1-3 | 1 | - |
| ERR974276 | - | NCBI | - | 1982 | T1-3 | 1 | - |
| ERR974277 | - | NCBI | - | 1982 | T1-3 | 1 | - |
| ERR974278 | - | NCBI | - | 1982 | T1-3 | 1 | - |
| ERR974283 | - | NCBI | - | 1983 | T1-3 | 1 | - |
| ERR974288 | - | NCBI | - | 1982 | T1-3 | 1 | - |
| ERR974294 | - | NCBI | - | 1982 | T1-3 | 1 | - |
| SRR3924583 | CO37 | NCBI | USA | 2013 | T1-3 | 1 | - |
| SRR3924584 | OR1 | NCBI | USA | 2011 | T1-3 | 1 | A2063G |
| SRR3924586 | WV9 | NCBI | USA | 2012 | T1-3 | 1 | A2063G |
| SRR3924594 | MA1 | NCBI | USA | 2011 | T1-3 | 1 | - |
| SRR3924615 | O-360 | NCBI | USA | 2007 | T1-3 | 1 | - |
| SRR3924618 | 2P | NCBI | USA | 2007 | T1-3 | 1 | - |
| SRR3924621 | WI3 | NCBI | USA | 2012 | T1-3 | 1 | - |
| SRR3924627 | NM2 | NCBI | USA | 2010 | T1-3 | 1 | A2063G |
| SRR3924630 | CO59 | NCBI | USA | 2013 | T1-3 | 1 | - |
| SRR3924633 | CO58 | NCBI | USA | 2013 | T1-3 | 1 | - |
| SRR3924636 | EPC205 | NCBI | USA | 2012 | T1-3 | 1 | A2063G |
| SRR3924638 | EPC67 | NCBI | USA | 2012 | T1-3 | 1 | A2063G |
| SRR3924639 | 988 | NCBI | Canada | 1992 | T1-3 | 1 | - |
| SRR3924640 | EPC181 | NCBI | USA | 2012 | T1-3 | 1 | - |
| SRR3924642 | EPC37 | NCBI | USA | 2011 | T1-3 | 1 | - |
| SRR3924644 | EPC164 | NCBI | USA | 2012 | T1-3 | 1 | A2063G |
| SRR3924645 | EPC44 | NCBI | USA | 2011 | T1-3 | 1 | - |
| SRR3924646 | EPC230 | NCBI | USA | 2012 | T1-3 | 1 | - |
| SRR3924648 | G10 | NCBI | Guatemala | 2010 | T1-3 | 1 | - |
| SRR3924649 | NM3 | NCBI | USA | 2010 | T1-3 | 1 | A2063G |
| GCF_009810475.1 | KPI-150 | NCBI | Japan | 1990 | T1-3 | 1 | - |
| GCF_009939765.1 | 16-710 | NCBI | South Korea | 2016 | T1-3R | 1 | A2063G |
| GCF_009939785.1 | 16-462 | NCBI | South Korea | 2016 | T1-3R | 1 | A2063G |
| GCF_009939805.1 | 16-118 | NCBI | South Korea | 2016 | T1-3R | 1 | A2063G |
| GCF_009939825.1 | 16-032 | NCBI | South Korea | 2016 | T1-3R | 1 | A2063G |
| GCF_030924755.1 | 006-414 | NCBI | China:Taiwan | 2019 | T1-3R | 1 | A2063G |
| GCF_000733995.1 | M29 | NCBI | China | 2005 | T1-3R | 1 | A2063G |
| GCF_001455605.1 | CIP10349 | NCBI | - | 2010 | T1-3R | 1 | A2063G |
| GCF_001455625.1 | CIP12311 | NCBI | - | 2012 | T1-3R | 1 | A2063G |
| GCF_001455635.1 | CIP10361 | NCBI | - | 2010 | T1-3R | 1 | A2063G |
| GCF_001455675.1 | CIP12206 | NCBI | - | 2012 | T1-3R | 1 | A2063G |
| GCF_001455685.1 | CIP12235 | NCBI | - | 2012 | T1-3R | 1 | A2063G |
| GCF_001455695.1 | CIP12261 | NCBI | - | 2012 | T1-3R | 1 | A2063G |
| GCF_001455735.1 | CIP12267 | NCBI | - | 2012 | T1-3R | 1 | A2063G |
| GCF_001455745.1 | CIP12355 | NCBI | - | 2012 | T1-3R | 1 | A2063G |
| GCF_001455775.1 | CIP12357 | NCBI | - | 2012 | T1-3R | 1 | A2063G |
| GCF_001509195.1 | S355 | NCBI | China | 2012 | T1-3R | 1 | A2063G |
| GCF_001558175.1 | C267 | NCBI | China | 2012 | T1-3R | 1 | A2063G |
| GCF_009810575.1 | SRC-1 | NCBI | Japan | 2013 | T1-3R | 1 | A2063G |
| GCF_009810735.1 | K-32 | NCBI | Japan | 2012 | T1-3R | 1 | A2063G |
| GCF_009810795.1 | KP2440 | NCBI | Japan | 2016 | T1-3R | 1 | A2063G |
| GCF_009810815.1 | KP2446 | NCBI | Japan | 2016 | T1-3R | 1 | A2063G |
| GCF_009810835.1 | KP2450 | NCBI | Japan | 2016 | T1-3R | 1 | A2063G |
| GCF_009810955.1 | KCH-595 | NCBI | Japan | 2011 | T1-3R | 1 | A2063G |
| GCF_009811015.1 | KCH-803 | NCBI | Japan | 2011 | T1-3R | 1 | A2063G |
| GCF_009811055.1 | KCH-837 | NCBI | Japan | 2011 | T1-3R | 1 | A2063G |
| GCF_009811095.1 | KCH-917 | NCBI | Japan | 2011 | T1-3R | 1 | A2063G |
| GCF_009811275.1 | Y4-45 | NCBI | Japan | 2011 | T1-3R | 1 | A2063G |
| GCF_009939845.1 | 16-004 | NCBI | South Korea | 2016 | T1-3R | 1 | A2063G |
| GCF_009939975.1 | 16-002 | NCBI | South Korea | 2016 | T1-3R | 1 | A2063G |
| GCF_009940325.1 | 15-982 | NCBI | South Korea | 2015 | T1-3R | 1 | A2063G |
| GCF_009940965.1 | 15-969 | NCBI | South Korea | 2015 | T1-3R | 1 | A2063G |
| GCF_009941325.1 | 15-885 | NCBI | South Korea | 2015 | T1-3R | 1 | A2063G |
| GCF_009941705.1 | 15-215 | NCBI | South Korea | 2015 | T1-3R | 1 | A2063G |
| GCF_009942395.1 | 12-091 | NCBI | South Korea | 2012 | T1-3R | 1 | A2063G |
| GCF_009942655.1 | 12-060 | NCBI | South Korea | 2012 | T1-3R | 1 | A2063G |
| GCF_009943805.1 | 11-634 | NCBI | South Korea | 2011 | T1-3R | 1 | A2063G |
| GCF_009944335.1 | 11-212 | NCBI | South Korea | 2011 | T1-3R | 1 | A2063G |
| GCF_009946845.1 | 10-1213 | NCBI | South Korea | 2010 | T1-3R | 1 | A2063G |
| GCF_009947985.1 | 10-1048 | NCBI | South Korea | 2010 | T1-3R | 1 | A2063G |
| GCF_030159455.1 | OA-63 | NCBI | Japan | 2020 | T1-3R | 1 | A2063G |
| GCF_030923165.1 | S55 | NCBI | China:Taiwan | 2019 | T1-3R | 1 | A2063G |
| GCF_030923225.1 | S59 | NCBI | China:Taiwan | 2019 | T1-3R | 1 | A2063G |
| GCF_030923285.1 | S53 | NCBI | China:Taiwan | 2019 | T1-3R | 1 | A2063G |
| GCF_030923305.1 | S54 | NCBI | China:Taiwan | 2019 | T1-3R | 1 | A2063G |
| GCF_030923325.1 | S50 | NCBI | China:Taiwan | 2019 | T1-3R | 1 | A2063G |
| GCF_030923395.1 | S45 | NCBI | China:Taiwan | 2019 | T1-3R | 1 | A2063G |
| GCF_030923415.1 | S32 | NCBI | China:Taiwan | 2019 | T1-3R | 1 | A2063G |
| GCF_030923495.1 | S06 | NCBI | China:Taiwan | 2018 | T1-3R | 1 | A2063G |
| GCF_030923515.1 | S04 | NCBI | China:Taiwan | 2018 | T1-3R | 1 | A2063G |
| GCF_030923535.1 | NS17 | NCBI | China:Taiwan | 2019 | T1-3R | 1 | A2063G |
| GCF_030923555.1 | NS02 | NCBI | China:Taiwan | 2019 | T1-3R | 1 | A2063G |
| GCF_030923575.1 | K32 | NCBI | China:Taiwan | 2019 | T1-3R | 1 | A2063G |
| GCF_030923695.1 | NS04 | NCBI | China:Taiwan | 2019 | T1-3R | 1 | A2063G |
| GCF_030923755.1 | K25 | NCBI | China:Taiwan | 2019 | T1-3R | 1 | A2063G |
| GCF_030923795.1 | K06 | NCBI | China:Taiwan | 2019 | T1-3R | 1 | A2063G |
| GCF_030923825.1 | K04 | NCBI | China:Taiwan | 2019 | T1-3R | 1 | A2063G |
| GCF_030923835.1 | K01 | NCBI | China:Taiwan | 2019 | T1-3R | 1 | A2063G |
| GCF_030923875.1 | K08 | NCBI | China:Taiwan | 2019 | T1-3R | 1 | A2063G |
| GCF_030924015.1 | 407-29 | NCBI | China:Taiwan | 2019 | T1-3R | 1 | A2063G |
| GCF_030924025.1 | 407-33 | NCBI | China:Taiwan | 2019 | T1-3R | 1 | A2063G |
| GCF_030924065.1 | 407-28 | NCBI | China:Taiwan | 2019 | T1-3R | 1 | A2063G |
| GCF_030924135.1 | 407-14 | NCBI | China:Taiwan | 2019 | T1-3R | 1 | A2063G |
| GCF_030924155.1 | 403-111 | NCBI | China:Taiwan | 2020 | T1-3R | 1 | A2063G |
| GCF_030924175.1 | 407-21 | NCBI | China:Taiwan | 2019 | T1-3R | 1 | A2063G |
| GCF_030924195.1 | 407-07 | NCBI | China:Taiwan | 2019 | T1-3R | 1 | A2063G |
| GCF_030924255.1 | 403-104 | NCBI | China:Taiwan | 2020 | T1-3R | 1 | A2063G |
| GCF_030924265.1 | 403-103 | NCBI | China:Taiwan | 2020 | T1-3R | 1 | A2063G |
| GCF_030924335.1 | 403-85 | NCBI | China:Taiwan | 2019 | T1-3R | 1 | A2063G |
| GCF_030924355.1 | 403-83 | NCBI | China:Taiwan | 2019 | T1-3R | 1 | A2063G |
| GCF_030924395.1 | 403-61 | NCBI | China:Taiwan | 2019 | T1-3R | 1 | A2063G |
| GCF_030924405.1 | 403-74 | NCBI | China:Taiwan | 2019 | T1-3R | 1 | A2063G |
| GCF_030924455.1 | 403-56 | NCBI | China:Taiwan | 2019 | T1-3R | 1 | A2063G |
| GCF_030924475.1 | 403-50 | NCBI | China:Taiwan | 2019 | T1-3R | 1 | A2063G |
| GCF_030924505.1 | 403-31 | NCBI | China:Taiwan | 2019 | T1-3R | 1 | A2063G |
| GCF_030924595.1 | 403-12 | NCBI | China:Taiwan | 2019 | T1-3R | 1 | A2063G |
| GCF_030924615.1 | 403-11 | NCBI | China:Taiwan | 2019 | T1-3R | 1 | A2063G |
| GCF_030924635.1 | 403-09 | NCBI | China:Taiwan | 2019 | T1-3R | 1 | A2063G |
| GCF_030924675.1 | 403-10 | NCBI | China:Taiwan | 2019 | T1-3R | 1 | A2063G |
| GCF_030924715.1 | 006-425 | NCBI | China:Taiwan | 2019 | T1-3R | 1 | A2063G |
| GCF_030924775.1 | 006-411 | NCBI | China:Taiwan | 2019 | T1-3R | 1 | A2063G |
| GCF_030924795.1 | 006-400 | NCBI | China:Taiwan | 2019 | T1-3R | 1 | A2063G |
| GCF_030924815.1 | 006-390 | NCBI | China:Taiwan | 2019 | T1-3R | 1 | A2063G |
| GCF_030924825.1 | 006-391 | NCBI | China:Taiwan | 2019 | T1-3R | 1 | A2063G |
| GCF_030924875.1 | 006-370 | NCBI | China:Taiwan | 2019 | T1-3R | 1 | A2063G |
| GCF_030924895.1 | 006-361 | NCBI | China:Taiwan | 2019 | T1-3R | 1 | A2063G |
| GCF_030924935.1 | 006-356 | NCBI | China:Taiwan | 2019 | T1-3R | 1 | A2063G |
| GCF_030924995.1 | 006-342 | NCBI | China:Taiwan | 2019 | T1-3R | 1 | A2063G |
| GCF_030925035.1 | 006-324 | NCBI | China:Taiwan | 2018 | T1-3R | 1 | A2063G |
| GCF_030925055.1 | 006-312 | NCBI | China:Taiwan | 2018 | T1-3R | 1 | A2063G |
| GCF_030925175.1 | 006-213 | NCBI | China:Taiwan | 2018 | T1-3R | 1 | A2063G |
| GCF_030925215.1 | 677 | NCBI | China:Taiwan | 2020 | T1-3R | 1 | A2063G |
| GCF_030925245.1 | 639 | NCBI | China:Taiwan | 2019 | T1-3R | 1 | A2063G |
| GCF_030925295.1 | 663 | NCBI | China:Taiwan | 2019 | T1-3R | 1 | A2063G |
| GCF_030925315.1 | 637 | NCBI | China:Taiwan | 2019 | T1-3R | 1 | A2063G |
| GCF_030925335.1 | 627 | NCBI | China:Taiwan | 2019 | T1-3R | 1 | A2063G |
| GCF_030925355.1 | 625 | NCBI | China:Taiwan | 2019 | T1-3R | 1 | A2063G |
| GCF_030925375.1 | 633 | NCBI | China:Taiwan | 2019 | T1-3R | 1 | A2063G |
| GCF_030925385.1 | 630 | NCBI | China:Taiwan | 2019 | T1-3R | 1 | A2063G |
| GCF_030928045.1 | NS01 | NCBI | China:Taiwan | 2019 | T1-3R | 1 | A2063G |
| CP141298 | CN2023-A40 | in this study | China | 2023 | T1-3R | 1 | A2063G |
| CP141299 | CN2023-A41 | in this study | China | 2023 | T1-3R | 1 | A2063G |
| DRR040054 | KCH-365 | NCBI | Japan | 2012 | T1-3R | 1 | A2063G |
| SRR11193108 | CGU633 | NCBI | China:Taiwan | 2019 | T1-3R | 1 | A2063G |
| SRR11193109 | CGUS55 | NCBI | China:Taiwan | 2019 | T1-3R | 1 | A2063G |
| SRR11193110 | CGUS53 | NCBI | China:Taiwan | 2019 | T1-3R | 1 | A2063G |
| SRR11193112 | CGUS50 | NCBI | China:Taiwan | 2019 | T1-3R | 1 | A2063G |
| SRR11193116 | CGUNS01 | NCBI | China:Taiwan | 2019 | T1-3R | 1 | A2063G |
| SRR11193117 | CGUK25 | NCBI | China:Taiwan | 2019 | T1-3R | 1 | A2063G |
| SRR11193118 | CGUK08 | NCBI | China:Taiwan | 2019 | T1-3R | 1 | A2063G |
| SRR11193119 | CGU630 | NCBI | China:Taiwan | 2019 | T1-3R | 1 | A2063G |
| SRR11193120 | CGUK06 | NCBI | China:Taiwan | 2019 | T1-3R | 1 | A2063G |
| SRR11193121 | CGUK04 | NCBI | China:Taiwan | 2019 | T1-3R | 1 | A2063G |
| SRR11193122 | CGUK01 | NCBI | China:Taiwan | 2019 | T1-3R | 1 | A2063G |
| SRR11193124 | CGU407-014 | NCBI | China:Taiwan | 2019 | T1-3R | 1 | A2063G |
| SRR11193125 | CGU403-009 | NCBI | China:Taiwan | 2019 | T1-3R | 1 | A2063G |
| SRR11193128 | CGU006-391-A | NCBI | China:Taiwan | 2019 | T1-3R | 1 | A2063G |
| SRR11193129 | CGU006-370-A | NCBI | China:Taiwan | 2019 | T1-3R | 1 | A2063G |
| SRR11193130 | CGU006-356-A | NCBI | China:Taiwan | 2019 | T1-3R | 1 | A2063G |
| SRR11193132 | CGU006-213-A | NCBI | China:Taiwan | 2018 | T1-3R | 1 | A2063G |
| SRR11193134 | CGU006-390-A | NCBI | China:Taiwan | 2019 | T1-3R | 1 | A2063G |
| SRR11193135 | CGU639 | NCBI | China:Taiwan | 2019 | T1-3R | 1 | A2063G |
| SRR11193136 | CGU627 | NCBI | China:Taiwan | 2019 | T1-3R | 1 | A2063G |
| SRR11193137 | CGU625 | NCBI | China:Taiwan | 2019 | T1-3R | 1 | A2063G |
| SRR12190402 | CGUS04 | NCBI | China:Taiwan | 2018 | T1-3R | 1 | A2063G |
| SRR12190403 | CGUNS04 | NCBI | China:Taiwan | 2019 | T1-3R | 1 | A2063G |
| SRR12190404 | CGUNS02 | NCBI | China:Taiwan | 2019 | T1-3R | 1 | A2063G |
| SRR12190405 | CGU407-028 | NCBI | China:Taiwan | 2019 | T1-3R | 1 | A2063G |
| SRR12190406 | CGU403-012 | NCBI | China:Taiwan | 2019 | T1-3R | 1 | A2063G |
| SRR12190408 | CGU006-312-A | NCBI | China:Taiwan | 2018 | T1-3R | 1 | A2063G |
| SRR12190411 | CGUS59 | NCBI | China:Taiwan | 2019 | T1-3R | 1 | A2063G |
| SRR12190412 | CGUS45 | NCBI | China:Taiwan | 2019 | T1-3R | 1 | A2063G |
| GCF_000283755.1 | - | NCBI | - | - | T2-A | 2 | - |
| GCF_001296565.1 | Mympn_3896 | NCBI | France | 2005 | T2-A | 2 | - |
| GCF_001296685.1 | Mympn_5393 | NCBI | Germany | 1991 | T2-A | 2 | - |
| GCF_001296765.1 | Mympn_6009 | NCBI | France | 2011 | T2-A | 2 | - |
| GCF_002128005.1 | E57 | NCBI | Egypt | 2009 | T2-A | 2 | - |
| GCF_002128125.1 | CO3 | NCBI | USA | 2014 | T2-A | 2 | - |
| GCF_002128265.1 | 519 | NCBI | USA | 1995 | T2-A | 2 | - |
| GCF_002355715.1 | KCH-405 | NCBI | Japan | 2012 | T2-A | 2 | - |
| GCF_009810155.1 | KCH-348 | NCBI | Japan | 2011 | T2-A | 2 | - |
| GCF_009810175.1 | I-37 | NCBI | Japan | 2013 | T2-A | 2 | - |
| GCF_009810255.1 | K-004_L | NCBI | Japan | 2011 | T2-A | 2 | - |
| GCF_009810275.1 | K-004_S | NCBI | Japan | 2011 | T2-A | 2 | - |
| GCF_009810495.1 | KPI-165 | NCBI | Japan | 1991 | T2-A | 2 | - |
| GCF_009810515.1 | KPI-170 | NCBI | Japan | 1991 | T2-A | 2 | - |
| GCF_009810535.1 | KPI-199 | NCBI | Japan | 1993 | T2-A | 2 | - |
| GCF_009810615.1 | KCH-161 | NCBI | Japan | 2009 | T2-A | 2 | - |
| GCF_009810635.1 | KCH-266 | NCBI | Japan | 2010 | T2-A | 2 | - |
| GCF_009810655.1 | KCH-338 | NCBI | Japan | 2011 | T2-A | 2 | - |
| GCF_009810675.1 | KCH-349 | NCBI | Japan | 2011 | T2-A | 2 | - |
| GCF_009810715.1 | K-24 | NCBI | Japan | 2012 | T2-A | 2 | - |
| GCF_009810855.1 | M241 | NCBI | Japan | 2011 | T2-A | 2 | - |
| GCF_009810875.1 | M282 | NCBI | Japan | 2011 | T2-A | 2 | - |
| GCF_009810935.1 | KCH-501 | NCBI | Japan | 2011 | T2-A | 2 | - |
| GCF_009810995.1 | KCH-731 | NCBI | Japan | 2011 | T2-A | 2 | A2063G |
| GCF_009811035.1 | KCH-825 | NCBI | Japan | 2011 | T2-A | 2 | - |
| GCF_009811075.1 | KCH-845 | NCBI | Japan | 2011 | T2-A | 2 | - |
| GCF_009811115.1 | Y3-2 | NCBI | Japan | 2011 | T2-A | 2 | - |
| GCF_009811175.1 | Y3-43 | NCBI | Japan | 2011 | T2-A | 2 | A2063G |
| GCF_009811195.1 | Y3-60 | NCBI | Japan | 2011 | T2-A | 2 | - |
| GCF_009939745.1 | 16-734 | NCBI | South Korea | 2016 | T2-A | 2 | - |
| GCF_009942155.1 | 14-637 | NCBI | South Korea | 2014 | T2-A | 2 | - |
| GCF_009942915.1 | 11-1384 | NCBI | South Korea | 2011 | T2-A | 2 | - |
| GCF_009943505.1 | 11-949 | NCBI | South Korea | 2011 | T2-A | 2 | A2063G |
| GCF_009944725.1 | 11-174 | NCBI | South Korea | 2011 | T2-A | 2 | - |
| GCF_009945865.1 | 10-1385 | NCBI | South Korea | 2010 | T2-A | 2 | - |
| GCF_030923175.1 | S62 | NCBI | China:Taiwan | 2019 | T2-A | 2 | - |
| GCF_030923455.1 | S13 | NCBI | China:Taiwan | 2018 | T2-A | 2 | - |
| GCF_030924315.1 | 403-88 | NCBI | China:Taiwan | 2020 | T2-A | 2 | A2063G |
| GCF_030924545.1 | 403-26 | NCBI | China:Taiwan | 2019 | T2-A | 2 | - |
| GCF_030924655.1 | 403-06 | NCBI | China:Taiwan | 2019 | T2-A | 2 | A2063G |
| GCF_030924835.1 | 006-380 | NCBI | China:Taiwan | 2019 | T2-A | 2 | - |
| GCF_030925075.1 | 006-268 | NCBI | China:Taiwan | 2018 | T2-A | 2 | - |
| DRR040049 | KCH-290 | NCBI | Japan | 2011 | T2-A | 2 | - |
| SRR11193114 | CGUS13 | NCBI | China:Taiwan | 2018 | T2-A | 2 | - |
| SRR11193126 | CGU403-006 | NCBI | China:Taiwan | 2019 | T2-A | 2 | A2063G |
| SRR12190410 | CGUS62 | NCBI | China:Taiwan | 2019 | T2-A | 2 | - |
| SRR3924603 | RI2 | NCBI | USA | 2011 | T2-A | 2 | - |
| SRR3924612 | 3076 | NCBI | USA | 2007 | T2-A | 2 | - |
| SRR3924622 | WI6 | NCBI | USA | 2012 | T2-A | 2 | - |
| SRR3924623 | WV1 | NCBI | USA | 2011 | T2-A | 2 | - |
| SRR3924624 | NE4 | NCBI | USA | 2014 | T2-A | 2 | - |
| SRR3924625 | NE26 | NCBI | USA | 2014 | T2-A | 2 | - |
| SRR3924629 | CO13 | NCBI | USA | 2013 | T2-A | 2 | - |
| GCF_000143945.1 | FH | NCBI | - | - | T2-B | 2 | - |
| GCF_000319655.2 | PO1 | NCBI | USA | 1980 | T2-B | 2 | - |
| GCF_000387745.2 | 19294 | NCBI | USA | 1994 | T2-B | 2 | - |
| GCF_001272715.1 | 39443 | NCBI | USA | 1999 | T2-B | 2 | - |
| GCF_001272835.1 | FH | NCBI | USA | 1954 | T2-B | 2 | - |
| GCF_001272855.1 | M1139 | NCBI | UK | 1981 | T2-B | 2 | - |
| GCF_001272875.1 | M2192 | NCBI | UK | 1982 | T2-B | 2 | - |
| GCF_001272895.1 | M2592 | NCBI | UK | 1982 | T2-B | 2 | - |
| GCF_001272915.1 | MAC | NCBI | USA | 1944 | T2-B | 2 | - |
| GCF_001296515.1 | Mympn_3163 | NCBI | France | 2001 | T2-B | 2 | - |
| GCF_001296525.1 | Mympn_2882 | NCBI | Spain | 1999 | T2-B | 2 | - |
| GCF_001296605.1 | Mympn_4318 | NCBI | Japan | 2003 | T2-B | 2 | - |
| GCF_001296625.1 | Mympn_4911 | NCBI | France | 2008 | T2-B | 2 | - |
| GCF_001296785.1 | Mympn_6282 | NCBI | France | 2011 | T2-B | 2 | - |
| GCF_001296805.1 | Mympn_M547 | NCBI | Denmark | 1967 | T2-B | 2 | - |
| GCF_001296825.1 | Mympn_4358 | NCBI | Japan | 2003 | T2-B | 2 | A2063C |
| GCF_001901705.1 | FH | NCBI | USA | 1954 | T2-B | 2 | - |
| GCF_002128085.1 | RI3 | NCBI | USA | 2007 | T2-B | 2 | - |
| GCF_002128105.1 | 1801 | NCBI | USA | 2000 | T2-B | 2 | - |
| GCF_002128165.1 | CO103 | NCBI | USA | 2013 | T2-B | 2 | - |
| GCF_002128185.1 | K27 | NCBI | Kenya | 2010 | T2-B | 2 | - |
| GCF_002128205.1 | 1006 | NCBI | USA | 1999 | T2-B | 2 | A2063G |
| GCF_002128235.1 | 1134 | NCBI | USA | 1999 | T2-B | 2 | - |
| GCF_002128285.1 | GA3 | NCBI | USA | 2012 | T2-B | 2 | - |
| GCF_002355695.1 | KCH-402 | NCBI | Japan | 2012 | T2-B | 2 | - |
| GCF_002563355.1 | 682 | NCBI | Denmark | - | T2-B | 2 | - |
| GCF_002563365.1 | 1005 | NCBI | USA | 1999 | T2-B | 2 | - |
| GCF_002563545.1 | FH | NCBI | USA | 1954 | T2-B | 2 | - |
| GCF_009809995.1 | KPI-002 | NCBI | Japan | 1976 | T2-B | 2 | - |
| GCF_009810015.1 | KPI-020 | NCBI | Japan | 1980 | T2-B | 2 | - |
| GCF_009810035.1 | KPI-037 | NCBI | Japan | 1983 | T2-B | 2 | - |
| GCF_009810055.1 | KPI-064 | NCBI | Japan | 1984 | T2-B | 2 | - |
| GCF_009810135.1 | KPI-187 | NCBI | Japan | 1992 | T2-B | 2 | - |
| GCF_009810195.1 | I-42 | NCBI | Japan | 2013 | T2-B | 2 | - |
| GCF_009810315.1 | KPI-007 | NCBI | Japan | 1979 | T2-B | 2 | - |
| GCF_009810335.1 | KPI-015 | NCBI | Japan | 1980 | T2-B | 2 | - |
| GCF_009810375.1 | KPI-072 | NCBI | Japan | 1985 | T2-B | 2 | - |
| GCF_009810415.1 | KPI-102 | NCBI | Japan | 1987 | T2-B | 2 | - |
| GCF_009810555.1 | KPI-200 | NCBI | Japan | 1994 | T2-B | 2 | - |
| GCF_009810895.1 | K708 | NCBI | Japan | 2011 | T2-B | 2 | - |
| GCF_009811155.1 | Y3-12 | NCBI | Japan | 2011 | T2-B | 2 | - |
| GCF_009811215.1 | Y4-15 | NCBI | Japan | 2011 | T2-B | 2 | - |
| GCF_009811235.1 | Y4-20 | NCBI | Japan | 2011 | T2-B | 2 | A2063G |
| GCF_009811295.1 | Y4-67 | NCBI | Japan | 2011 | T2-B | 2 | - |
| GCF_030159355.1 | KPI-025 | NCBI | Japan | 1980 | T2-B | 2 | - |
| GCF_030159395.1 | Y12-24 | NCBI | Japan | 2020 | T2-B | 2 | A2063G |
| GCF_030159415.1 | Y12-38 | NCBI | Japan | 2020 | T2-B | 2 | - |
| GCF_030924925.1 | 006-345 | NCBI | China:Taiwan | 2019 | T2-B | 2 | - |
| GCF_900660465.1 | NCTC10119 | NCBI | - | 1967 | T2-B | 2 | - |
| ERR974260 | - | NCBI | - | 1981 | T2-B | 2 | - |
| ERR974261 | - | NCBI | - | 1981 | T2-B | 2 | - |
| ERR974263 | - | NCBI | - | 1981 | T2-B | 2 | - |
| ERR974264 | - | NCBI | - | 1978 | T2-B | 2 | - |
| ERR974270 | - | NCBI | - | 1983 | T2-B | 2 | - |
| ERR974271 | - | NCBI | - | 1983 | T2-B | 2 | - |
| ERR974272 | - | NCBI | - | 1982 | T2-B | 2 | - |
| ERR974274 | - | NCBI | - | 1982 | T2-B | 2 | - |
| ERR974275 | - | NCBI | - | 1983 | T2-B | 2 | - |
| ERR974279 | - | NCBI | - | 1983 | T2-B | 2 | - |
| ERR974280 | - | NCBI | - | 1983 | T2-B | 2 | - |
| ERR974281 | - | NCBI | - | 1981 | T2-B | 2 | - |
| ERR974282 | - | NCBI | - | 1981 | T2-B | 2 | - |
| ERR974284 | - | NCBI | - | 1979 | T2-B | 2 | - |
| ERR974285 | - | NCBI | - | 1981 | T2-B | 2 | - |
| ERR974286 | - | NCBI | - | 1982 | T2-B | 2 | - |
| ERR974287 | - | NCBI | - | 1976 | T2-B | 2 | - |
| ERR974289 | - | NCBI | - | 1982 | T2-B | 2 | - |
| ERR974290 | - | NCBI | - | 1982 | T2-B | 2 | - |
| ERR974291 | - | NCBI | - | 1983 | T2-B | 2 | - |
| ERR974292 | - | NCBI | - | 1982 | T2-B | 2 | - |
| ERR974293 | - | NCBI | - | 1983 | T2-B | 2 | - |
| SRR3924595 | SA18 | NCBI | South Africa | 2012 | T2-B | 2 | - |
| SRR3924601 | 987 | NCBI | USA | 1986 | T2-B | 2 | - |
| SRR3924604 | 985 | NCBI | USA | 1988 | T2-B | 2 | - |
| SRR3924607 | 237 | NCBI | USA | 1993 | T2-B | 2 | - |
| SRR3924611 | 334 | NCBI | USA | 1994 | T2-B | 2 | - |
| SRR3924613 | 300 | NCBI | USA | 1994 | T2-B | 2 | - |
| SRR3924614 | 709 | NCBI | USA | 1996 | T2-B | 2 | - |
| SRR3924616 | 399 | NCBI | USA | 1994 | T2-B | 2 | - |
| SRR3924619 | 386 | NCBI | USA | 1994 | T2-B | 2 | - |
| SRR3924620 | 551 | NCBI | USA | 1974 | T2-B | 2 | - |
| SRR3924634 | CO26 | NCBI | USA | 2013 | T2-B | 2 | - |

**Table S13. Statistics of mNGS sequencing for 251 MP-positive samples, along with the clade and resistance profiles of MP in these samples.**

| **Sample IDs** | **clean_reads** | **clean_bases** | **Q20（%）** | **Q30(%)** | **GC(%)** | **Lineage** | **MR mutation** |
| --- | --- | --- | --- | --- | --- | --- | --- |
| I-1 | 96,482,310 | 14,421,985,044 | 96.3847 | 90.7699 | 48.366 | T1-3-R | Undetermined |
| I-108 | 59,672,942 | 8,891,557,809 | 96.3717 | 90.7234 | 42.2961 | T1-3-R | A2063G |
| I-118 | 55,713,132 | 8,285,070,415 | 96.4003 | 90.8624 | 43.8115 | T1-3-R | A2063G |
| I-120 | 82,497,000 | 12,299,357,689 | 96.7153 | 91.4918 | 41.6748 | T1-3-R | A2063G |
| I-123 | 49,680,098 | 7,367,711,428 | 96.1031 | 90.3065 | 42.8528 | T1-3-R | Undetermined |
| I-128 | 63,176,538 | 9,402,158,153 | 95.8839 | 89.9149 | 45.005 | T1-3-R | A2063G |
| I-129 | 78,005,170 | 11,628,137,824 | 94.8457 | 87.9628 | 41.0909 | T1-3-R | A2063G |
| I-131 | 107,971,362 | 16,014,737,791 | 95.8777 | 90.0079 | 42.872 | Undetermined | Undetermined |
| I-135 | 32,351,104 | 4,758,180,291 | 95.5696 | 89.3868 | 51.0461 | Undetermined | Undetermined |
| I-138 | 41,742,568 | 6,205,006,696 | 95.6171 | 89.2674 | 59.0261 | Undetermined | Undetermined |
| I-144 | 23,312,866 | 3,364,725,513 | 94.5563 | 87.618 | 42.5537 | Undetermined | Undetermined |
| I-146 | 59,661,484 | 8,789,872,577 | 94.7703 | 88.0157 | 42.8929 | Undetermined | Undetermined |
| I-15 | 53,761,140 | 8,025,773,794 | 96.5853 | 91.1309 | 41.2449 | T1-3-R | A2063G |
| I-150 | 96,029,864 | 14,346,694,200 | 96.0931 | 90.2164 | 39.9773 | Undetermined | Undetermined |
| I-151 | 112,228,774 | 16,772,953,855 | 97.8747 | 93.8585 | 55.0423 | T1-3-R | Undetermined |
| I-152 | 93,944,964 | 14,020,369,620 | 95.0378 | 88.2111 | 39.5545 | T2-B | Undetermined |
| I-23 | 80,152,956 | 11,936,304,946 | 96.3757 | 90.8782 | 44.2235 | T1-3-R | A2063G |
| I-28 | 78,516,644 | 11,710,172,818 | 96.5673 | 91.1515 | 42.6467 | T2-B | Undetermined |
| I-31 | 59,764,758 | 8,919,473,765 | 96.1085 | 90.262 | 42.4271 | T1-3-R | A2063G |
| I-4 | 68,197,584 | 10,146,487,521 | 96.7255 | 91.4309 | 41.9443 | T1-3-R | Undetermined |
| I-42 | 63,341,346 | 9,471,531,396 | 96.1954 | 90.4272 | 50.7079 | T1-3-R | A2063G |
| I-48 | 64,919,352 | 9,678,572,685 | 96.9995 | 92.0427 | 41.962 | T1-3-R | A2063G |
| I-5 | 71,414,498 | 10,660,246,835 | 96.7635 | 91.5551 | 42.8632 | T1-3-R | Undetermined |
| I-54 | 88,615,334 | 13,231,421,302 | 96.6059 | 91.1745 | 43.4389 | Undetermined | Undetermined |
| I-57 | 73,785,300 | 10,955,353,654 | 96.4051 | 90.877 | 51.0277 | Undetermined | Undetermined |
| I-65 | 43,188,512 | 6,430,305,362 | 96.8113 | 91.6986 | 45.2945 | T1-3-R | A2063G |
| I-67 | 62,513,894 | 9,264,795,984 | 96.0376 | 90.1367 | 43.4077 | T1-2 | Undetermined |
| I-7 | 63,007,468 | 9,324,723,742 | 97.2331 | 92.5793 | 41.7356 | T1-3-R | Undetermined |
| I-79 | 55,992,278 | 8,328,544,040 | 96.7377 | 91.5214 | 45.468 | T1-3-R | A2063G |
| I-82 | 75,816,370 | 11,317,125,696 | 96.2216 | 90.4833 | 41.8594 | Undetermined | Undetermined |
| I-84 | 126,657,252 | 18,931,878,558 | 96.3562 | 90.7484 | 41.2096 | Undetermined | Undetermined |
| I-89 | 53,954,594 | 8,059,065,246 | 96.5864 | 91.1652 | 42.5059 | T2-A | Undetermined |
| I-9 | 91,647,798 | 13,663,027,565 | 97.2783 | 92.6295 | 43.3508 | T1-3-R | Undetermined |
| I-94 | 42,650,010 | 6,343,857,021 | 96.4951 | 91.0909 | 43.6038 | T1-3-R | A2063G |
| I-96 | 55,446,542 | 8,244,227,534 | 95.744 | 89.5327 | 41.8617 | T1-2 | A2063G |
| I-99 | 88,934,506 | 13,271,499,750 | 96.6049 | 91.1842 | 41.568 | T2-A | Undetermined |
| II-112 | 73,072,766 | 10,921,702,271 | 96.542 | 91.1931 | 41.7822 | T1-3-R | Undetermined |
| II-146 | 102,100,116 | 15,264,832,154 | 96.5492 | 91.1939 | 41.6982 | Undetermined | Undetermined |
| II-149 | 112,487,166 | 16,662,315,822 | 95.9422 | 90.3773 | 42.3451 | Undetermined | Undetermined |
| II-151 | 13,103,802 | 1,942,072,458 | 96.2333 | 90.9075 | 42.7873 | Undetermined | Undetermined |
| II-18 | 89,871,112 | 13,432,256,043 | 96.8693 | 91.8369 | 42.4392 | T2-A | A2063G |
| II-19 | 69,020,848 | 10,288,711,238 | 97.5857 | 93.4187 | 41.0157 | T2-B | Undetermined |
| II-24 | 98,804,510 | 14,770,877,319 | 97.1297 | 92.3791 | 41.5491 | Undetermined | Undetermined |
| II-40 | 94,186,964 | 14,079,411,085 | 96.6347 | 91.3969 | 44.2693 | Undetermined | Undetermined |
| II-56 | 76,072,348 | 11,391,105,671 | 96.0031 | 90.043 | 52.5897 | Undetermined | Undetermined |
| II-63 | 84,184,574 | 12,586,492,280 | 96.7851 | 91.649 | 42.456 | T1-3-R | A2063G |
| II-76 | 108,472,510 | 16,242,059,706 | 96.8124 | 91.6629 | 44.8107 | T1-3-R | A2063G |
| II-8 | 97,185,472 | 14,541,233,072 | 97.1823 | 92.4624 | 42.9319 | T1-3-R | A2063G |
| II-85 | 88,942,320 | 13,306,456,134 | 97.2484 | 92.5269 | 42.4496 | T2-B | Undetermined |
| II-86 | 96,170,762 | 14,363,980,627 | 97.1031 | 92.3462 | 42.8376 | T2-B | Undetermined |
| II-87 | 103,342,340 | 15,460,740,659 | 97.2464 | 92.6042 | 42.0293 | T1-3-R | Undetermined |
| III-1 | 55,073,124 | 8,197,141,356 | 97.0669 | 92.2142 | 40.8407 | T1-3-R | A2063G |
| III-100 | 77,324,670 | 11,536,439,276 | 97.2634 | 92.8071 | 59.7342 | T1-3-R | Undetermined |
| III-101 | 45,610,244 | 6,773,618,604 | 96.6211 | 91.3623 | 41.5586 | T1-3-R | Undetermined |
| III-102 | 82,917,136 | 12,345,107,856 | 96.1048 | 90.295 | 41.5587 | T1-3-R | Undetermined |
| III-103 | 77,438,724 | 11,566,696,772 | 97.0887 | 92.1712 | 42.5416 | T1-3-R | Undetermined |
| III-104 | 43,062,836 | 6,279,808,706 | 96.2003 | 90.566 | 42.4266 | Undetermined | Undetermined |
| III-105 | 59,887,256 | 8,856,265,345 | 96.6136 | 91.3253 | 41.9214 | T1-3-R | Undetermined |
| III-106 | 68,404,362 | 10,195,533,328 | 97.228 | 92.5253 | 41.8753 | Undetermined | Undetermined |
| III-107 | 69,835,576 | 10,346,047,177 | 96.5978 | 91.2923 | 41.88 | Undetermined | Undetermined |
| III-108 | 88,751,252 | 13,220,744,467 | 97.214 | 92.5376 | 41.3992 | T1-3-R | A2063G |
| III-109 | 50,626,686 | 7,333,883,693 | 96.5759 | 91.4019 | 43.1958 | Undetermined | Undetermined |
| III-110 | 75,332,562 | 11,148,731,885 | 97.3124 | 92.5492 | 41.0391 | Undetermined | Undetermined |
| III-111 | 69,128,228 | 10,275,791,274 | 97.5956 | 93.3576 | 43.0599 | T1-3-R | A2063G |
| III-112 | 64,413,714 | 9,615,815,879 | 96.7937 | 91.6355 | 41.4556 | T1-3-R | A2063G |
| III-113 | 103,472,604 | 15,434,863,313 | 97.5407 | 93.1851 | 41.1941 | T2-A | A2063G |
| III-114 | 55,266,754 | 8,251,424,322 | 96.8324 | 91.7409 | 40.8388 | T1-3-R | Undetermined |
| III-115 | 79,007,362 | 11,738,161,736 | 97.0201 | 91.8544 | 40.7699 | T1-3-R | A2063G |
| III-116 | 61,297,092 | 9,135,857,417 | 97.4003 | 92.9614 | 41.667 | Undetermined | Undetermined |
| III-117 | 59,861,996 | 8,301,098,651 | 95.7119 | 89.4431 | 45.8452 | T1-3-R | Undetermined |
| III-118 | 67,845,182 | 10,122,989,773 | 97.7206 | 93.5061 | 43.0231 | T1-3-R | Undetermined |
| III-119 | 99,688,966 | 14,861,739,670 | 97.2479 | 92.5936 | 42.6861 | T1-3-R | Undetermined |
| III-120 | 32,674,338 | 4,832,563,662 | 95.8233 | 89.7913 | 41.6555 | Undetermined | Undetermined |
| III-121 | 46,281,068 | 6,888,770,596 | 96.5786 | 91.2327 | 41.7412 | T2-A | Undetermined |
| III-122 | 42,744,716 | 6,273,336,881 | 96.2957 | 90.7353 | 42.2132 | Undetermined | Undetermined |
| III-123 | 68,449,456 | 10,222,991,575 | 96.9972 | 92.026 | 42.432 | Undetermined | Undetermined |
| III-124 | 55,783,494 | 8,325,479,435 | 96.9958 | 91.9746 | 41.1412 | Undetermined | Undetermined |
| III-125 | 74,336,906 | 11,018,010,173 | 97.3684 | 92.8784 | 42.4663 | T1-3-R | Undetermined |
| III-126 | 78,832,454 | 11,729,173,570 | 97.3638 | 92.8476 | 42.7344 | T1-3-R | A2063G |
| III-127 | 58,469,322 | 8,662,070,731 | 97.0175 | 92.0897 | 41.1118 | T1-3-R | A2063G |
| III-128 | 52,421,714 | 7,753,928,805 | 96.2839 | 90.4122 | 42.184 | T1-3-R | Undetermined |
| III-129 | 58,694,846 | 8,670,090,079 | 97.6681 | 93.4495 | 42.3191 | T1-3-R | A2063G |
| III-13 | 61,802,110 | 9,199,604,265 | 97.597 | 93.3172 | 42.6711 | T1-3-R | A2063G |
| III-130 | 82,970,834 | 12,311,912,951 | 96.5789 | 90.9937 | 41.9138 | T1-3-R | Undetermined |
| III-131 | 100,139,052 | 14,935,373,812 | 95.3538 | 88.6844 | 42.0026 | T1-3-R | A2063G |
| III-132 | 55,082,090 | 8,208,184,176 | 96.3522 | 90.5363 | 41.2968 | Undetermined | Undetermined |
| III-133 | 46,723,516 | 6,948,248,376 | 96.9612 | 92.0166 | 41.9567 | T1-3-R | Undetermined |
| III-134 | 58,485,144 | 8,712,412,795 | 96.1394 | 90.1275 | 43.2564 | T1-3-R | Undetermined |
| III-135 | 63,375,500 | 9,455,193,659 | 96.2416 | 90.2974 | 42.1816 | T1-3-R | Undetermined |
| III-136 | 83,274,664 | 12,420,976,868 | 97.2424 | 92.5666 | 42.0306 | Undetermined | Undetermined |
| III-137 | 82,165,062 | 12,219,492,512 | 96.9671 | 92.0212 | 42.4569 | T1-3-R | Undetermined |
| III-138 | 37,947,586 | 5,608,412,424 | 96.7552 | 91.6545 | 42.5875 | T2-A | Undetermined |
| III-139 | 64,503,328 | 9,617,941,166 | 96.1699 | 90.1826 | 42.6658 | Undetermined | Undetermined |
| III-14 | 65,462,422 | 9,769,798,679 | 97.4873 | 93.0236 | 43.6499 | T1-3-R | Undetermined |
| III-140 | 73,804,226 | 11,001,021,756 | 95.9589 | 89.7621 | 41.7205 | Undetermined | Undetermined |
| III-141 | 58,805,778 | 8,773,718,167 | 96.7147 | 91.4215 | 43.4199 | Undetermined | Undetermined |
| III-142 | 24,604,434 | 3,558,638,135 | 95.7456 | 89.4466 | 44.7339 | Undetermined | A2064G |
| III-143 | 55,280,366 | 8,239,198,860 | 96.9714 | 91.9961 | 43.5148 | T1-3-R | Undetermined |
| III-144 | 76,473,418 | 11,391,765,400 | 96.2453 | 90.5952 | 41.6766 | T1-3-R | Undetermined |
| III-145 | 78,026,236 | 11,604,784,157 | 97.1772 | 92.4472 | 42.6912 | Undetermined | Undetermined |
| III-146 | 76,713,026 | 11,389,159,078 | 96.3816 | 90.6505 | 41.1229 | T1-3-R | A2063G |
| III-147 | 56,523,698 | 8,293,135,330 | 95.8606 | 89.6155 | 44.5173 | T2-A | Undetermined |
| III-148 | 59,665,084 | 8,881,380,470 | 96.6447 | 91.111 | 41.8897 | T1-3-R | Undetermined |
| III-149 | 65,148,722 | 9,724,713,457 | 96.3727 | 90.5681 | 41.8992 | T1-3-R | Undetermined |
| III-150 | 35,265,904 | 5,069,002,186 | 95.1427 | 88.3474 | 44.0845 | Undetermined | Undetermined |
| III-151 | 83,423,838 | 12,448,419,356 | 95.8714 | 89.6116 | 41.1937 | Undetermined | A2064G |
| III-152 | 97,190,290 | 14,509,477,757 | 96.4084 | 90.6526 | 41.5489 | T1-3-R | A2063G |
| III-153 | 73,984,968 | 11,043,838,125 | 96.3573 | 90.5341 | 44.1882 | T1-3-R | A2063G |
| III-159 | 69,468,510 | 10,344,727,433 | 97.5787 | 93.2704 | 41.4076 | T2-B | Undetermined |
| III-16 | 55,748,222 | 8,304,115,411 | 97.2674 | 92.5781 | 42.4155 | T1-3-R | A2063G |
| III-170 | 44,650,964 | 6,640,775,883 | 97.2647 | 92.607 | 41.6625 | Undetermined | Undetermined |
| III-172 | 58,679,732 | 8,744,239,260 | 96.7907 | 91.6552 | 41.5857 | T1-3-R | Undetermined |
| III-173 | 53,431,170 | 7,976,535,919 | 96.9348 | 91.9064 | 43.3229 | T1-3-R | Undetermined |
| III-175 | 66,949,874 | 9,988,180,291 | 96.6684 | 91.1222 | 41.3995 | T1-3-R | A2063G |
| III-176 | 60,550,792 | 9,035,381,595 | 96.8608 | 91.7563 | 42.3604 | T1-3-R | Undetermined |
| III-177 | 60,796,094 | 9,089,311,068 | 96.8371 | 91.6965 | 42.1864 | Undetermined | Undetermined |
| III-178 | 57,245,406 | 8,545,887,837 | 96.3979 | 90.6287 | 41.9177 | T1-3-R | A2063G |
| III-179 | 57,612,216 | 8,594,560,805 | 97.6091 | 93.2584 | 43.9506 | Undetermined | Undetermined |
| III-18 | 60,265,984 | 8,977,270,517 | 96.5979 | 91.0283 | 42.8593 | T1-3-R | A2063G |
| III-180 | 79,581,818 | 11,836,000,215 | 97.5241 | 93.1798 | 41.1176 | T1-3-R | Undetermined |
| III-183 | 72,845,810 | 10,868,226,969 | 96.2873 | 90.4101 | 42.3292 | T1-2 | A2063G |
| III-184 | 79,312,268 | 11,839,642,055 | 96.4166 | 90.627 | 41.8543 | T1-3-R | A2063G |
| III-185 | 68,530,586 | 10,207,025,166 | 96.9247 | 91.9487 | 41.4706 | T1-3-R | A2063G |
| III-188 | 81,889,114 | 12,222,933,548 | 96.2443 | 90.2964 | 42.3778 | T1-3-R | Undetermined |
| III-189 | 85,819,976 | 12,794,185,922 | 96.661 | 91.1368 | 41.3624 | T1-3-R | A2063G |
| III-192 | 70,370,044 | 10,496,576,771 | 96.6376 | 91.2468 | 42.1286 | T2-A | Undetermined |
| III-193 | 82,852,594 | 12,354,528,778 | 97.2496 | 92.5497 | 42.5874 | T1-3-R | A2063G |
| III-194 | 62,214,134 | 9,272,492,172 | 97.2804 | 92.6356 | 44.3383 | T1-3-R | A2063G |
| III-195 | 66,101,164 | 9,860,177,604 | 97.4144 | 92.8855 | 41.4465 | T1-3-R | A2063G |
| III-199 | 77,244,678 | 11,510,642,613 | 97.237 | 92.5418 | 41.5071 | T1-3-R | A2063G |
| III-2 | 71,295,110 | 10,620,589,086 | 96.8413 | 91.4617 | 42.1569 | T1-3-R | A2063G |
| III-200 | 59,704,374 | 8,874,046,457 | 97.4567 | 93.0959 | 42.164 | T1-3-R | A2063G |
| III-201 | 58,692,362 | 8,759,649,600 | 96.944 | 91.6116 | 42.699 | T1-3-R | Undetermined |
| III-202 | 63,166,112 | 9,424,141,199 | 97.1306 | 92.4037 | 44.9323 | T1-3-R | Undetermined |
| III-203 | 50,379,670 | 7,247,662,882 | 96.583 | 91.3071 | 42.1747 | T1-3-R | A2063G |
| III-204 | 77,353,872 | 11,492,296,181 | 96.8944 | 91.9181 | 42.9715 | T2-B | Undetermined |
| III-205 | 74,576,306 | 11,115,634,584 | 96.7884 | 91.697 | 44.868 | T1-3-R | A2063G |
| III-206 | 29,980,044 | 4,079,090,459 | 94.9132 | 88.5688 | 46.5108 | Undetermined | Undetermined |
| III-209 | 50,564,390 | 7,542,199,672 | 96.5389 | 91.2015 | 46.1274 | T2-B | Undetermined |
| III-210 | 75,472,406 | 11,176,386,759 | 96.8432 | 91.7987 | 46.7352 | T1-3-R | Undetermined |
| III-211 | 70,144,216 | 10,451,400,238 | 96.1401 | 90.4808 | 46.5455 | T1-3-R | A2063G |
| III-213 | 62,076,190 | 9,235,461,848 | 96.8844 | 91.8519 | 42.834 | T2-A | A2063G |
| III-217 | 64,835,342 | 9,681,501,447 | 97.1806 | 92.3917 | 44.4452 | T1-3-R | A2063G |
| III-218 | 69,580,964 | 10,381,929,460 | 96.8449 | 91.8219 | 45.7812 | T1-3-R | Undetermined |
| III-222 | 59,085,192 | 8,731,169,141 | 97.7472 | 93.7266 | 43.5547 | Undetermined | Undetermined |
| III-223 | 53,525,968 | 7,966,738,715 | 96.9243 | 91.933 | 41.3792 | T1-3-R | A2063G |
| III-225 | 51,006,006 | 7,615,393,613 | 96.9661 | 92.0289 | 43.0795 | Undetermined | Undetermined |
| III-226 | 55,615,130 | 8,278,871,495 | 97.2637 | 92.6822 | 45.2798 | T1-3-R | A2063G |
| III-227 | 78,140,834 | 11,624,720,559 | 97.1959 | 92.7056 | 46.9079 | T1-3-R | A2063G |
| III-228 | 74,800,960 | 11,148,396,477 | 97.2669 | 92.6351 | 41.2883 | Undetermined | Undetermined |
| III-229 | 89,746,680 | 13,364,799,118 | 97.1377 | 92.379 | 42.6879 | Undetermined | Undetermined |
| III-236 | 71,672,180 | 10,546,692,918 | 97.6575 | 93.5474 | 41.0008 | T1-3-R | A2063G |
| III-240 | 52,297,022 | 7,313,795,112 | 96.2461 | 91.065 | 44.8184 | T1-3-R | A2063G |
| III-241 | 47,565,768 | 7,066,722,237 | 96.9768 | 92.0683 | 41.9412 | T1-2 | Undetermined |
| III-28 | 54,942,990 | 8,129,797,388 | 96.6773 | 91.403 | 42.5595 | T2-A | Undetermined |
| III-31 | 48,028,870 | 7,156,798,050 | 97.2163 | 92.4696 | 41.4808 | T1-3-R | A2063G |
| III-32 | 76,349,500 | 11,369,591,953 | 96.5419 | 90.9218 | 41.8698 | T1-2 | A2063G |
| III-38 | 67,950,030 | 10,132,530,220 | 96.2552 | 90.3172 | 44.1016 | T1-3-R | A2063G |
| III-40 | 58,514,032 | 8,711,999,185 | 96.0095 | 89.8936 | 41.1171 | T1-3-R | Undetermined |
| III-46 | 53,739,812 | 8,005,557,642 | 96.8806 | 91.7945 | 42.3926 | T1-3-R | A2063G |
| III-50 | 65,504,380 | 9,760,076,250 | 97.0519 | 92.1618 | 41.4664 | T2-B | Undetermined |
| III-51 | 72,660,038 | 10,835,816,713 | 96.0072 | 89.8713 | 41.0807 | T2-A | A2063G |
| III-53 | 52,349,870 | 7,811,727,498 | 96.8788 | 91.7658 | 41.2784 | T1-3-R | Undetermined |
| III-55 | 64,032,406 | 9,537,904,300 | 96.0816 | 90.0006 | 41.3364 | T1-3-R | Undetermined |
| III-57 | 86,605,530 | 12,878,781,036 | 96.7672 | 91.6036 | 41.6832 | T2-A | A2063G |
| III-59 | 70,973,534 | 10,591,106,332 | 95.8635 | 89.5957 | 40.7637 | T1-3-R | A2063G |
| III-6 | 76,413,714 | 11,386,337,776 | 97.4136 | 92.9631 | 46.9166 | T2-B | Undetermined |
| III-62 | 52,872,458 | 7,866,072,024 | 96.9789 | 92.0776 | 41.7294 | T2-A | Undetermined |
| III-63 | 69,867,706 | 10,401,708,899 | 96.8796 | 91.8388 | 40.7445 | T1-3-R | Undetermined |
| III-65 | 53,859,922 | 8,023,506,808 | 96.7952 | 91.607 | 40.7491 | T1-3-R | A2063G |
| III-66 | 67,414,922 | 10,036,716,778 | 97.3615 | 92.7884 | 43.694 | Undetermined | Undetermined |
| III-67 | 53,337,968 | 7,952,813,175 | 96.8632 | 91.7575 | 40.7308 | T1-3-R | Undetermined |
| III-68 | 77,418,372 | 11,532,672,635 | 97.1448 | 92.3432 | 41.847 | T1-3-R | A2063G |
| III-69 | 71,722,400 | 10,703,042,162 | 96.4435 | 90.8963 | 40.9957 | Undetermined | Undetermined |
| III-7 | 62,219,996 | 9,236,855,496 | 96.9375 | 91.6601 | 41.4006 | T1-3-R | A2063G |
| III-70 | 68,447,216 | 10,114,071,472 | 96.4421 | 90.9939 | 41.5254 | T1-3-R | A2064G |
| III-71 | 71,308,396 | 10,623,493,219 | 96.209 | 90.2442 | 41.7261 | Undetermined | A2063G |
| III-72 | 64,341,398 | 9,427,866,072 | 96.6896 | 91.5728 | 42.0014 | T1-3-R | A2063G |
| III-73 | 65,290,748 | 9,734,889,131 | 96.7331 | 91.2227 | 41.6304 | T2-B | Undetermined |
| III-74 | 63,876,206 | 9,525,068,952 | 96.0586 | 89.9724 | 41.4954 | Undetermined | Undetermined |
| III-75 | 56,818,808 | 8,419,222,559 | 95.1895 | 88.4044 | 41.3694 | T1-3-R | Undetermined |
| III-76 | 59,475,000 | 8,869,351,637 | 96.9231 | 91.8476 | 41.7744 | T1-3-R | Undetermined |
| III-77 | 73,528,162 | 10,947,469,123 | 96.2544 | 90.3594 | 41.6454 | T1-3-R | Undetermined |
| III-78 | 82,572,694 | 12,321,428,773 | 96.167 | 90.1712 | 42.0155 | T2-B | Undetermined |
| III-79 | 64,113,702 | 9,570,573,417 | 96.8565 | 91.7346 | 41.4359 | Undetermined | Undetermined |
| III-80 | 96,004,376 | 14,269,674,166 | 96.6683 | 91.143 | 40.7478 | T1-3-R | A2063G |
| III-81 | 77,380,286 | 11,561,136,648 | 97.3392 | 92.6567 | 43.4844 | Undetermined | Undetermined |
| III-82 | 74,994,426 | 11,178,448,374 | 96.3246 | 90.453 | 41.2784 | Undetermined | Undetermined |
| III-83 | 71,559,204 | 10,684,506,721 | 96.4262 | 90.6415 | 40.436 | Undetermined | Undetermined |
| III-84 | 79,486,408 | 11,875,950,800 | 96.2051 | 90.5143 | 43.7265 | Undetermined | A2063G |
| III-85 | 59,027,196 | 8,816,160,916 | 96.4727 | 90.9374 | 42.169 | T1-3-R | Undetermined |
| III-86 | 112,619,450 | 16,815,314,451 | 96.2979 | 90.441 | 41.105 | T1-3-R | A2063G |
| III-87 | 71,272,826 | 10,641,359,606 | 96.8759 | 91.8262 | 42.7366 | T1-3-R | Undetermined |
| III-88 | 53,000,896 | 7,909,341,360 | 96.9668 | 92.0178 | 43.7443 | T1-3-R | A2063G |
| III-89 | 100,852,044 | 15,042,643,835 | 96.1751 | 90.3969 | 41.6413 | Undetermined | Undetermined |
| III-90 | 45,289,588 | 5,747,669,959 | 93.225 | 85.5044 | 48.4441 | Undetermined | Undetermined |
| III-91 | 35,609,764 | 5,249,726,172 | 96.1968 | 90.5649 | 42.0325 | Undetermined | Undetermined |
| III-92 | 56,761,792 | 8,448,135,550 | 96.8268 | 91.657 | 41.0841 | T1-3-R | Undetermined |
| III-93 | 94,886,746 | 14,133,501,268 | 97.0535 | 91.9319 | 41.0606 | Undetermined | Undetermined |
| III-94 | 53,236,480 | 7,944,621,692 | 96.6003 | 91.2247 | 42.209 | T1-3-R | A2063G |
| III-95 | 32,768,182 | 4,876,036,951 | 96.5877 | 91.2191 | 43.4236 | Undetermined | Undetermined |
| III-96 | 28,718,216 | 4,260,735,441 | 96.3788 | 90.7916 | 41.8504 | T1-3-R | Undetermined |
| III-97 | 74,265,516 | 11,055,772,604 | 97.612 | 93.3665 | 41.5971 | T1-3-R | A2063G |
| III-98 | 52,187,408 | 7,781,346,013 | 96.9063 | 91.7866 | 41.9813 | T2-A | Undetermined |
| III-99 | 58,164,072 | 8,666,294,285 | 96.4263 | 90.8883 | 41.9698 | T1-3-R | A2063G |
| IV-1 | 86,662,370 | 12,841,986,739 | 98.6848 | 95.8101 | 59.8075 | Undetermined | Undetermined |
| IV-10 | 75,292,510 | 10,995,451,796 | 98.6544 | 95.7583 | 60.8039 | Undetermined | Undetermined |
| IV-11 | 108,822,162 | 15,865,429,295 | 98.5396 | 95.4723 | 50.3913 | Undetermined | A2067G |
| IV-12 | 87,586,312 | 12,368,284,170 | 98.02 | 94.5883 | 45.7514 | Undetermined | Undetermined |
| IV-13 | 102,660,948 | 14,935,564,046 | 98.9285 | 96.5738 | 45.6583 | T2-B | Undetermined |
| IV-14 | 124,903,726 | 17,919,971,302 | 98.9188 | 96.593 | 41.5325 | T1-3-R | A2063G |
| IV-15 | 111,043,654 | 14,982,876,689 | 98.6991 | 96.222 | 47.8815 | T1-3-R | Undetermined |
| IV-16 | 78,815,550 | 10,571,358,722 | 98.2596 | 95.1416 | 45.8509 | Undetermined | Undetermined |
| IV-17 | 91,325,606 | 13,369,036,429 | 98.6993 | 95.9057 | 58.3113 | T1-3-R | Undetermined |
| IV-18 | 84,532,360 | 12,121,099,085 | 98.8448 | 96.4055 | 47.6167 | Undetermined | Undetermined |
| IV-19 | 90,671,326 | 13,228,870,706 | 98.7517 | 96.115 | 55.8197 | T1-3-R | A2063G |
| IV-2 | 120,179,334 | 17,594,511,520 | 98.5884 | 95.6458 | 53.8279 | T1-3-R | Undetermined |
| IV-20 | 82,775,308 | 12,110,174,347 | 98.6851 | 95.8742 | 51.9675 | T1-3-R | A2063G |
| IV-21 | 84,357,238 | 12,149,210,903 | 99.005 | 96.8625 | 42.0651 | Undetermined | Undetermined |
| IV-22 | 98,464,272 | 13,968,480,750 | 98.8205 | 96.2763 | 44.8606 | T1-3-R | Undetermined |
| IV-23 | 94,629,228 | 13,820,831,482 | 98.8225 | 96.2563 | 47.9285 | T1-3-R | Undetermined |
| IV-24 | 126,179,012 | 18,575,738,591 | 98.5332 | 95.4915 | 55.974 | T1-3-R | Undetermined |
| IV-25 | 122,156,982 | 17,654,359,925 | 98.5974 | 95.7094 | 57.9048 | T2-A | A2063G |
| IV-26 | 131,189,836 | 18,847,001,716 | 98.7942 | 96.2544 | 48.3196 | Undetermined | Undetermined |
| IV-27 | 82,374,012 | 12,085,573,705 | 98.8399 | 96.3609 | 46.8624 | T1-3-R | Undetermined |
| IV-28 | 69,858,266 | 9,750,973,959 | 98.1881 | 94.8645 | 44.7458 | Undetermined | Undetermined |
| IV-29 | 93,736,838 | 13,661,102,913 | 98.4635 | 95.2044 | 56.626 | T2-B | A2063G |
| IV-3 | 71,911,060 | 10,585,343,271 | 98.6823 | 95.8798 | 56.3658 | Undetermined | Undetermined |
| IV-30 | 80,500,904 | 11,881,045,977 | 98.5446 | 95.4367 | 56.7524 | Undetermined | Undetermined |
| IV-31 | 118,076,018 | 17,339,216,392 | 98.8589 | 96.3859 | 41.9377 | Undetermined | Undetermined |
| IV-32 | 155,980,870 | 22,204,262,941 | 98.9217 | 96.5705 | 42.7144 | T1-3-R | Undetermined |
| IV-33 | 100,503,346 | 14,896,757,675 | 98.5112 | 95.2982 | 60.4136 | Undetermined | Undetermined |
| IV-34 | 112,297,772 | 15,374,161,055 | 98.1346 | 94.7721 | 45.3076 | Undetermined | Undetermined |
| IV-35 | 99,653,234 | 14,539,168,531 | 98.6458 | 95.7305 | 47.8332 | T1-3-R | Undetermined |
| IV-36 | 141,991,434 | 21,001,616,445 | 98.5694 | 95.4903 | 52.276 | T1-3-R | Undetermined |
| IV-37 | 107,746,620 | 15,797,435,352 | 98.6207 | 95.7371 | 59.4099 | Undetermined | Undetermined |
| IV-38 | 112,925,830 | 16,131,616,315 | 99.0642 | 96.9305 | 43.1079 | Undetermined | Undetermined |
| IV-39 | 154,727,462 | 22,676,702,698 | 98.9876 | 96.6761 | 55.5483 | Undetermined | Undetermined |
| IV-4 | 72,868,468 | 10,691,853,127 | 98.4369 | 95.1731 | 55.9953 | Undetermined | Undetermined |
| IV-40 | 103,348,774 | 14,964,211,410 | 98.6214 | 95.657 | 51.1911 | T1-3-R | A2063G |
| IV-41 | 115,834,794 | 16,997,911,533 | 98.6306 | 95.5415 | 52.3607 | T1-3-R | A2063G |
| IV-42 | 134,769,080 | 18,940,076,228 | 98.2724 | 95.0399 | 46.2274 | Undetermined | Undetermined |
| IV-43 | 151,515,678 | 20,721,653,569 | 98.5544 | 95.7421 | 47.1898 | T1-3-R | Undetermined |
| IV-44 | 99,893,730 | 14,675,355,444 | 98.7641 | 96.0704 | 41.6148 | Undetermined | Undetermined |
| IV-45 | 76,337,660 | 10,421,409,246 | 97.87 | 94.2246 | 46.3453 | Undetermined | Undetermined |
| IV-46 | 72,463,384 | 10,548,030,627 | 98.6663 | 95.8539 | 42.1886 | T1-3-R | Undetermined |
| IV-5 | 79,614,642 | 11,711,863,002 | 98.4904 | 95.2728 | 52.2061 | T1-3-R | Undetermined |
| IV-6 | 72,635,132 | 10,627,858,385 | 98.8042 | 96.2349 | 47.7939 | Undetermined | Undetermined |
| IV-7 | 78,592,122 | 11,522,591,700 | 98.6572 | 95.8106 | 50.4655 | T1-3-R | Undetermined |
| IV-8 | 134,458,246 | 19,963,677,394 | 98.3819 | 94.9515 | 58.7967 | Undetermined | Undetermined |
| IV-9 | 76,315,812 | 10,782,792,451 | 98.0995 | 94.8009 | 45.2569 | Undetermined | Undetermined |

Samples beginning with I, II, and III are the 205 samples collected in August 2023; samples beginning with IV are the 46 samples collected in May 2024. "Undetermined" indicates that the sequencing results did not cover the sites used for clade and MR mutation detection, making it impossible to determine the clade and MR mutation status.


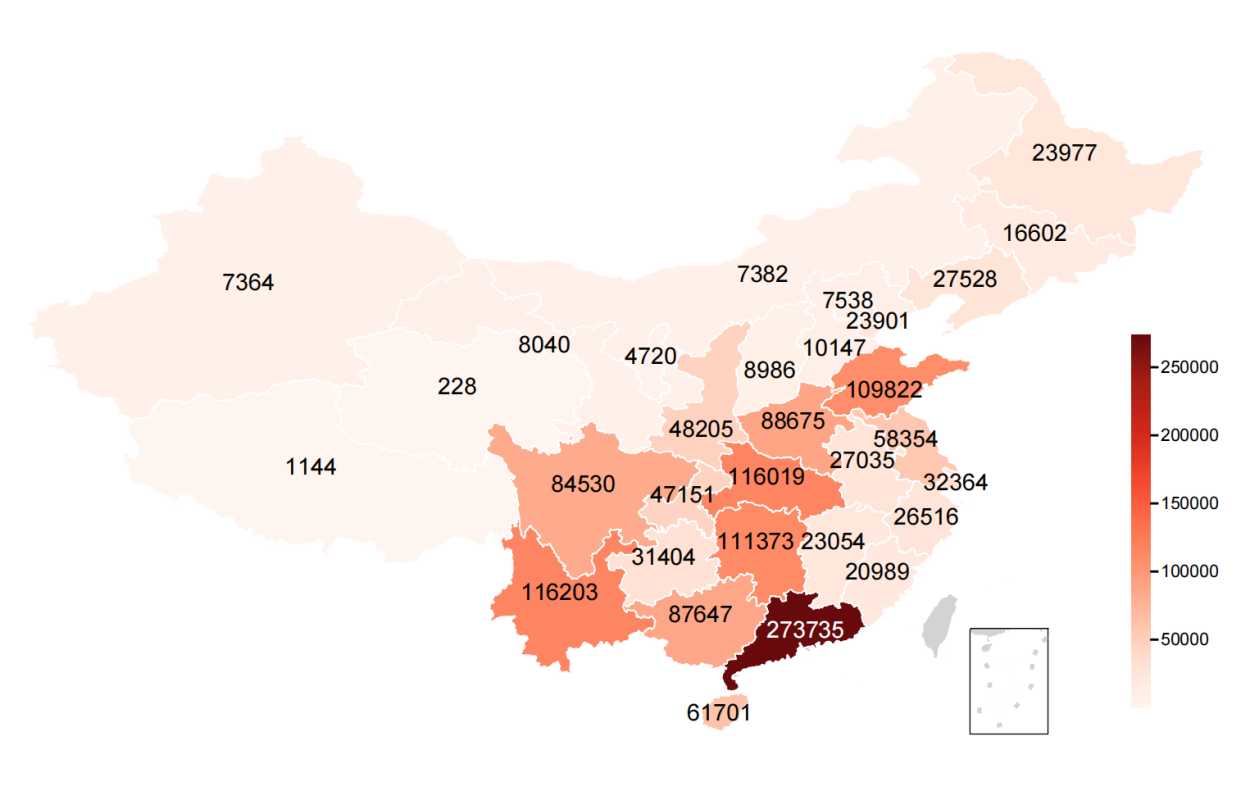


**Figure S1. Geographical distribution of ARI case in the study**


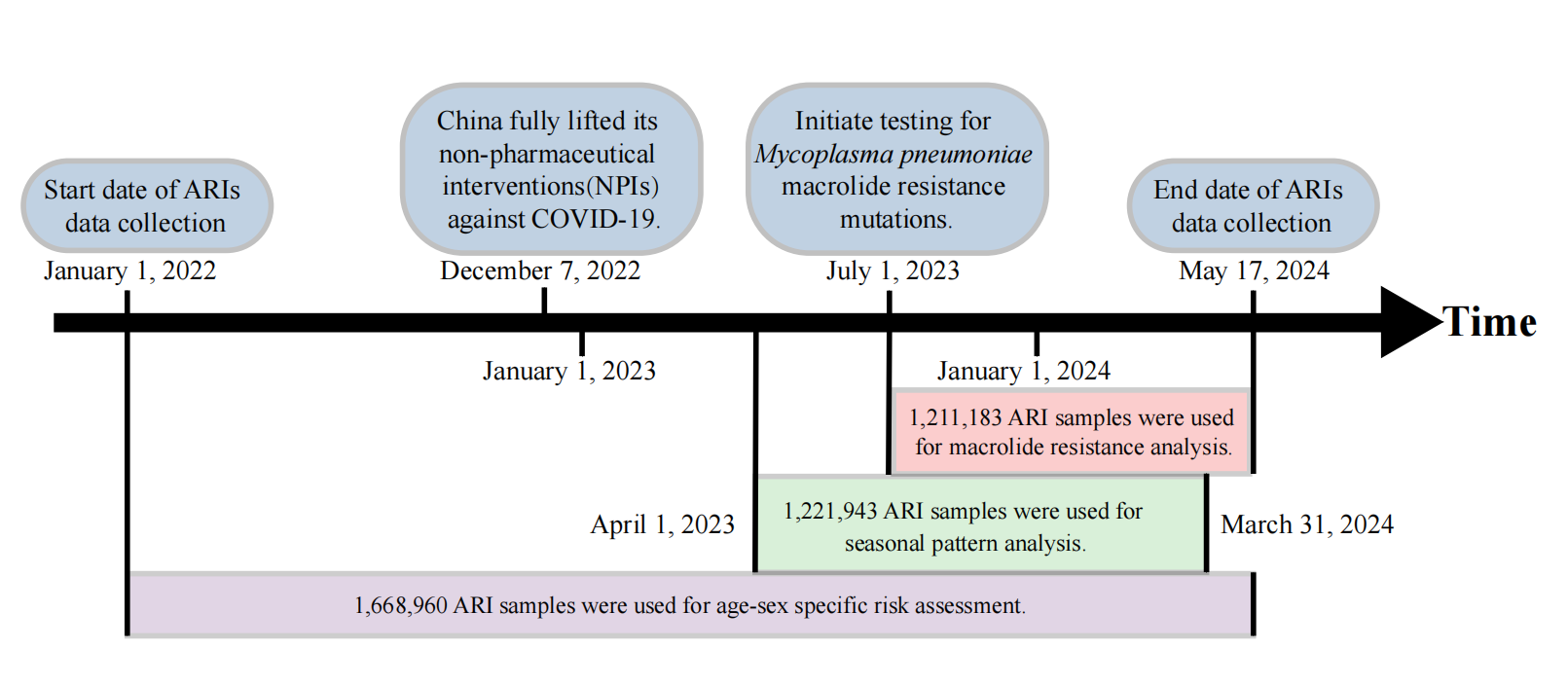


**Figure S2. A schematic diagram of the tNGS datasets selected for different analyses, with key time points marked above the timeline. All data were used for sex- and age-specific infection risk analysis. On December 7, 2022, China fully lifted its Non-Pharmaceutical Interventions (NPI). From January to March 2023, the Chinese population experienced a peak in COVID-19 infections, which affected the infection and co-infection patterns of other respiratory pathogens. To avoid the impact of COVID-19, we selected the period from April 2023 to March 2024 as the annual data for seasonal pattern analysis. Monitoring of MP resistance rates began on July 1, 2023, so we selected cases from July 2023 to May 2024 for the MP resistance study.**


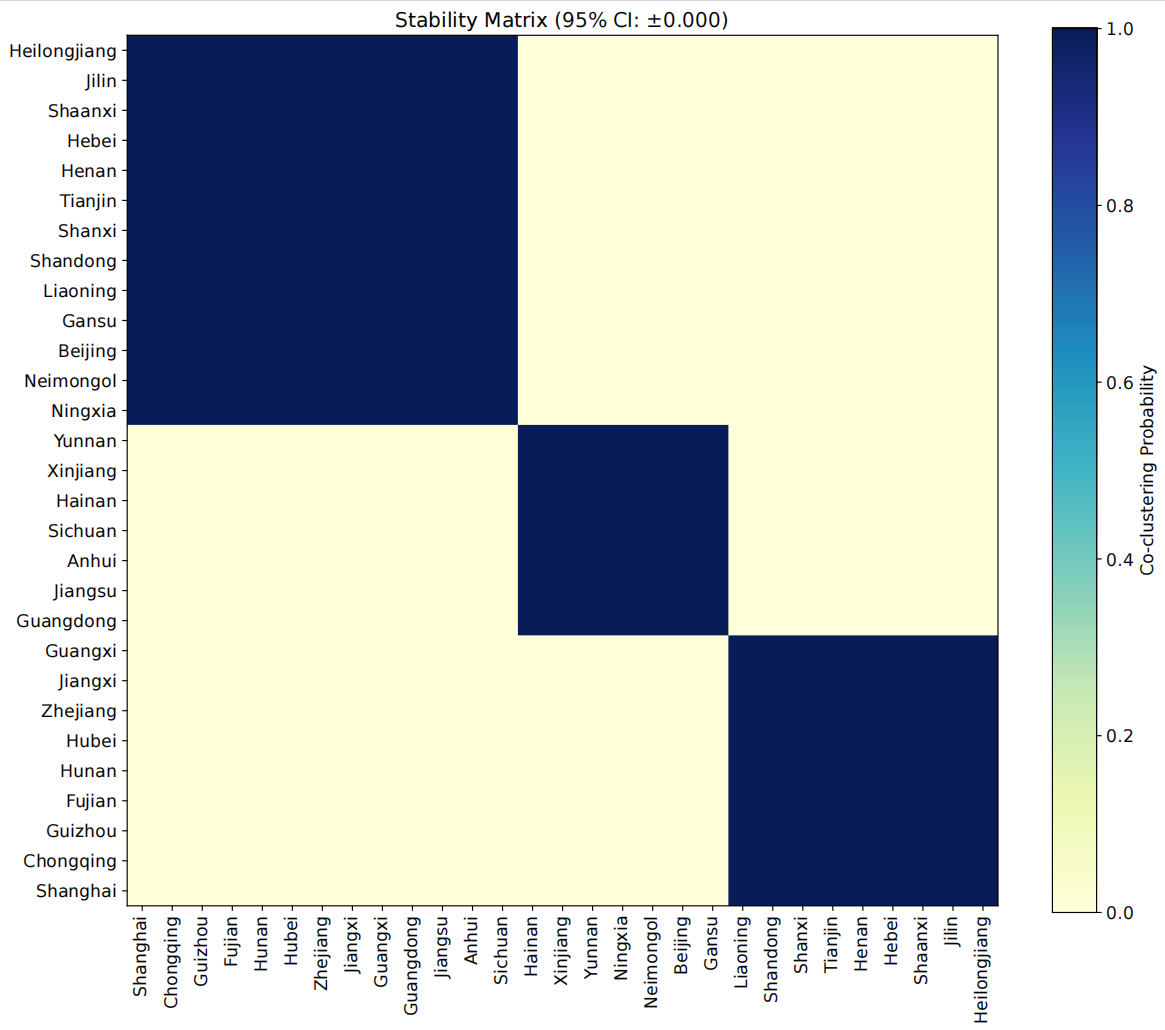


**Figure S3 Inter-provincial clustering group stability matrix**


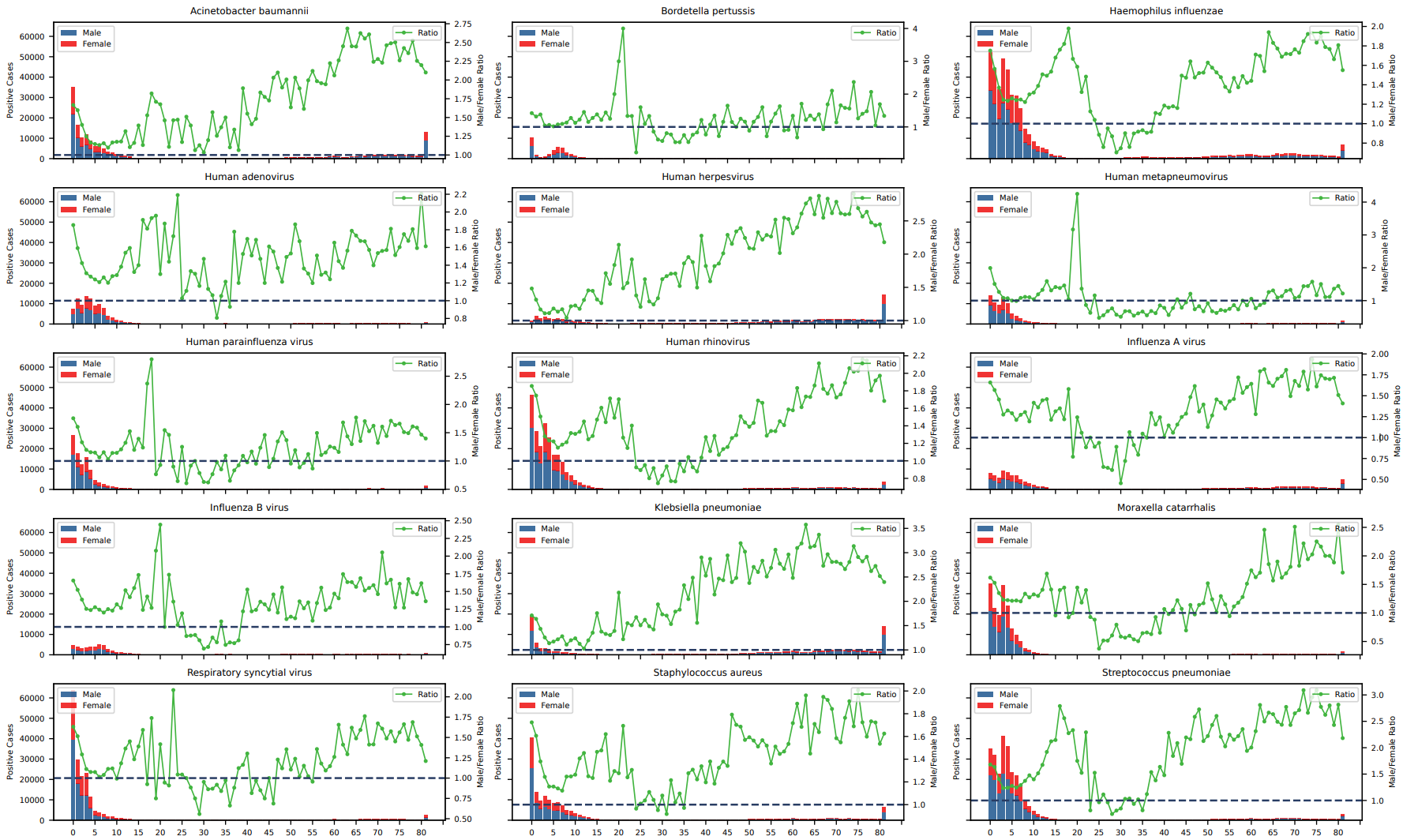


**Figure S4. Comparative analysis of 15 pathogens infection risk between males and females. The x-axis represents age. The red bars denote the number of female cases, while the blue bars denote male cases. The green line represents the risk ratio of pathogen infection between males and females**


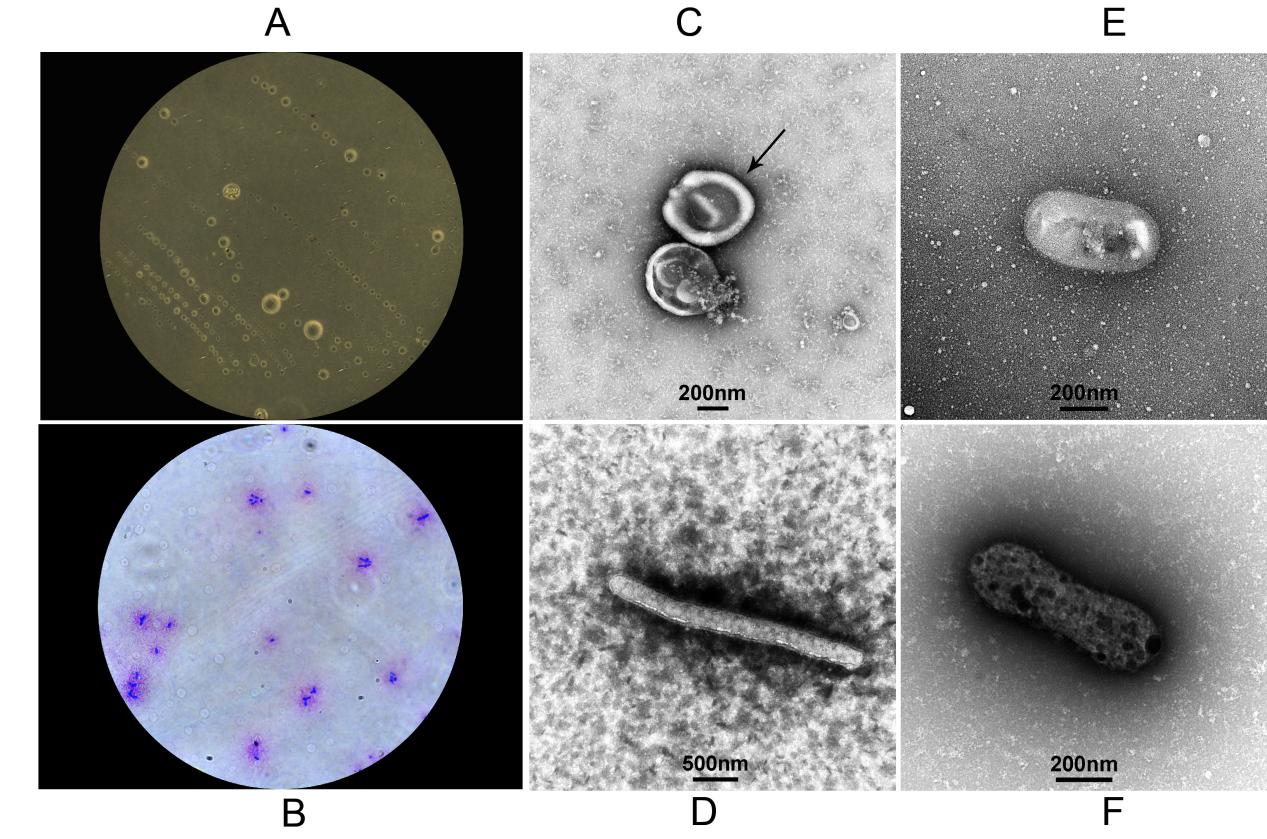


**Figure S5. The morphology of isolated outbreak strain of MP**

**A: The typical "fried egg" colony morphology under light microscope (×400).**

**B: The cells stained by Giemsa's dye.**

**F: The pleomorphic morphology of MP with negative staining under transmission electron microscopy.**

**#: The MP particles present spherical (with arrow in Fig C), filamentous (Fig D), ovoid (Fig E) or stick-shaped (Fig F).**


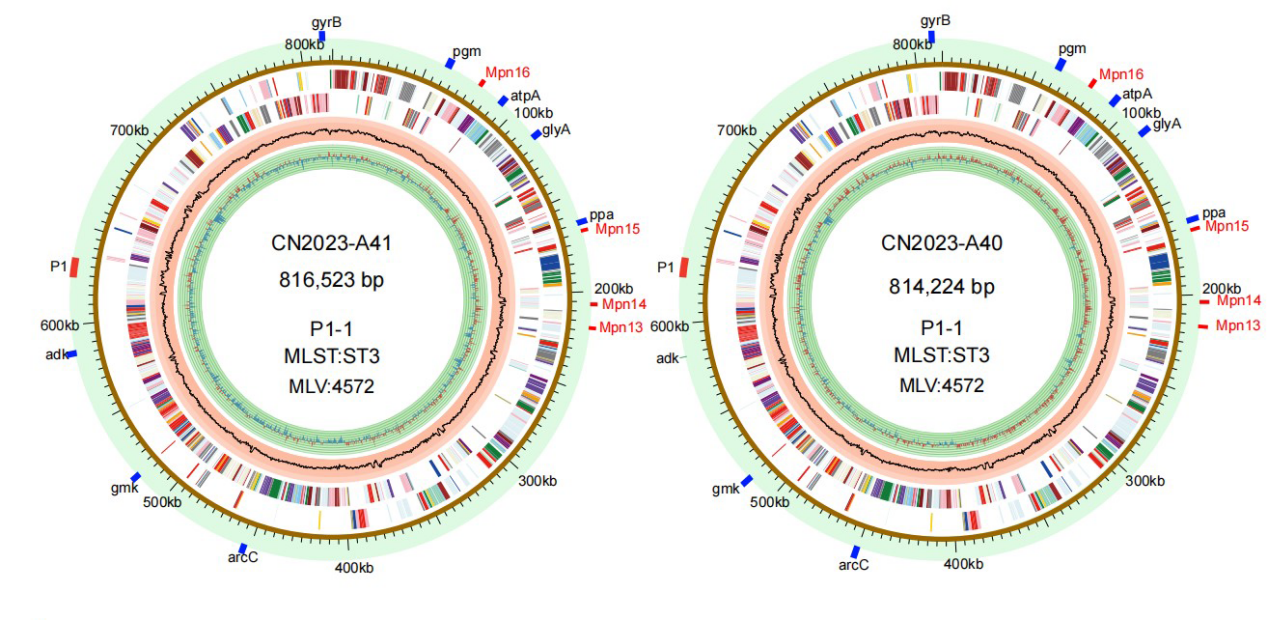


**Figure S6. The genome Circos Plot of strain CN2023-A40 and CN2023-A41 and genome.**

**#: The outermost circle annotated for the p1 gene used for P1 typing; the four genes (Mpn13, Mpn15, Mpn16, Mpn17) used for MLVA typing; and the eight genes (adk, gmk, arcC, ppa, glyA, atpA, pgm, gyrB) used for MLST typing. The second and third circles from the outermost represent genes on the positive and negative strands, respectively, with different colors indicating various functional genes. The fourth circle represents the GC content, while the fifth circle represents the GC skew. The innermost circle provides text annotations for the sizes of the two genomes and the results of P1, MLST, and MLVA typing.**
